# Water impacts of U.S. biofuels: Insights from an assessment combining economic and biophysical models

**DOI:** 10.1371/journal.pone.0204298

**Published:** 2018-09-28

**Authors:** Jacob Teter, Sonia Yeh, Madhu Khanna, Göran Berndes

**Affiliations:** 1 International Energy Agency, Sustainable Technology Outlooks, Paris, France; 2 Department of Space, Earth and Environment, Chalmers University of Technology, Gothenburg, Sweden; 3 Department of Agricultural and Consumer Economics, University of Illinois at Urbana Champaign, Urbana, Illinois, United States of America; 4 DOE Center for Advanced Bioenergy and Bioproducts Innovation, University of Illinois at Urbana-Champaign, Urbana Illinois, United States of America; University of Huddersfield, UNITED KINGDOM

## Abstract

Biofuels policies induce land use changes (LUC), including cropland expansion and crop switching, and this in turn alters water and soil management practices. Policies differ in the extent and type of land use changes they induce and therefore in their impact on water resources. We quantify and compare the spatially varying water impacts of biofuel crops stemming from LUC induced by two different biofuels policies by coupling a biophysical model with an economic model to simulate the economically viable mix of crops, land uses, and crop management choices under alternative policy scenarios. We assess the outputs of an economic model with a high-resolution crop-water model for major agricultural crops and potential cellulosic feedstocks in the US to analyze the impacts of three alternative policy scenarios on water balances: a counterfactual ‘no-biofuels policy’ (BAU) scenario, a volumetric mandate (Mandate) scenario, and a clean fuel-intensity standard (CFS) scenario incentivizing fuels based on their carbon intensities. While both biofuel policies incentivize more biofuels than in the counterfactual, they differ in the mix of corn ethanol and advanced biofuels from miscanthus and switchgrass (more corn ethanol in Mandate and more cellulosic biofuels in CFS). The two policies differ in their impact on irrigated acreage, irrigation demand, groundwater use and runoff. Net irrigation requirements increase 0.7% in Mandate and decrease 3.8% in CFS, but in both scenarios increases are concentrated in regions of Kansas and Nebraska that rely upon the Ogallala aquifer for irrigation water. Our study illustrates the importance of accounting for the overall LUC and shifts in agricultural production and management practices in response to policies when assessing the water impacts of biofuels.

## Introduction

Policy support for biofuels motivated by the objectives of energy security and greenhouse gas (GHG) mitigation have led to substantial expansion of biofuel production in the last decade in the US [1, 2]. Several studies have examined the land use and GHG implications of biofuel policies. These studies show that policies promoting biofuels production lead to significant changes in cropping patterns by inducing crop switching (or “displacement”) and expansion of cropped area (or “extensification”)[3–6] as well as altering water/soil management practices (e.g. irrigation, tillage)[4, 7–10]. Studies also show that the volume and mix of biofuels and biofuel feedstocks and thus their land use and GHG implications differs considerably across alternative biofuel policies [11, 12]. There has however been limited assessment of the implications of alternative biofuel policies for water resources. As the allocation of water resources is a key concern of natural resource management, particularly in regions where they are scarce, and since changes in land use can directly impact sub-surface water availability for other economic uses, groundwater recharge, and in-stream ecology, it is important to try to understand the implications of different biofuels policies on water availability.

Biofuel feedstocks differ in the amount and type of water required for their production. Food crop based biofuel feedstocks (corn and soybeans) are grown under both rainfed and irrigated conditions in the US while, in accordance with the assumptions in the economic model, energy crops for cellulosic biofuels are expected to be grown under rainfed conditions. However, cellulosic energy crops have high evapotranspiration rates; as a result despite their higher water use efficiency compared to corn, they also have high non-irrigation water requirements with potentially adverse impacts on sub-surface water flows.

Various approaches have been suggested in the literature for assessing the water use impacts of biofuel supply chains, including water-use lifecycle assessment (WU-LCA) and water footprint (WF) methods [13–20] The WF method assesses the volumes of ‘green’ (i.e. soil water sourced from precipitation), ‘blue’ (irrigation), and in certain instances also ‘gray’ water (which is an estimate of the volume of water required to dilute water polluted by e.g. nitrates, phosphates, etc. to given threshold levels) needed to produce a megajoule of biofuels [21–25]. Geographically explicit WF assessments find that the WF of cultivating biofuel feedstocks such as corn stover and grain, and wheat straw vary by more than an order of magnitude across US counties due to spatial heterogeneity in water use and due to variations in the methods used for the assessment [26]. WU-LCA studies differ in their approach to characterizing the impacts of biofuel production pathways on water resource quality and availability; some focus on a particular step or portion of the biofuels production pathway while others undertake a full ‘well-to-wheels’ assessment of all steps of biofuel production and use [13, 17, 18, 26–28]. Different methods of allocating water along the production supply chain (such as allocating water to feedstocks based on mass [26] or the entire crop [29], or using system expansion to account for co-products water use) can result in very different conclusions [16, 30]. In addition, as biofuel supply chains can extend over large distances, ascertaining impacts on water resource quality and availability from representative LCAs can be misleading.

Existing studies using WU-LCA and WF approaches have not incorporated the effects of economically viable changes in land use and land management due to policies promoting biofuels production. Since different policy regimes may alter cropping and other land use patterns, attempts to grasp the regional water use impacts of *policies* should be used to inform policy makers on the natural resource implications of various alternatives. Exceptions to this include Housh et al. [31], which considers the effects of perennial feedstocks in a single watershed in Illinois. Vanloocke et al. [32] examines the effects of converting various exogenously given shares of cropland hectares to miscanthus and switchgrass in the Mississippi River Basin without considering the economic incentives needed for that land conversion. Due to the lack of a comparison with a counterfactual scenario (i.e. what would have happened in the absence of biofuels policies?) and without consideration of the dynamic interactions between crops production decisions, policy-incentivized land use, management practices and crop choices, the reported biofuel life-cycle water use, or WFs, do not provide sufficient information for making conclusions about potential water impacts of alternative biofuel policies. They, therefore, have limited value for policy makers that want to compare the potential impacts of alternative policy regimes and develop effective measures to promote positive and mitigate negative impacts. To quantify the spatially varying water impacts of biofuel crops, it is important to couple biophysical models with an economic model to simulate the economically viable, spatially varying mix of crops, land uses, and crop management choices under alternative policy scenarios [33].

This study shows how these shortcomings can be addressed by adopting a scenario-based approach that combines land use outcomes from a U.S.-scale economic model (BEPAM) with a crop-water model (CropWatR) of major crops and biofuel feedstocks. The contribution of this study is to analyze water use effects of crop switching and land use change (LUC) of two alternative policy scenarios considered in Chen et al. [11], a biofuel mandate and a clean fuel-intensity standard. The policy scenarios modeled in Chen et al. focus on the period 2007–2030. The policies analyzed here are stylized versions of the Renewable Fuel Standard and the California Low Carbon Fuel Standard, extended to a national scale [34]. These two policies differ in the mix of feedstock production and the spatial pattern of land use changes they induce, and were chosen because they are the main biofuel policies implemented in the US (Mandate) and in states including California and Oregon and had been proposed for the national scale (CFS) [12, 35]. Our focus here is to estimate differences in seasonal and annual water flow balances among the crop, soil, and atmosphere between these two alternative policies rather than on analyzing the feasibility and implementability of these policies over the time horizon considered. The two policy scenarios analyzed here were developed to achieve the same cumulative GHG reduction targets over this time period. By comparing the water impacts of these policies, our analysis shows the different impacts that the same GHG mitigation target can pose on water resource use and availability and the potential to mitigate these impacts through policy choices.

## Data and methods

Three scenarios are examined in this study: (1) a counterfactual scenario (BAU) in which we assume that there is no biofuel policy; (2) a volumetric mandate scenario that simulates a stylized version of the Renewable Fuels Standard (Mandate) that sets quantity targets for different types of biofuels [34]. This scenario is modeled with a target of 150 billion ethanol equivalent liters of biofuel at the end of the modeling period (in 2030) with an upper limit of 56 billion liters of corn ethanol following projections by the Annual Energy Outlook (2010) [36], and (3) a Clean Fuel-Intensity Standard (CFS) scenario that has features similar to the Low Carbon Fuel Standard (LCFS) implemented in California, Oregon, British Columbia [37] and has been proposed for the US [38, 39] and recently Canada [40]. The policy sets an increasingly stringent GHG intensity target for transportation fuels over the 2015–2030 time period and incentivizes fuels that have lower carbon intensity. In general, cellulosic ethanol has lower carbon intensity (gCO_2_e/MJ) compared with corn ethanol, making it more attractive under a CFS policy [41]. The volumetric mandate (Mandate scenario) is based on projections of the capacity to supply the required quantity of advanced biofuels by 2030 and the projected availability of flex-fuel cars to consume it by the Annual Energy Outlook [36]. To make the effects of Mandate and CFS comparable, the stringency of the GHG intensity target in CFS is calibrated to achieve the same cumulative reduction in domestic greenhouse gas emissions as the Mandate scenario by 2030. We find that an annual target for lowering GHG intensity of fuel under the CFS that increases linearly to 8% by 2030 achieves the same 4.2% reduction in cumulative domestic GHG emissions by 2030 as the Mandate scenario relative to the no-policy baseline.

Table 1 shows the volumes of biofuels produced under each scenario.

**Table 1:** Biofuels produced (billion liters) under BEPAM scenarios in the base year and at the end of the modelling period.

Biofuels pathway (Year)	Base year (2007)	BAU (2030)	Mandate (2030)	CFS (2030)
Corn ethanol	15.59	14.60	56.78	26.10
Forest waste ethanol	--	--	6.30	--
Pulpwood ethanol	--	--	0.69	--
Cellulosic ethanol (from switchgrass, miscanthus, corn stover and wheat straw)	--	--	83.52	93.20
*Total ethanol*	15.59	*14*.*60*	*147*.*29*	*119*.*30*
DDGS oil diesel	--	0.65	2.53	1.16
Soy oil diesel	--	--	0.54	--
BTL biodiesel (from forest residue)	--	--	--	3.38
Waste grease diesel		--	0.35	0.35
*Total biodiesel*	--	*0*.*65*	*3*.*43*	*4*.*90*

The main differences between the two policy scenarios are the volumes of biofuels from different categories (more corn in Mandate and more cellulosic biofuels in CFS), land use change patterns (i.e. crop substitution and expansion, see further discussion below), management practices and variations in crop-specific characteristics, where water use efficiency, rooting profile, and length of growing season are among key characteristics. The two cellulosic energy crops included (switchgrass and miscanthus), by virtue of being perennials, have substantially longer growing seasons than row crops (which were historically grown as agricultural commodities, rather than as biofuel feedstocks), such as corn, soy, and wheat, and have similar crop-water use characteristics (and hence, similar influence on hydrology) as uncropped perennial grass land cover (such as grassland pasture or idle cropland). The energy crop cultivations however have greater transpiration rates than the marginal perennial grass land types they are typically modeled as displacing. Studies have found that perennial energy crops for cellulosic (‘second generation’) biofuels can be grown under rainfed conditions and have higher water use efficiency [42, 43], lower nutrient loading [32, 44], and surface runoff [45–48] as compared to corn. However, these crops have high yields, high transpiration rates and a longer growing season which means high water consumption per hectare (but with much less water consumption per MJ of biofuel product) [43, 49]. Wide-scale adoption of highly productive perennial feedstocks on previously sparsely vegetated lands may substantially reduce sub-surface water availability for other economic uses, groundwater recharge, in-stream ecology, etc.

We assess the water use impacts of three scenarios by combing the results of land use and land use change and agricultural management practices from the agro-economic model Biofuel Environmental Policy Analysis Model (BEPAM) [11] with a process-based crop-water modeling CropWatR [50]. In this section we briefly describe the BEPAM model and the estimated land use and agricultural management practices. The CropWatR model is described in Section 2.3.

### BEPAM model

Results from the economic model BEPAM formed the basis of crop-water modeling. BEPAM is a dynamic multi-market, multi-period partial equilibrium cost minimization model of the food, feed, and fuel sectors that has been used extensively to evaluate and compare the social welfare, greenhouse gas (GHG) emissions, land use, and economic impacts of various policies [11, 51–54]. The BEPAM model endogenously determines the cropped area for 15 crops, under various rotation (including corn-soybean, continuous corn, and fallow-wheat rotations), tillage (including conventional till and no-till, or alternating between the two) and irrigated/rainfed practices, annually from 2007 to 2030 at a crop reporting district (CRD) level. A CRD is an aggregation of typically 6–10 counties and is the basic unit of agricultural census data aggregation. In the Mandate and CFS scenarios, corn grain and soybean oil are the feedstocks for conventional ethanol and biodiesel respectively, while residues, from corn and wheat, and energy crops, switchgrass (*Panicum virgatum* L.) and miscanthus (*Miscanthus giganteus*), are potential feedstocks for producing cellulosic biofuels from 2015 onwards in the Mandate and CFS scenarios [34]. BEPAM assumes yield increases following historical trends of 1.17% and 0.54% per year for corn and soybeans, respectively [55]. The model includes several types of land, namely regular cropland, idle land, cropland pasture, pasture land, and forestland pasture, for each CRD. Land under conventional crops is assumed to change in response to crop prices, using estimated crop-specific price elasticities of acreage and price elasticity of total acreage. Idle land and cropland pasture are assumed to be available for conversion to conventional crop or energy crop production. Other land, including pasture land and forestland pasture are fixed at 2007 levels while land enrolled in the Conservation Reserve Program, is fixed at levels authorized by the Farm Bill of 2008 [56]. Annual yields of row crops are assumed to increase over time following historical trends, based on econometric analysis [55].

Energy crops can be planted either on ‘prime’ cropland or on ‘cropland pasture’ (land that is in and out of cropland and pasture/grazing and not under continuous crop production) and are assumed in all cases to be cultivated under rainfed conditions without irrigation. These energy crops are perennial grasses that cannot survive in the dry conditions to the west of the 100^th^ meridian [57]. Although these energy crops can be cultivated under irrigated conditions in Western US [58] doing so will divert water and land from food/feed crops [59]. This would raise costs of production as well as adversely affect the energy intensity and carbon intensity of these energy crops since energy would be needed to pump the groundwater or transport surface water for these crops. There has, therefore, been very limited analysis of the potential for growing these crops under irrigated conditions in the Western US, and of the amount of water that would be required and the crop yields that could be obtained under irrigated conditions. We therefore focus on the impacts of growing these crops under rainfed conditions.

### Land use change

As stated earlier, the BEPAM model results from the study Chen et al. [11] simulate differences in land use impacts incentivized by diverging policy frameworks—these results form the basis for differences in crop water balances. The main differences between the two policy scenarios are the volumes and types of biofuels: 56.8 vs. 26.1 billion liters of corn ethanol in Mandate vs. CFS, and 83.5 vs. 93.2 billion liters cellulosic ethanol in Mandate vs. CFS (Table 1). The land use patterns simulated by BEPAM at the resolution of CRDs show larger corn areas in Mandate and CFS scenarios than in the BAU scenario (Table B in S1 File) [11]. Increased cropping of row crops and energy crops is concentrated in the Midwest and Great Plains states under both scenarios—Oklahoma, Kansas, Nebraska, and North and South Dakota. More moderate increases in cropped land area occur throughout the Corn Belt. The increase in corn acreage is partially offset by decreases in the acreage under winter wheat and soybeans, and to a lesser extent by the reduction in acreage under alfalfa and other crops (Figs C-D in S1 File). The net effect is an increase in total cultivated acreage in each scenario; total cropped land increases by 2% and 6% under Mandate and CFS, respectively, compared to the BAU. In Mandate scenario, row crops (primarily corn) make up most of the increased acreage, while energy crops account for most of the increase in CFS.

In CFS, there is a far greater increase in land dedicated to switchgrass cultivation throughout Texas (Fig E in S1 File), and to miscanthus throughout the eastern U.S. (in particular in Oklahoma and along the mid stretches of the Mississippi river), compared to the Mandate scenario. The percent of ‘prime’ cropland and cropland pasture land types displaced by energy crops under Mandate and CFS is shown in Fig E in S1 File. As shown also in Table B in S1 File, most of land extensification in Mandate is due to miscanthus, which is mostly cultivated on cropland pasture (~1.5 million hectares, most pronounced in Oklahoma) and to a smaller extent on prime cropland (~0.45 million hectares). The extent of land dedicated to energy crops is far wider under CFS. Nearly four times as much cropland pasture (~5.2 million hectares) and similar areas of cropland (~5.5 million hectares) is converted to miscanthus. Switchgrass displaced a total of 1.5 million hectares, two-thirds of which is cropland pasture. In CFS, land under energy crops stretches across much of the Northern and Southern Great Plains (Colorado, New Mexico, South Dakota, Nebraska, Kansas, Oklahoma, and Texas), Midwest, and spreads across most growing regions east of the Rockies. Oklahoma, Texas, and regions along the mid- to lower-Mississippi experience the greatest concentration of energy crop cultivation. More detailed results on land use patterns in the three scenarios can be found in S1 File.

### The *CropWatR* model

The CropWatR model is an open-source GIS implementation [50] for *R* [60] of the FAO 56 Penman-Monteith dual-crop coefficient estimation of crop-water balances [61]. CropWatR performs process-based crop-water modeling at a daily time-step and high spatial resolution (10 km by 10 km).

CropWatR begins by deriving reference evapotranspiration (ET_0_) on the basis of daily weather data—it requires inputs of precipitation; minimum, mean, and maximum temperature; minimum and maximum relative humidity; wind speed at 2 meters height; water vapor pressure; and incident shortwave radiation). Daily ET_0_ derived on this bases is next combined with stage-specific crop specific parameters (including stage-specific basal crop coefficients K_cb_; plant stage lengths (in days or ratios); maximum plant height; base, mean, and maximum rooting depths; and p-value) to estimate the potential evapotranspiration as a function of the weather and the development stage of the crop. Crop development can further be specified by the user, based for instance on site specific information on cropping and harvesting dates and management regimes (i.e. irrigation and tillage). Planting dates and management rules can be either provided in raster format or specified by the user according to certain rules, such as when available soil water depletion thresholds are exceeded, for instance. The final key input needed to derive soil water balances is a characterization of the soil type. Topsoil (i.e. for the top 10 cm of soil) texture (percentage soil, silt, and sand) percentage data in raster format are used by CropWatR to calculate total evaporable water (TEW) and readily evaporable water (REW). Soil texture of the top meter is used to calculate soil water content at field capacity (q_FC_) and at wilting point (q_WP_), as well as the depth of the soil surface layer that is subject to evaporative drying (Z_e_).

**Fig 1:**
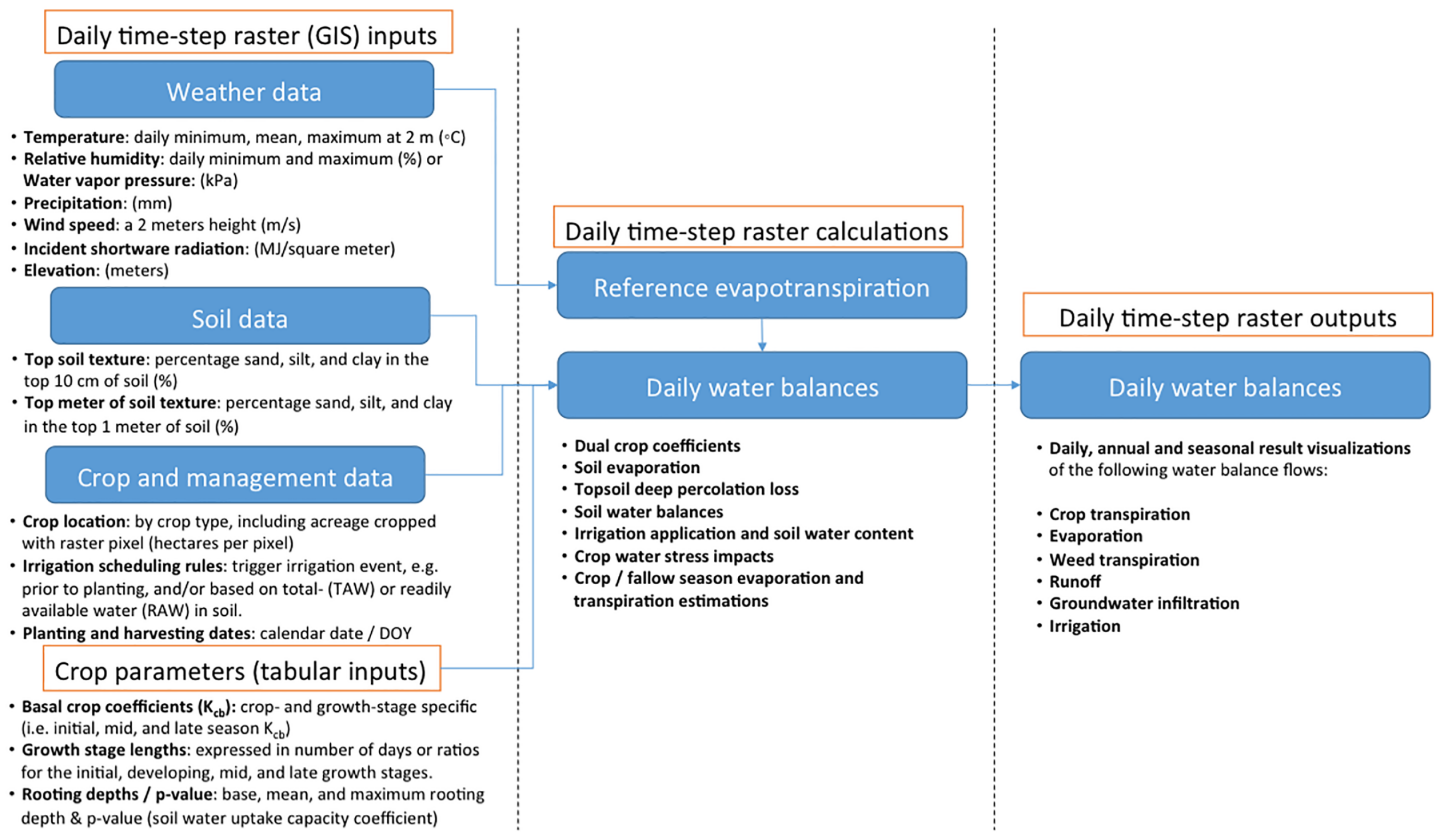
Conceptual flow diagram of CropWatR, focusing on raster file inputs, which need to be provided with daily resolution, and tabular inputs for crop-specific parameters. For further details on the calculation methods, see the supporting information file 2 (S2 File).

On the basis of these inputs, CropWatR can be used to estimate water flows between the soil, crop, and atmosphere under various soil types, water deficits, and crop management practices. The model estimates at a daily time-step growing and fallow season evaporation and crop transpiration, root zone water balances, crop water stress, and irrigation scheduling, the later of which can be calibrated to external surveys of irrigation volumes at an any level of spatial resolution. Fig 1 provides a high-level conceptual flow diagram of the required inputs and crop parameters, as well as the main calculations and outputs. For a more detailed explanation of CropWatR, as well as a complete list of the calculations performed, readers are referred to the second Supporting Information file (S2 File).

Cropping, land use, and irrigation changes simulated by BEPAM at the CRD resolution under each of the three scenarios were downscaled for more detailed biophysical modeling in CropWatR by allocating cropland first to those 10 by 10 km pixels with the highest frequency of cropping for each crop type, as assessed by National Agricultural Statistic Service Cropscape GIS data product over the years 1997–2014 (with complete national coverage beginning in 2004) [62]. The year 2008 is taken as a base year for calibrating CropWatR due to the availability of detailed data regarding cropping practices in that year—state-level irrigation survey data and planting and harvesting dates are taken from the National Agricultural Statistics Service [63, 64]. The annual weather profile is taken as static in modeling subsequent years. As outlined in the S2 File, crop- and vegetation-specific parameters were calibrated to MODIS 16 satellite data estimates of evapotranspiration[65, 66] and then validated against plot and field studies. Further details on downscaling methods, data sources and spatial interpolation algorithms used to derive raster surfaces of daily weather inputs, soil texture, and crop management, the derivation of reference evapotranspiration, and base year calibration and validation are available in S2 File and in Teter [50].

**Fig 2:**
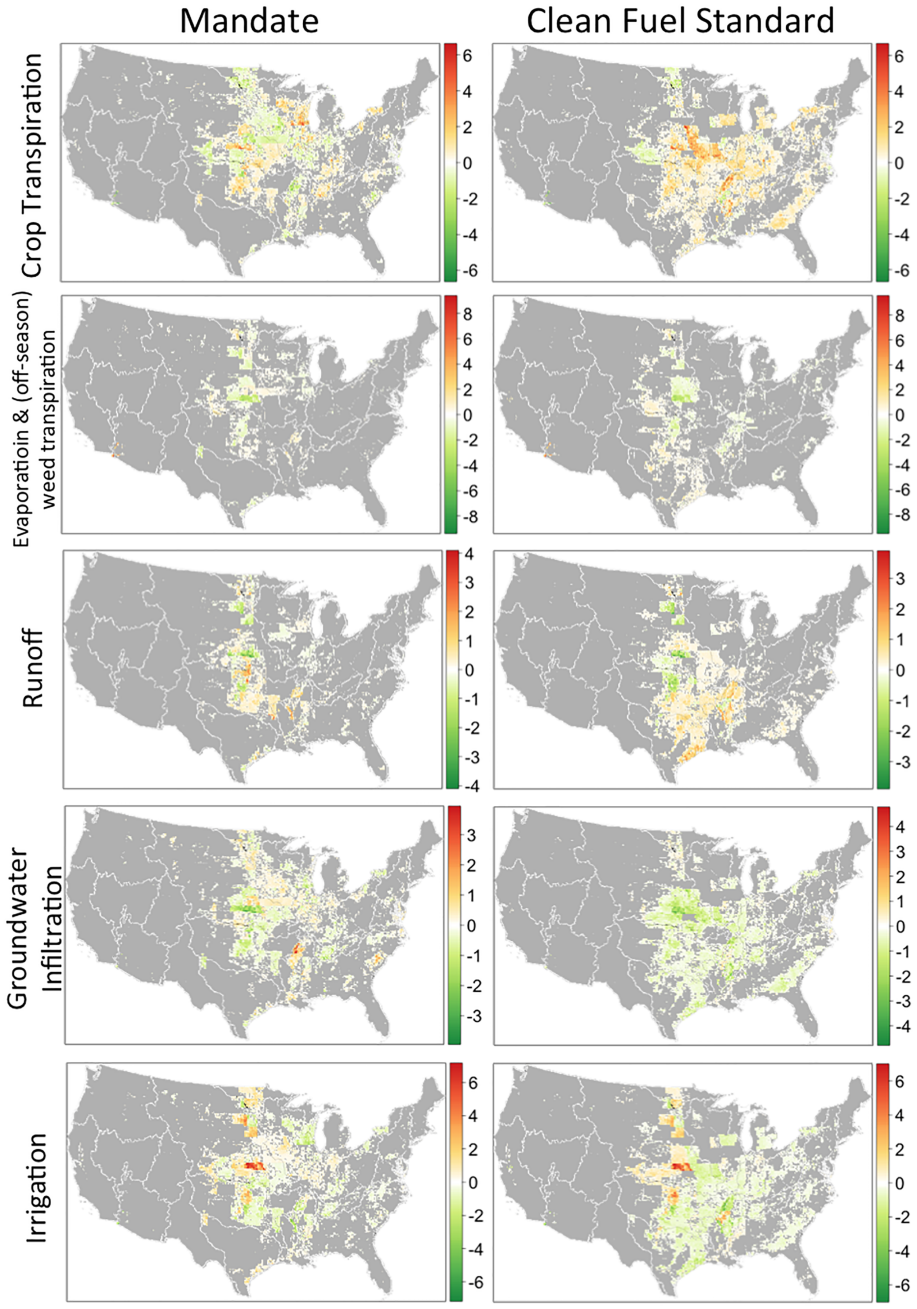
Differences in billion liters in agricultural water balances in the Mandate scenario (left panel) and CFS scenario (right panel) relative to the no-policy counterfactual. Maps show the spatial distribution of changes by water flow.

Model calibration and validation were done based on available site-specific observed data and other reported values in the literature. Crop-specific state-level irrigation volumes were calibrated to roughly match survey reported irrigation intensities per hectare (acre). The results of this calibration are report in Fig 2 and Fig. L in S2 File. Model validation was done against by comparing modelled evapotranspiration against both satellite and survey data. Random samples of MODIS 16 estimates of actual 8-day evapotranspiration were extracted in locations where high shares of the land were cropped in 2008 with row crops, and these time-series were compared with crop specific daily evapotranspiration calculated with CropWatR at the same geographic location (as an illustration, see Figs F-H and M in S2 File). Using the MODIS 16 as a benchmark, common model performance metrics for accuracy (absolute relative error), bias (mean relative error), and reliability (the Nas-Sutcliffe model efficiency coefficient), were performed for all major crop types. These performance metrics are reported in Table C in S2 File. A separate validation was performed by comparing evapotranspiration as modelled in CropWat across various states with state- and location-specific literature estimates (Table D in S2 File). More details on the CropWatR model, its calibration and validation, and on the assumptions used in this study can be found in the S1 File Section III, the separate S2 File that details CropWatR, and in Teter [50].

The BEPAM-CropWatR analysis gives information about differences between the three scenarios concerning regional and national patterns of crop-water use. Absolute and differential water flow rates over given areas (in billion liters) are estimated on an annual basis for the following key water flows: (i) *irrigation* (volumes applied at the crop roots); (ii) the sum of *evaporation* (which is estimated for all land use types) and *off-season transpiration* of weeds/other non-harvested plants (sometimes referred to as non-productive water use), (iii) *transpiration of cultivated crops* (sometimes referred to as productive water use); (iv) *runoff* at the plot level then aggregated across plots, and; (v) *groundwater infiltration*. Irrigation corresponds to blue water (BW) use, and the sum of (growing season) evaporation and transpiration corresponds to the green water (GW) consumption that is associated with crop production. An illustration of these key water flows is shown in Fig H in S1 File. CropWatR enables these water balances to be estimated for each crop and land use type (given in mm water depth, an intensity without a spatial component); and in million liters per 10 by 10 kilometer (about 6.2 by 6.2 miles. 1 acre-foot ≈ 1.23 million liters).

## Results

We estimate the impacts on the balances of five key water flows across the US in the immediately following section and compare the results across scenarios in more detail in the section, “Comparison of Water Use Across Alternative Policy Scenarios”. Spatially explicit water use impacts are examined in greater detail in the section, “Regional and national impacts,” and the results are aggregated to the national level and compared with the literature in the final section of the results, “Water Footprint of biofuels aggregated to the national level”.

### Combined effects of land use change and crop changes on water balances

The patterns associated with Mandate and CFS scenarios can be categorized as one of the following four concerning LUC and related water impacts:

1. **Cropland pasture converted to permanent row crops cultivation**. Transpiration increases in a location are primarily determined by the reference evapotranspiration, the water use intensity of the particular crop, and the length of the growing season. Growing season evaporation and off-season evapotranspiration (ET) both decrease proportional to the differences in weed and bare soil ET relative to uncropped perennial land cover in the BAU scenario. Groundwater infiltration decreases during the growing season, though this decrease may be somewhat offset by an increase over the off-season. Runoff increases in the off-season. In the Southern Plains, irrigation water needs are higher (ranging as high as 10%-25% more across large sections of southeast Nebraska and northeast Kansas, under both the Mandate and CFS Scenarios), leading to increased irrigation for row crops (since it is assumed that energy crops are rainfed). This is the primary LUC type for Mandate scenario.
2. **Land cropped with row crops is displaced by dedicated energy crops**. Due to the higher growth rates (i.e., high transpiration rates) and longer growing season of energy crops compared with most raw crops, this LUC category leads to an increase in transpiration (typically on the order of 15% to 30%, but in some cases as high as 60% to 80%, depending on the row crop replaced by switchgrass or miscanthus and on climate conditions), and to decreases in all other water flows, including irrigation. This occurs primarily in the CFS scenario, wherein about 4% of area cropped in row crops is displaced by energy crops.
3. **Idle cropland and cropland pasture is displaced by energy crops**. Effects are similar to those in category (2), but the total annual increases in transpiration and decreases in groundwater infiltration may be more muted (although growing season transpiration increases substantially). Moreover, runoff in the post-harvest period may increase somewhat (generally by no more than 20%). Although the majority of energy crops are planted on marginal lands (77% of total area) in the Mandate scenario, the total area of marginal lands cropped by energy crops is more than four times greater in the CFS scenario (1.69 million hectares).
4. **Land cropped with row crops is taken out of production**. This category of LUC is atypical (accounting for only 2% and 6% of the total cropped land in Mandate and CFS, respectively), but does occur. The water use impacts are opposite to those of category 1, and irrigation requirements are reduced.

### Comparison of water use across alternative policy scenarios

The land use and total water balances across all modeled crops in the base year (Fig I in S1 File) serve as a useful basis of comparison against the water balances modeled in Mandate and CFS scenarios in the final model year (2030). Fig 2 shows the differences in annual water flows across the contiguous United States in Mandate and CFS scenarios, compared with the BAU scenario. The magnitude of water balance change on the scale of a single grid cell (10 x 10 km) is small (typically less than ±10%) in most grid cells, as shown in the density plots (Fig J in S1 File). The maps in Fig 2 show those localities with more pronounced (ranging from ±10% to as high as ±50% in isolated regions, and even greater changes in the case of adoption or cessation of irrigation) decreases (green) and increases (red) in a few grid cells. These are regions where more pronounced impacts of biofuel policies on water resource availability and hydrologic processes are more likely to occur. The broad white areas correspond to regions with a clear pattern (i.e., increased or decreased water flows) but very low intensity (with changes to water balances of less than 3.5% on average), i.e. areal extent is broad, but impact per hectare is small. The grey areas correspond to regions of the country where water balances are essentially unchanged (i.e. where changes are less than the first percentile of average grid-level water balance changes).

Of these four categories discussed in Section 3.1, the most pronounced water use impacts (with changes in water flows on the order of ±10% to ±50%) are of type (1), i.e. in those regions where cropland pasture is converted to row crops. Corresponding to LUC categories (2) and (3), the signal of increased transpiration counterbalanced by decreased groundwater infiltration and irrigation across the eastern states is also evident in CFS scenario. Results can be usefully described at the broadest level of hydrologic classification used by the United States Geological Survey, by Water Resource Regions (WRRs), which divide the contiguous United States into 18 regions, following drainage areas of major rivers. Within any given WRR, water flow changes will be positive for some grid cells, and negative for others. The positive and negative water flow changes may counterbalance one another at the WRR level; for instance, in Ohio WRR under Mandate, positive and negative changes in crop-water flows approximately counterbalance each other, muting the net signal that a shift occurs from evapotranspiration to economically productive transpiration. In other instances, a clear increase in a given water flow is evident; for instance, under Mandate, irrigation requirements increase substantially over much of the Missouri WRR.

Table 2 summarizes the net nationwide differences in annual water flows in Mandate and CFS, respectively, compared to the BAU. The general trends are similar in both scenarios, although the increase in transpiration and commensurate decrease in groundwater infiltration, as well as the increase in runoff, is greater in CFS. Even though overall effects are relatively small, the effects differ for different sources of water and different regions. Net irrigation requirements increase 0.7% in Mandate and decrease 3.8% in CFS, but in both scenarios increases are concentrated in regions of Kansas and Nebraska that rely upon the Ogallala aquifer for irrigation water.

**Table 2:** Net and percentage water flow changes relative to the no-policy counterfactual (billion liters).

Annual water balance	BAU	Mandate	CFS
	(Percent change from BAU)		
Productive (Food / feedstock) transpiration	163900	545 (0.3%)	9103 (5.6%)
Unproductive (fallow season) transpiration and evaporation	85400	-970 (-1.1%)	-886 (-1.0%)
Runoff	69200	282 (0.4%)	1678 (2.4%)
Groundwater infiltration	113700	-587 (-0.5%)	-5797 (-5.1%)
Irrigation	109100	729 (0.7%)	-4070 (-3.8%)

Note that the crops modeled in BEPAM represented about 67% of irrigation water applications in 2008 and 2013, with remaining irrigation water applications to orchards, vegetables, pasture land, and other crops not modeled in BEPAM.

### Regional and national impacts

**Fig 3:**
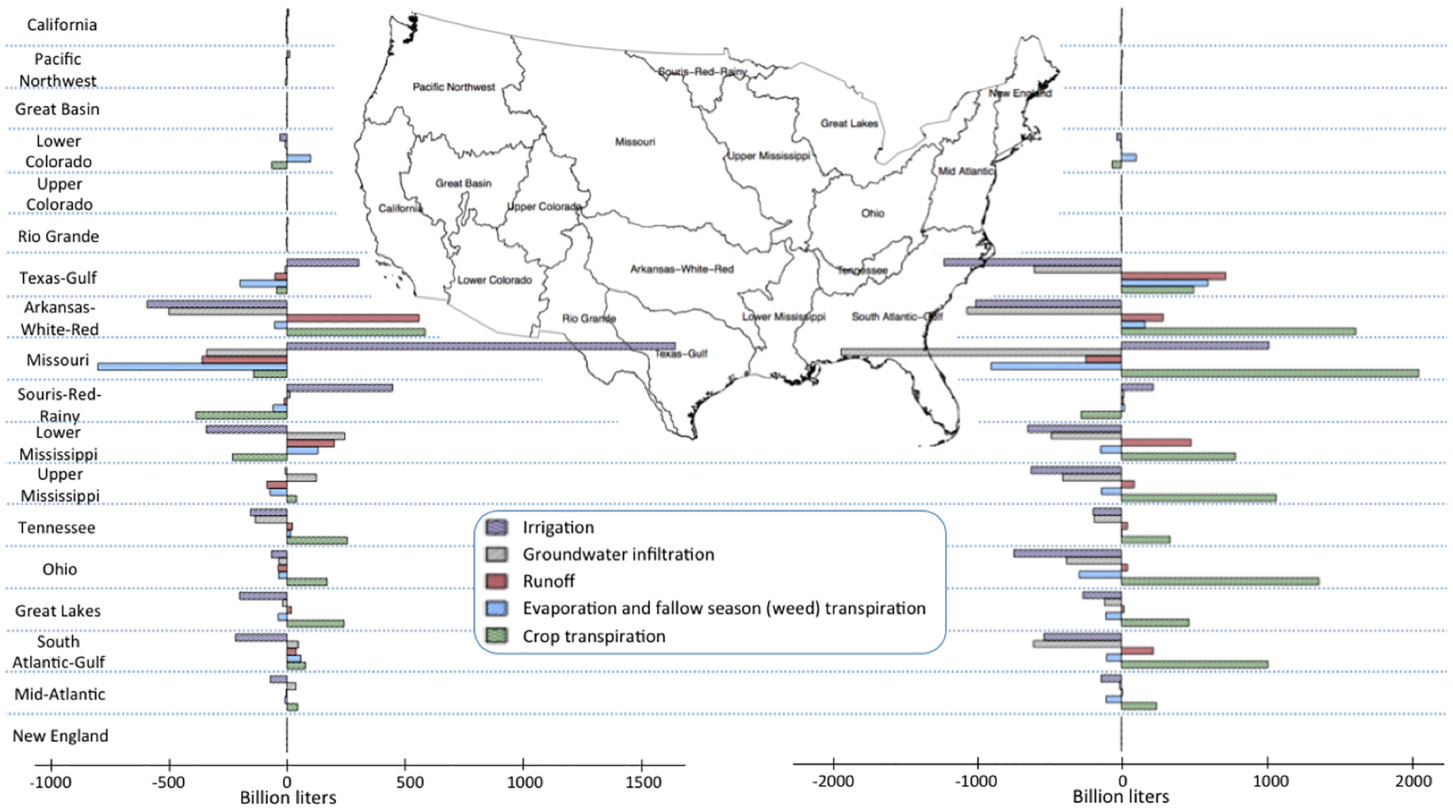
Net water balance changes (in billion liters) in the Mandate scenario (left) and in the CFS scenario (right) by water resource region (WRR).

Fig 3 shows net water flow changes summed separately within each of the WRRs in Mandate and CFS scenarios, respectively, relative to the BAU. In CFS, diminished irrigation requirements throughout most of the eastern U.S. result from the displacement of row crops by bioenergy crops. Increased irrigation requirements in the Missouri WRR occur due to partial displacement of these same row crops to Nebraska, Kansas, and other states. Results also suggest reduced irrigation (though of smaller magnitudes compared with CFS) across the Eastern U.S. in Mandate, but with significant increases in Missouri and other WRRs west of the Mississippi river. As can be seen from Fig 3, the general pattern of changes in crop water use are: (1) an increase in transpiration nationwide and in most regions, particularly under CFS scenario, and a commensurate decrease in evaporation and post-harvesting season transpiration; (2) slightly decreased irrigation water use east of the Mississippi—which is a result of displacement and substitution of row crops by energy crops, (3) regions of substantial increase in irrigation water requirements in the Great Plains from Texas to Nebraska, and (4) runoff increases in regions under the most extreme extensification—in Kansas and Oklahoma under Mandate and in Texas and the lower Mississippi under CFS.

A net increase in transpiration (0.3% vs. 5.6% in Mandate and CFS respectively) and a commensurate net decrease in groundwater infiltration (-0.5% vs. -5.1% in Mandate and CFS respectively) and annual evaporation and off-season transpiration (~1% in both scenarios) are observed in both Mandate and CFS. The regions where transpiration increases most are those that have the largest increases in cropped area–Wisconsin, Illinois, North and South Dakota, Oklahoma, Kansas, Nebraska, and east of the mid stretches of the Mississippi in Mandate, and stretches along much of the eastern US, but most extreme in the Great Plains, from Texas north to North Dakota, and along the mid-stretches of the Mississippi, in CFS. These pronounced increases in cropped area under CFS are a combination of direct cultivation of energy crops on prime cropland and of crop displacement—as prime cropland in eastern states is converted to energy crops, row crops are instead grown in the states listed above (as well as in the Midwest and Corn Belt).

Net decreases in national irrigated acreage do not necessarily translate to decreases in national net irrigation water use as the irrigation intensity of cultivation varies regionally and from crop to crop. Specifically, increased cultivation of corn in regions where substantial irrigation is required (Oklahoma, Kansas, and Nebraska) leads to increased net irrigation in Mandate, while cultivation of rainfed (by assumption) energy crops actually substitutes for crops that require very little irrigation across the eastern US. The regions where irrigation water use increases under both scenarios are in the Midwest, South, and Great Plains: mostly in Texas, Oklahoma, Kansas, Nebraska, and North and South Dakota. In the Mandate scenario, net increases in irrigation water requirements occur in regions of Texas, Oklahoma, and Kansas that source the vast majority (>90%) of their irrigation from the Ogallala Aquifer, a resource from which water is currently being drawn at unsustainable rates [67, 68]. Smaller increases (~l billion liters a year) in irrigation water requirement in Missouri WRR in observed in CFS.

### Water footprint of biofuels aggregated to the national level

**Fig 4:**
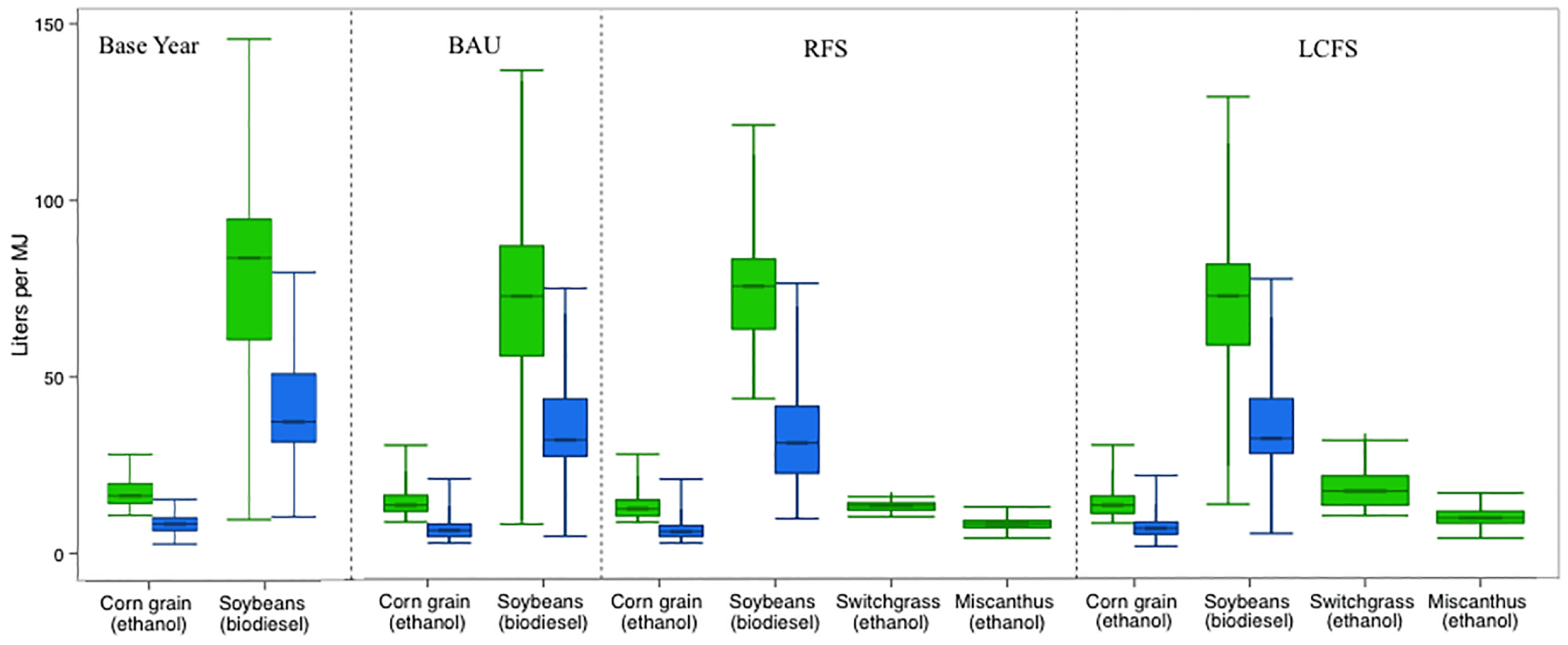
Green (soil water and rainfall) and blue (irrigation) water footprint of feedstock cultivation in the base year, no-policy counterfactual (BAU), Mandate, and CFS scenarios. Box plots show the area-weighted 5^th^, 25^th^, mean, 75^th^ and 95^th^ percentiles.

Spatially explicit blue and green WFs (measured in L water/MJ biofuel) for biofuel feedstocks are derived under the assumption of a uniform distribution of corn and soybean for biofuel production (i.e. no specific region contributes greater proportion of feedstock to biofuel production). Feedstock and pathway-specific conversion efficiencies are taken from literature [11]. The aggregated WFs for biofuel feedstocks are shown in Fig 4 for the three scenarios. Fig 4 shows the green- and blue WFs of corn and soybean in the base year (2008), and in each of the scenario for these two main agricultural feedstocks plus Miscanthus and switchgrass. Variability in WF is primarily a function of geography. The lower WFs of corn grain ethanol and soybean-based biodiesel in BAU compared to the base year is due to yield increases assumed in BEPAM, which lead by the final year (2030) to increased productivity per unit area of these crops by 29% and 12%, WFs of corn grain ethanol and soybean-based biodiesel in 2030 in the BAU is smaller compared to the base year (2008). The national area-weighted average green and blue WFs for corn cultivation in the base year were 18.3 L/MJ of ethanol (with a 5%-95% range from 12.0–49.2 L/MJ) and 10.2 L/MJ (5%-95% range from 5.0–30.9 L/MJ), respectively. For soybean biodiesel, the national area-weighted average green and blue WFs were 85.3 L/MJ and 34.3 L/MJ, respectively.

## Discussion

The modeling framework adopted represents an attempt to quantify the water use and availability impacts of different biofuels policies, both of which achieve the same GHG reductions. Broadly speaking, a Clean Fuel Standard (CFS)-type policy would require less irrigation water nationally and less pronounced impacts on irrigation water requirements in key regions than the Mandate. This result is predicated, however, on the assumption that cellulosic energy crops would be rainfed. At the same time, the expansion of cellulosic energy crop production on marginal (formerly idle and pasture land) under the CFS may result in more runoff and reduced groundwater infiltration.

The study also pinpoints regions where water balance impacts are very pronounced (i.e. greater than ±25%) for more detailed hydrologic assessment. Comparison of water use impacts under varying biofuel targets and policy designs, as well as using CropWatR, which is available as open source software on Github, to assess regional impacts as modeled by economic, land use, and policy models other than BEPAM, can improve the robustness of this approach. By confirming or refuting specific results, such an approach can either provide greater confidence in the results or alternately illuminate key uncertainties. This approach can be replicated to assess the water resource use impacts of other biofuel policy alternatives, as well as different modeling and economic assumptions. Crucially, using CropWatR to model differences in water balances based on the land use outputs of various models and/or policy scenarios allows estimation of the water impacts of direct and indirect land use change.

The key results and insights obtained from the analysis are:

1. Both Mandate and CFS shift irrigation patterns to the Western Corn Belt and the Southern Great Plains. Cropped land area increases by 2% and 6% under Mandate and CFS scenarios, respectively. Though there is a net decrease in the acreage of irrigated cropland of 2% in Mandate and 0.3% in CFS scenario, net irrigation water increases by 0.7% in Mandate scenario, and decreases by 3.8% under CFS, compared to BAU. Both Mandate and CFS result in lower irrigation in certain regions by incentivizing rainfed feedstock cultivation that displaces irrigated row crops, and they lead to more cultivated area in arid Great Plains (including Kansas, Nebraska, Oklahoma, the panhandle of Texas, and North and South Dakota) due to an overall increase in corn acreage under Mandate and displacement of cropping of corn, soy, and other crops under CFS.
2. By increasing total cropped land area, Mandate and CFS also lead to a net increase in ET with concomitant reductions in groundwater infiltration recharging surface and potentially also groundwater stocks over agricultural lands throughout the West, Midwest, Corn Belt, and the rest of the Eastern United States. Increases in ET are more pronounced in the CFS scenario, which are mostly offset by greater reductions in groundwater infiltration than in Mandate (see Table 2).
3. Increased runoff in regions where formerly uncropped land is converted to row crop and energy crop cultivation, for instance along states in the Lower Mississippi WRR. Thus, extensive cropping of corn and possibly energy crops may also lead to greater runoff in regions of the Ohio and Upper and Lower Mississippi WRRs. While the magnitude of this effect is similar in the CFS and Mandate scenarios in some regions, it is more pronounced in the Mandate scenario in northeastern Kansas and Southwestern Nebraska (as a result of increased corn planting), whereas the spatial extent of runoff increases in the CFS is far wider, particularly in the Lower Mississippi WRR.

The results of our study are consistent with past observations of corn expansion into the frontier states (i.e. the six states north from North Dakota, south to Texas) due to biofuel policies [4, 10, 69]. Our results are also consistent with earlier studies suggesting that miscanthus and switchgrass utilize more water than corn [43, 49, 70] and may thereby reduce stream flows, though one recent study suggests measured water use by perennial systems was similar to corn across normal and drought years and suggests that rain-fed perennial biomass crops in US Midwest have little impact on landscape water balances [71].

The modeled blue and green water footprints for biofuel feedstocks shown in Fig 4 were compared with point estimates and ranges reported in the literature [14, 15, 21, 26, 47, 70, 72–74]. These estimates vary widely and besides regional variation, differences can be attributed to input data, year of consideration, allocation methods, and different metrics for estimating water consumption. Comparisons with other studies are summarized in the Supporting Information 1 (S1 File) Section IV in Table D in S1 File. Dominguez-Faus, Powers [21] estimate 60 L/MJ for US average corn ethanol and 198 L/MJ for US average soybean biodiesel. For miscanthus and switchgrass, our estimates are similar to Zhuang, Qin [49] but much lower than Song, Cervarich [75]. One major difference between our study and other studies is the use of economic optimization model that favors production areas and agricultural practices with higher yields.

Most studies comparing corn and soybean with perennial energy crops suggest that the latter are associated with lower runoff [46, 70, 76], which is consistent with the findings of this study when considering direct substitution between corn or soy and switchgrass and miscanthus. However, this study finds that conversion of idle cropland or cropland pasture to cellulosic energy crops could increase runoff over large areas. One recent study looking at the displacement of grassland with energy crops found reduction in surface runoff, where the reductions were greater under displacement by Miscanthus compared to switchgrass [76]. This study found that surface runoff from pasture areas was high in the months of June to August, compared to perennial energy crops due to summer grazing of pasture in the study area. Our study does not consider grazing, and shows higher runoff during the post-harvest period. It has been widely recognized that cropland pasture is a broad category and there is a huge ambiguity in its definition, as it encompasses many vegetation types, management practices and transition phases [3, 77]. This heterogeneity in vegetation and management may have a substantial impact on the parameterization of water use for this type of land [78]. This is certainly one of the largest areas of uncertainty in this study, and clarifying the geographic variation in cropland pasture water balances as a baseline for comparison will require future studies to carefully consider types of cropland pasture and their conditions in each region.

Some care is warranted in interpreting and extrapolating from this study. First, the BEPAM model used as the basis for deriving water balances and differentials in water flows among scenarios makes certain assumptions, where important ones are (1) that annual yields of row crops increases following a historical trend, and (2) that cultivation of switchgrass and miscanthus is rainfed and their yields levels are fixed over the entire time period. The results obtained here are values under average conditions, i.e. no annual yield variability due to weather variations are included for any crop. The lack of accounting for changing climatic conditions in both historical and future years is a key limitation of the modeling, and the sensitivity of model results to historical weather patterns and projected future climate inputs would be a worthwhile object of future exploration. No sensitivity analysis was done to explore the impacts of different modeling assumptions (e.g. oil price trends or supply and demand price elasticity values in the BEPAM economic modeling, or weather variability or future changes due to climate change in the biophysical inputs to CropWatR) on water balance results.

Another caveat is that there is no consideration of wider scale effects (e.g., at the plot to hydrologic basin scale) on water quality and surface- and groundwater recharge. Crop-water balances are modeled here only on the plot level; since larger scale hydrologic impacts are heavily dependent on spatially heterogeneous hydrologic properties are larger scale (e.g. at the catchment, drainage basin, and watershed levels), this study’s main utility is to identify ‘hotspots’ of greatest potential impact for more detailed hydrologic modeling. Translating direct crop-water balances into landscape and basin level scale hydrologic flows requires interfacing crop-water interactions into a hydrologic model. Such detailed hydrologic studies—using for instance SWAT [79] or EPIC [80]–are useful for post-hoc assessment of LUC and their hydrologic impacts [46, 81, 82] or for scenario assessments with regionally or locally defined scope [82–85], but they are less easy to implement for studies attempting to characterize water use impacts over a wide geographic area, as they require extensive data that are not always readily available for parameterization, and necessitate extensive regional calibration and validation[82, 86].

Unlike WU-LCA and WF studies that often include a scarcity assessment based on the ISO 14046 standard [20, 87], our study has not considered the impacts of changes of water flow balances. This will be an important future work to understand the impacts at local/regional level and to involve policy makers in finding solutions that minimize or mitigate such impacts. Nor do we consider water quality effects of biofuels policies as data are unavailable at the model resolution, and are of variable quality across the contiguous US. To the extent that policies increase or decrease acreage cropped in corn, soybeans, and other crops requiring high nitrogen (and phosphorus) fertilizer input, they may either exacerbate or mitigate nutrient loading to surface waters. Similarly, changes in tillage practices or displacement of crops by varieties with growing seasons of different lengths imply shorter or longer fallow periods. If the length of the off-season is increased by bringing formerly uncropped land into cultivation or through substitution to crops with a shorter growing season, runoff volumes and nutrient discharge may increase. A few regional studies include an analysis of water quality impacts of recent cropping trends, which are then attributed to the amended Renewable Fuel Standard, the RFS2 [46, 81], or cited as potential future consequences of biofuels policies [84, 86, 88]. On the other hand, studies generally show positive impacts, in terms of higher water use efficiency (e.g., liters biofuel per m^3^ water)[49] and reductions in nutrient and sediment concentrations in streams [89, 90] switching from food crops to energy crops. One shows reductions in stream flow and surface runoff for both corn/soybean and pasture changing to energy crops [91] though more studies are needed to reach robust conclusions across the range of vegetation and management types categorized as grassland.

## Supporting information

S1 File Supporting Information for ‘Water impacts of U.S. biofuels: Insights from an assessment combining economic and biophysical models’. **Table A. Crop and land use categories modeled in BEPAM, CDL, and CropWatR. Table B. Area cropped in million hectares in the base year (2008) and at the end of the modeling period by scenario.** Delta values show the percent change in the policy scenario compared with the BAU. **Table C. Million hectares irrigated at the end of the modeling period in each scenario.** Nationwide changes in irrigated area, by crop, in million hectares. Deltas are the percent difference between the Mandate and CFS scenarios from the counterfactual (no-policy BAU) at the end of the modeling period, respectively. **Table D. Literature estimates of blue and green water use for cultivation of biofuels feedstock. Fig A. Base Year (2008) cropping patterns.** Colors indicate the percent of land cropped in each 10 x 10 kilometer pixel. **Fig B. BAU cropping patterns at the end of the modeling period, in 2030. Fig C. Mandate scenario cropping patterns at the end of the modeling period, in 2030. Fig D. CFS scenario cropping patterns at the end of the modeling period, in 2030. Fig E. Land converted for cellulosic feedstocks in the Mandate scenario.** Area cultivated in miscanthus and switchgrass as a percentage of total regular cropland and marginal land, at the resolution of 10 x 10 kilometers at the end of the modeling period. Total land cropped in miscanthus is the sum of land cropped in regular cropland and in marginal land. Switchgrass is cropped only on regular cropland in the Mandate scenario. **Fig F. Land converted for cellulosic feedstocks in the CFS scenario.** Area cultivated in miscanthus and switchgrass as a percentage of total land, at the resolution of 10 x 10 kilometers at the end of the modeling period. Total land cropped in both miscanthus and switchgrass is the sum of land cropped in regular cropland and in marginal land. **Fig G. Land use change (increase or decrease in cropland, at 10 x 10 km resolution) in the Mandate (top) and CFS (bottom) scenarios, relative to the no-policy counterfactual (BAU) at the end of the modeling period.** Net changes decomposed by latitude and longitude, respectively, are shown above and on the right. **Fig H. Illustration of key water flows modelled in *CropWatR* model. Fig I. Water balances and land use in the base year (2008).** Water balance maps (top five maps) show the annual water flows in ***billion*** liters. The net water balances are aggregated to Water Resource Regions (WRRs) and reported in the bar chart to give annual water flows in trillion liters. The map at the bottom right shows the percentage of cropped land for which water balances were estimated. **Fig J. Differences in billion liters in agricultural water balances across all 10 km x 10 km grid cells, in the Mandate scenario (left panel) and CFS scenario (right panel) relative to the no-policy counterfactual in the final model year.** The density plots of the changes including the *median* (red vertical bar) of all grid cells are shown on the left. **Fig K. Water use intensities (liters/MJ) of corn cultivation in base year (left) and end of modeling year (2030), in the BAU, Mandate, and CFS Scenarios (left to right).** Density plots (right of maps) show the distribution of water use intensities at the pixel scale (10 x 10 km) across all land planted in corn. **Fig L. Water use intensities (liters/MJ) of soybean cultivation in base year (left) and end of modeling year (2030), in the BAU, Mandate, and CFS Scenarios (left to right).** Density plots (right of maps) show the distribution of water use intensities at the pixel scale (10 x 10 km) across all land planted in soybean. **Fig M. Water use intensities (liters/MJ) of miscanthus cultivation at the end of modeling year (2030) in the Mandate (left) and CFS (right) scenarios.** Density plots (right of maps) show the distribution of water use intensities at the pixel scale (10 x 10 km) across all land planted in miscanthus. **Fig N. Water use intensities (liters/MJ) of switchgrass cultivation at the end of modeling year (2030) in the Mandate (left) and CFS (right) scenarios.** Density plots (right of maps) show the distribution of water use intensities at the pixel scale (10 x 10 km) across all land planted in corn.
(ZIP)

S2 File A description of CropWatR. **Table A. K**_**cb**_ **parameter values for perennial grassland and non-crop agricultural land cover types. Table B. Crop water balances that can be estimated for daily, seasonal, or annual time steps. Table C. Literature estimates of evapotranspiration versus modeled evapotranspiration rates.** Seasonal and annual evapotranspiration ranges reported in literature sources and model by *CropWatR*. A range of methods are available for determining evapotranspiration at the field, landscape, and watershed scales, either via direct measurement (e.g. soil moisture measurement via lysimeters), modeling (e.g. via process-based s imulation models, or energy balances using weather data collected via satellite, local instruments, and/or remote sensing). For a summary of common methods, see Connor et al.,2011. **Table C. Model performance metrics comparing the results with MODIS 16 estimates. Table E. NASS Classification categories considered in this analysis.** Note that double-cropped classifications where both crops were not included among the 14 parameterized crops (e.g. lettuce / upland cotton, lettuce / barley, etc.) were excluded from the analysis. The total acreage of these classifications on the national scale was in all cases much less than 1% of the acreage of the modeled crop. **Table F. NASS Accuracy assessments for crops and land types considered in this analysis.** Accuracy statistics for crop and land use categories not reported in the above table are not available for 2008. These categories are: alfalfa, other hay, sugarcane, fallow/idle cropland, grassland herbaceous, and pasture/hay. **Fig A. Relation between time (day since emergence), crop coefficient (Kcb) and plant height for maize.** Crop coefficients are specified for three moments in time. According to the FAO 56 methods, coefficients are estimated by stepwise and linear interpolation (black line). In CropWatR, a Bezier curve (red curve) is used to interpolate daily Kcb values. **Fig B. Irrigation calibration results for corn.** The density plot on the left shows the distribution of state-level mean irrigation intensity in acre-feet per acre, according to the state survey (blue) and as calibrated in the model (red). The map shows the survey reported (blue) and modeled mean (red) irrigation intensities in each state. The colors of the map show irrigation intensity at the 10-kilometer pixel resolution. **Fig C. Annual water requirements for growing maize per pixel thousand acre-feet (total—left) and in millimeters (right)**. Maps in the right-hand column show annual water flows used to grow corn in 2008 in millimeters. Maps in the left-hand column show the water budget volumes scaled by the number of acres grown in corn in each pixel in thousand acre-feet, resulting in the total annual water requirements per 10-kilometer grid cell. **Fig D. Total annual water requirements for corn by water resource region (HUC 2), in million acre-feet.** Water requirements in each region computed based on the acres of corn grown in each state, as shown in the map above the bar chart. **Fig E. Distribution of annual water use for corn by water resource region (HUC 2), in millimeters.** The violin plot shows the continuous distribution of values for each water balance across all pixels modeled in each water resource region, and hence provides a more detailed version of the same information conveyed by a box plot. **Fig F. Validation locations for corn.** Locations selected for validation of corn shown in black. **Fig G**. **Comparisons of modeled daily Evapotranspiration (ET) versus 8-day average ET estimated by MODIS 16.** Daily average evapotranspiration (in mm/day) on each day of the year. Red lines show (smoothed) daily actual ET as estimated by the model, and blue steps show the average ET in each 8-day time step as estimated by the MODIS 16 algorithm. Vertical green lines delineate the start and the end of the growing season (i.e. planting and harvesting dates). Representative results for each crop are shown. More complete results are given in the appendix (Fig M). **Fig H. Scatterplots comparing of modeled annual and eight-day average daily Evapotranspiration (ET) as estimated by *CropWatR* and MODIS 16**. Scatterplots on the left-hand side graph the correlation between annual ET at across all validation sites, those on the right-hand side show correlations across data points (i.e. the eight-day average ET as estimated by MODIS 16 versus the eight-day averaged modeled ET according to *CropWatR*). The diagonal lines bisect the graph (y = x). **Fig I. Annual summary maps of daily input parameters for calculating reference evapotranspiration. Fig J. Derived aggregated coverage of crops and other land covers modeled in 2008, from the NASS CDL.** Crops shown in order of decreasing acreage. **Fig K. Soil classification for soils of at least one meter depth. Fig L. Irrigation Calibration Results.** The density plots show the distribution of state-level mean irrigation intensity in acre-feet per acre, according to the state survey (blue) and as calibrated in the model (red). The maps show the survey reported (blue) and modeled mean (red) irrigation intensities in each state. The colors of the map show irrigation intensity at the 10 × 10 kilometer pixel resolution. **Fig M. Daily Modeled ET and MODIS 16 8-day Average ET Estimates.** Representative results of the validation at two locations for each crop. **Fig N. Evapotranspiration Validation Locations.** The maps show the density of cropping and the locations in each of the 18 Water Resource Regions (HUC2 regions) sampled to validate modeled daily actual ET against 8-day average ET as modeled by MODIS16. Locations were chosen where the density of the given cropping / land use classification was most dense in each of the HUC2 regions.
(ZIP)
