# Supplementary material for: Water impacts of U.S. biofuels: Insights from an assessment combining economic and biophysical models
Source: PLoS One. 2018 Sep 28;13(9):e0204298. doi: 10.1371/journal.pone.0204298 (PMC6161887; doi:10.1371/journal.pone.0204298)
Supplement: S1 File — Table A. Crop and land use categories modeled in BEPAM, CDL, and CropWatR. Table B. Area cropped in million hectares in the base year (2008) and at the end of the modeling period by scenario. Delta values show the percent change in the policy scenario compared with the BAU. Table C. Million hectares irrigated at the end of the modeling period in each scenario. Nationwide changes in irrigated area, by crop, in million hectares. Deltas are the percent difference between the Mandate and CFS scenarios from the counterfactual (no-policy BAU) at the end of the modeling period, respectively. Table D. Literature estimates of blue and green water use for cultivation of biofuels feedstock. Fig A. Base Year (2008) cropping patterns. Colors indicate the percent of land cropped in each 10 x 10 kilometer pixel. Fig B. BAU cropping patterns at the end of the modeling period, in 2030. Fig C. Mandate scenario cropping patterns at the end of the modeling period, in 2030. Fig D. CFS scenario cropping patterns at the end of the modeling period, in 2030. Fig E. Land converted for cellulosic feedstocks in the Mandate scenario. Area cultivated in miscanthus and switchgrass as a percentage of total regular cropland and marginal land, at the resolution of 10 x 10 kilometers at the end of the modeling period. Total land cropped in miscanthus is the sum of land cropped in regular cropland and in marginal land. Switchgrass is cropped only on regular cropland in the Mandate scenario. Fig F. Land converted for cellulosic feedstocks in the CFS scenario. Area cultivated in miscanthus and switchgrass as a percentage of total land, at the resolution of 10 x 10 kilometers at the end of the modeling period. Total land cropped in both miscanthus and switchgrass is the sum of land cropped in regular cropland and in marginal land. Fig G. Land use change (increase or decrease in cropland, at 10 x 10 km resolution) in the Mandate (top) and CFS (bottom) scenarios, relative to the no-policy counterfactual (B [file pone.0204298.s001.zip › SI 1 Figures Tables/S1 Table B.docx]

**S1 Table B. Area cropped in million hectares in the base year (2008) and at the end of the modeling period by scenario.**

| Crop | 2008  (Base Year) | End of modeling period (2030) | | | | |
| --- | --- | --- | --- | --- | --- | --- |
|  |  | BAU | Mandate | Δ | CFS | Δ |
| Winter wheat | 14.20 | 18.25 | 17.56 | -4% | 15.78 | -13% |
| Alfalfa & other hays | 23.76 | 22.99 | 22.66 | -1% | 21.85 | -5% |
| Corn | 32.58 | 28.85 | 36.22 | 26% | 30.51 | 6% |
| Soybeans | 29.06 | 29.54 | 25.37 | -14% | 27.15 | -8% |
| Switchgrass |  |  | 0.02 |  | 1.54 |  |
| *regular cropland* |  |  | 0.02 |  | 0.43 |  |
| *cropland pasture* |  |  |  |  | 1.10 |  |
| Miscanthus |  |  | 1.94 |  | 10.72 |  |
| *regular cropland* |  |  | 0.43 |  | 5.50 |  |
| *cropland pasture* |  |  | 1.51 |  | 5.22 |  |
| Other Crops | 15.09 | 19.87 | 18.33 | -8% | 18.66 | -6% |
| Total Acreage | 114.73 | 119.46 | 122.13 | 2% | 126.18 | 6% |

Delta values show the percent change in the policy scenario compared with the BAU.
