# Supplementary material for: Water impacts of U.S. biofuels: Insights from an assessment combining economic and biophysical models
Source: PLoS One. 2018 Sep 28;13(9):e0204298. doi: 10.1371/journal.pone.0204298 (PMC6161887; doi:10.1371/journal.pone.0204298)
Supplement: S1 File — Table A. Crop and land use categories modeled in BEPAM, CDL, and CropWatR. Table B. Area cropped in million hectares in the base year (2008) and at the end of the modeling period by scenario. Delta values show the percent change in the policy scenario compared with the BAU. Table C. Million hectares irrigated at the end of the modeling period in each scenario. Nationwide changes in irrigated area, by crop, in million hectares. Deltas are the percent difference between the Mandate and CFS scenarios from the counterfactual (no-policy BAU) at the end of the modeling period, respectively. Table D. Literature estimates of blue and green water use for cultivation of biofuels feedstock. Fig A. Base Year (2008) cropping patterns. Colors indicate the percent of land cropped in each 10 x 10 kilometer pixel. Fig B. BAU cropping patterns at the end of the modeling period, in 2030. Fig C. Mandate scenario cropping patterns at the end of the modeling period, in 2030. Fig D. CFS scenario cropping patterns at the end of the modeling period, in 2030. Fig E. Land converted for cellulosic feedstocks in the Mandate scenario. Area cultivated in miscanthus and switchgrass as a percentage of total regular cropland and marginal land, at the resolution of 10 x 10 kilometers at the end of the modeling period. Total land cropped in miscanthus is the sum of land cropped in regular cropland and in marginal land. Switchgrass is cropped only on regular cropland in the Mandate scenario. Fig F. Land converted for cellulosic feedstocks in the CFS scenario. Area cultivated in miscanthus and switchgrass as a percentage of total land, at the resolution of 10 x 10 kilometers at the end of the modeling period. Total land cropped in both miscanthus and switchgrass is the sum of land cropped in regular cropland and in marginal land. Fig G. Land use change (increase or decrease in cropland, at 10 x 10 km resolution) in the Mandate (top) and CFS (bottom) scenarios, relative to the no-policy counterfactual (B [file pone.0204298.s001.zip › SI 1 Figures Tables/Supporting Information 1.docx]

**Supporting Information for ‘Water impacts of U.S. biofuels: insights from an assessment combining economic and biophysical models’**

Jacob Teter^1*^, Sonia Yeh^2^, Madhu Khanna^3, 4^, Göran Berndes^2^

^1^ International Energy Agency, Sustainable Technology Outlooks, Paris, France

^2^ Department of Space, Earth and Environment, Chalmers University of Technology, Gothenburg, Sweden

^3^ Department of Agricultural and Consumer Economics, University of Illinois at Urbana Champaign, Urbana, IL, United States of America;

^4^ DOE Center for Advanced Bioenergy and Bioproducts Innovation, University of Illinois at Urbana-Champaign, Urbana IL, United States of America

* Corresponding author

E-mail: [Jacob.TETER@iea.org](mailto:Jacob.TETER@iea.org) (JT)

The supporting information is organized into the following sections:

1. More detailed assumptions
   1. Land use allocation of BEPAM, CDL, and CropWatR
   2. Land use and land use change change under the BAU and policy scenarios
2. More detailed results:
   1. Water balances
   2. Geographic distribution of water use intensity, by feedstock and scenario
3. The CropWatR model
4. Literature estimates of blue and green water use for biofuel feedstock cultivation

#### I. More Detailed Assumptions about Land Use

#### I.a. Land use allocation of BEPAM, CDL and CropWatR

Base year (2008) acreage from BEPAM is downscaled to the resolution of 10 by 10 km according to acreages reported in the Cropland Data Layer (CDL) [1]. Crop categories were reported at different levels of specificity in BEPAM and in the CDL, and there were minor mismatches in total acreage as estimated by the model and by the satellite estimates for the base year. To address the later mismatch, cropped areas reported in the CDL are adjusted by scalars at the CRD resolution to match the CRD-level estimated by BEPAM in 2008. Rotations of corn and soybean modeled by BEPAM are allocated assumed a 50:50 split between corn and soybeans. To reclassify and match crop categories, areas cropped in BEPAM in wheat are allocated among spring, durum, and winter wheat proportionally according to acreage in the CDL; spring and fall varieties of oats and barley were split by seasonal variety according to state-level acreage splits as reported by NASS [2, 3] (in most states, only the spring or fall variety is grown). Alfalfa and hay acreages modeled in BEPAM were allocated to areas cropped in the 2008 CDL first to alfalfa (30.27 million hectares or 12.25 million acres), then to ‘other (non-alfalfa) hay’ (40.45 million hectares or 16.37 million acres) and finally to ‘pasture hay’ (74.13 million hectares or 30 million acres) categories such that total CRD cropped modeled in the base year areas matched BEPAM inputs. S1 Table A shows the translation of crops and land use categories modeled by BEPAM and included in the CDL to varieties and categories used for crop-water modeling in *CropWatR*.

**S1 Table A.** **Crop and land use categories modeled in BEPAM, CDL, and CropWatR.**

| BEPAM | CDL | CropWatR |
| --- | --- | --- |
| Corn/Soybean | Corn/Soybean | Corn/Soybean |
| Corn | Corn | Corn |
| Soybeans | Soybeans | Soybeans |
| Wheat | Durum wheat, spring wheat, winter wheat | Durum wheat, spring wheat, winter wheat |
| Sorghum | Sorghum | Sorghum |
| Barley | Barley | Spring barley, fall barley |
| Cotton | Cotton | Cotton |
| Oats | Oats | Spring oats, fall oats |
| Peanuts | Peanuts | Peanuts |
| Rice | Rice | Rice |
| Silage | -- | Silage |
| Sugar beets | Sugar beets | Sugar beets |
| Sugarcane | Sugarcane | Sugarcane |
| Alfalfa | Alfalfa, other hay, pasture/hay | Alfalfa-hay |

#### I.b. Land use change under the BAU and policy scenarios

**S1 Table B.** **Area cropped in million hectares in the base year (2008) and at the end of the modeling period by scenario.**

| Crop | 2008  (Base Year) | End of modeling period (2030) | | | | |
| --- | --- | --- | --- | --- | --- | --- |
|  |  | BAU | Mandate | Δ | CFS | Δ |
| Winter wheat | 14.20 | 18.25 | 17.56 | -4% | 15.78 | -13% |
| Alfalfa & other hays | 23.76 | 22.99 | 22.66 | -1% | 21.85 | -5% |
| Corn | 32.58 | 28.85 | 36.22 | 26% | 30.51 | 6% |
| Soybeans | 29.06 | 29.54 | 25.37 | -14% | 27.15 | -8% |
| Switchgrass |  |  | 0.02 |  | 1.54 |  |
| *regular cropland* |  |  | 0.02 |  | 0.43 |  |
| *cropland pasture* |  |  |  |  | 1.10 |  |
| Miscanthus |  |  | 1.94 |  | 10.72 |  |
| *regular cropland* |  |  | 0.43 |  | 5.50 |  |
| *cropland pasture* |  |  | 1.51 |  | 5.22 |  |
| Other Crops | 15.09 | 19.87 | 18.33 | -8% | 18.66 | -6% |
| Total Acreage | 114.73 | 119.46 | 122.13 | 2% | 126.18 | 6% |

Delta values show the percent change in the policy scenario compared with the BAU.

## Land use by crop

S1 Figs A-D show the areas cropped in row crops in the base year (2008) (S1 Fig A), and at the end of the modeling period in each of the three biofuel policy scenarios: the no-policy counterfactual (BAU) (S1 Fig B), the Mandate (S1 Fig C), and the CFS (S1 Fig D) scenarios.

Land cropped in switchgrass and miscanthus in the end of the modeling period (2030) in the Mandate scenario is shown in S1 Fig E, and in the CFS scenario in S1 Fig F. Both miscanthus and switchgrass can, in principle, be grown on either marginal land or on regular cropland. In the RFS, miscanthus is grown on both types of land, while switchgrass is grown only on regular cropland. The total land cropped in miscanthus or switchgrass is the sum across land types, and the total land cropped in dedicated feedstocks for cellulosic ethanol is the sum across land types. In the Mandate scenario, switchgrass is grown on regular land only in some watersheds of Texas. Comparing the four figures in S1 Fig F with the three shown in S1 Fig E, we see that the distribution of regular cropland and marginal land cropped in switchgrass and especially in miscanthus is much more extensive in the CFS than in the Mandate scenario.

Land use change aggregated across all crops, including displacement of cropland pasture by energy crops, is shown in S1 Fig F. Relative to the BAU scenario, the cropped acreage increases in both the CFS and the Mandate scenarios. The increases are concentrated in the Midwest and Northern and Southern Great Plains under both scenarios – Oklahoma, Kansas, Nebraska, and North and South Dakota, as well as Kentucky, Tennessee, and Wisconsin in the Mandate, experience the greatest increases in cropped area. More moderate increases in the area of cropped land occur throughout the Corn Belt. The CFS scenario leads to pronounced increases in land dedicated to switchgrass cultivation throughout Texas, and to miscanthus throughout the eastern U.S. (and in particular in Oklahoma and along the mid stretches of the Mississippi river).

In addition to the crop area expansion, price driven crop substitution, and cultivation of energy crops on marginal lands, the CFS and Mandate scenarios alter the net balance of crop management practices at county, regional, and national levels. The area of irrigated corn increases in both scenarios relative to the BAU (S1 Table C). However, these area increases of 0.386 and 0.230 million hectares, respectively, are small compared to the overall increase in corn area (shown in S1 Table C) of 7.37 and 1.66 million hectares, respectively.

A similar pattern is apparent for soybeans and other row crops (e.g. cotton, alfalfa, rice): decreases in overall acreage due to substitution by corn and energy crops are greater than decreases in irrigated cropland by about an order of magnitude. A notable exception to this trend is wheat: in this case decreases in total acreage are countered by an increase in irrigated acreage. The general pattern that emerges is that crops are displaced from regions where rainfed cultivation is economical (e.g. the Midwest, Corn Belt, and Southeast) to the Great Plains (in particular Oklahoma, Kansas, Nebraska, and North and South Dakota) where irrigation is necessary for economic viability.

**S1 Fig A.**

**S1 Fig B.**

**S1 Fig C.**

**S1 Fig D.**

**S1 Fig E.**

**S1 Fig F.**

**S1 Fig G.**

**S1 Table C.** **Million hectares irrigated at the end of the modeling period in each scenario.**

| Crop | BAU | Mandate | Δ | CFS | Δ |
| --- | --- | --- | --- | --- | --- |
| Corn | 3.28 | 3.65 | 11% | 3.51 | 7% |
| Soybeans | 1.93 | 1.49 | -23% | 1.70 | -12% |
| Wheat (spring & winter) | 1.78 | 1.83 | 3% | 1.83 | 3% |
| Cotton | 1.46 | 1.40 | -4% | 1.42 | -2% |
| Alfalfa | 1.38 | 1.34 | -3% | 1.36 | -1% |
| Rice | 1.12 | 1.06 | -5% | 1.10 | -2% |
| Sorghum | 0.42 | 0.41 | -3% | 0.42 | 0% |
| Spring barley | 0.43 | 0.45 | 3% | 0.43 | 0% |
| Silage | 0.17 | 0.15 | -9% | 0.16 | -6% |
| Spring oats | 0.02 | 0.02 | 12% | 0.02 | 2% |
| Peanuts | 0.08 | 0.08 | 11% | 0.08 | 0% |
| All Crops | 12.06 | 11.87 | -2% | 12.03 | -0.3% |

Nationwide changes in irrigated area, by crop, in million hectares. Deltas are the percent difference between the Mandate and CFS scenarios from the counterfactual (no-policy BAU) at the end of the modeling period, respectively.

## II. More Detailed Results about Water Balances

The figure below illustrates the key water flows modeled in *CropWatR*: (i) *irrigation* (volumes applied at the crop roots); (ii) *evaporation,* (iii) *transpiration* (including *off-season transpiration* of weed/other non-harvested plants (sometimes referred to as non-productive water use) and *transpiration of cultivated crops* (sometimes referred to as productive water use); (iv) *runoff*, and; (v) *groundwater infiltration*. Irrigation is often referred to as blue water (BW) use, whereas the sum of (growing season) evaporation and transpiration are often referred to as the green water (GW) consumption that is associated with crop production.

**S1 Fig H.**

**II.a. Water balances**

**S1 Fig I.**

**S1 Fig J.**

## II.b Geographic distribution of water use intensity, by feedstock and scenario

S7 Figs K-N shows the green- and blue water use intensity, in liters per MJ of biofuel product, for corn grain and soybeans in the base year (2008), and for corn grain, soybeans, switchgrass, and miscanthus, at the end of the modeling period, in each of the three biofuel policy scenarios: the no-policy counterfactual (BAU), the Mandate, and the CFS scenario.

In a few locations, soybeans (in the base year [2008] and in the BAU scenario) and corn (in the BAU scenario) are not provided with sufficient water to grow very much, as modeled in CropWatR. This is a result of the calibration of irrigation regimes to statewide averages (see SI 2 for further details on this calibration), together insufficient rainfall in these regions. The result is that the crop transpiration water use intensity in these regions is very close to zero – from very little biomass yeild comes very little biofuel product.

**S1 Fig K.**

**S1 Fig L.**

**S1 Fig M.**

**S1 Fig N.**

#### III. The CropWatR Model

CropWatR is a process-based crop-water model that can be used for high spatial and temporal resolution estimation of crop-water balances in large regions. It can be used to designate simple irrigation scheduling rules, separately model evaporation and transpiration, and estimate volumes of seasonal and annual runoff and groundwater infiltration.

The implementation of the model program in *R* is quite similar to the FAO CropWat model,[4] with a few key differences, including: (1) the parameters can easily be calibrated by the user to match local conditions, (2) input parameters can be specified to match survey or GIS data, and (3) the user can easily incorporate geographic data to batch process calculations across varying locations with different weather, soil, and management parameters.

The model runs on a daily time step and estimates balances of transpired and evaporated water, groundwater infiltration and runoff, and volumes of irrigation water applied. To model each of these key water flows, it uses as its basis the algorithms for computation of the dual crop coefficient (Kcb) as described in the report by the Food and Agriculture Organization of the United Nations, “*Crop evapotranspiration - Guidelines for computing crop water requirements - FAO Irrigation and drainage paper 56.*”[5] The model can be specified to match planting and harvesting dates, total annual irrigation application volumes, and other parameters, based on field or survey data. Crop coefficients and other parameters can be calibrated to match and validated against field measurements and/or other estimates of evapotranspiration. The model can further be used to estimate water balances in the off (or fallow) season, as well as for perennial or non-crop land uses (such as idle crop land or pasture land).

Running the model run requires that daily reference evapotranspiration (ETo) is first derived on the basis of daily weather data using the full Penman-Monteith algorithm. Next, survey and satellite data can be used to specify key parameters (e.g. planting and harvesting dates, irrigation intensity, and land area irrigated). Area cropped per grid cell serves as a mask to determine the grid cells for which the daily computation of crop-water balances is run, for each crop.

A complete description of CropWatR can be found in a separate second document of supporting information.[6] The source code for implementing the algorithms using the dual crop coefficient described in FAO 56 and integrating these with geographic and survey data is available on github at *github.com/jacobteter/CropWatR*.

#### IV. Literature estimates of blue and green water use for biofuel feedstock cultivation

**S1 Table D.** **Literature estimates of blue and green water use for cultivation of biofuels feedstock.**

**Corn grain ethanol**

| Region | Water type | LCA stage(s) | Liters water/MJ ethanol | Source |
| --- | --- | --- | --- | --- |
| Major corn producing regions | Blue water  (irrigation) | Cultivation | **0.33** in Region 5  (IA, IL, IN, OH, MO)  **0.65** in Region 6  (MN, WI, MI)  **15.19** in Region 7  (SD, ND, KS, NB) | [7] |
| Minnesota | Blue water | Cultivation and Production | **0.57-1.33** | [8] |
| Major corn producing regions | Blue water | Cultivation and Production | **0.24-2.70** in Region 5  (IA, IL, IN, OH, MO)  **0.90-2.23** in Region 6  (MN, WI, MI)  **2.8 – 25.0** in Region 7  (SD, ND, KS, NB) | [9] |
| US average | Blue and green water (ET) | Cultivation | **60** | [10] |

**Soybean biodiesel**

| Region | Water type | LCA stage(s) | Liters water/MJ | Source |
| --- | --- | --- | --- | --- |
| US average | Blue and green water  (ET) | Cultivation | **198** | [10] |

**Miscanthus ethanol**

| Region | Water type | LCA stage(s) | Liters water/MJ ethanol | Source |
| --- | --- | --- | --- | --- |
| US | Blue and green water (ET) | Cultivation | **95-620** | [11] |
| US corn regions | Blue and green water (ET) | Cultivation | **26-39** | [12] |

**Switchgrass ethanol**

| Region | Water type | LCA stage(s) | Liters water/MJ ethanol | Source |
| --- | --- | --- | --- | --- |
| US | Blue and green water (ET) | Cultivation | **95-284** | [11] |
| US corn regions | Blue and green water (ET) | Cultivation | **59-95** | [12] |

**References**

1. Han W, Yang Z, Di L, Mueller R. CropScape: A Web service based application for exploring and disseminating US conterminous geospatial cropland data products for decision support. Computers and Electronics in Agriculture. 2012;84:111-23.

2. NASS U. Census of agriculture. US Department of Agriculture, National Agricultural Statistics Service, Washington, DC. 2007.

3. NASS U. Farm and ranch irrigation survey. US Department of Agriculture, National Agricultural Statistics Service. 2008.

4. FAO. CROPWAT 8.0: A computer program for irrigation planning and management. Irrigation and Drainage Paper. 2012;46.

5. Allen RG, Pereira LS, Smith M, Raes D, Wright JL. FAO-56 dual crop coefficient method for estimating evaporation from soil and application extensions. Journal of irrigation and drainage engineering. 2005;131(1):2-13.

6. Teter J. The Agricultural Water Use Impacts of Biofuel Cultivation in the United States, and of California’s Future Transportation Fuels: Universtiy of California, Davis; 2015.

7. Wu M, Mintz M, Wang M, Arora S. Water Consumption in the Production of Ethanol and Petroleum Gasoline. Environmental Management. 2009;44(5):981-97.

8. Chiu Y-W, Suh S, Pfister S, Hellweg S, Koehler A. Measuring ecological impact of water consumption by bioethanol using life cycle impact assessment. The International Journal of Life Cycle Assessment. 2012;17(1):16-24. doi: 10.1007/s11367-011-0328-0.

9. Chiu Y-W, Walseth B, Suh S. Water embodied in bioethanol in the United States. Environmental Science & Technology. 2009;43(8):2688-92. doi: 10.1021/es8031067.

10. Dominguez-Faus R, Powers SE, Burken JG, Alvarez PJ. The water footprint of biofuels: A drink or drive issue? Environmental Science & Technology. 2009;43(9):3005-10. doi: 10.1021/es802162x.

11. Song Y, Cervarich M, Jain AK, Kheshgi HS, Landuyt W, Cai X. The Interplay Between Bioenergy Grass Production and Water Resources in the United States of America. Environmental Science & Technology. 2016. doi: 10.1021/acs.est.5b05239.

12. Zhuang Q, Qin Z, Chen M. Biofuel, land and water: maize, switchgrass orMiscanthus? Environmental Research Letters. 2013;8(1):015020. doi: 10.1088/1748-9326/8/1/015020.
