# Supplementary figures and images for: Water impacts of U.S. biofuels: Insights from an assessment combining economic and biophysical models
Source: PLoS One. 2018 Sep 28;13(9):e0204298. doi: 10.1371/journal.pone.0204298 (PMC6161887; doi:10.1371/journal.pone.0204298)

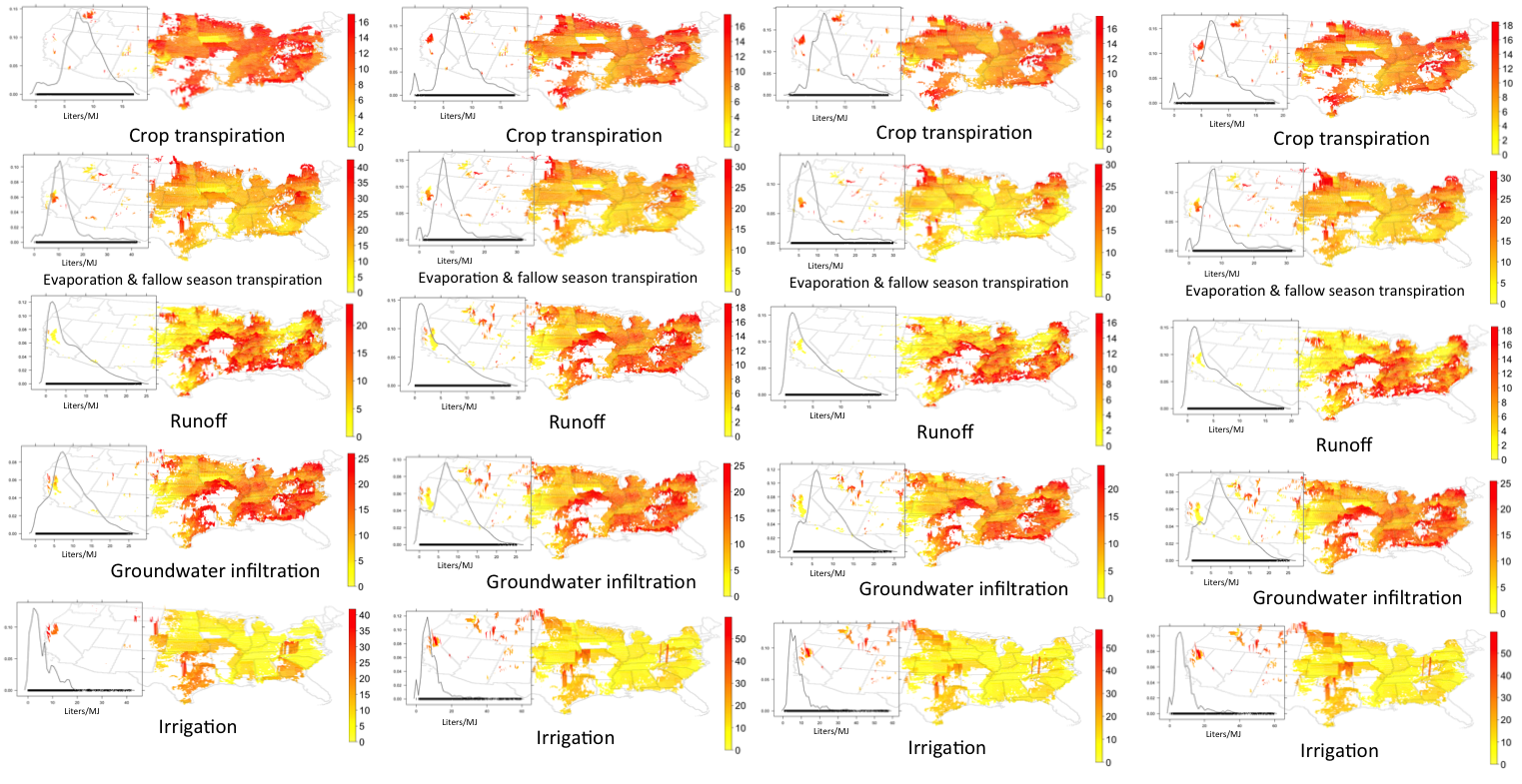

Supplement: S1 File — Table A. Crop and land use categories modeled in BEPAM, CDL, and CropWatR. Table B. Area cropped in million hectares in the base year (2008) and at the end of the modeling period by scenario. Delta values show the percent change in the policy scenario compared with the BAU. Table C. Million hectares irrigated at the end of the modeling period in each scenario. Nationwide changes in irrigated area, by crop, in million hectares. Deltas are the percent difference between the Mandate and CFS scenarios from the counterfactual (no-policy BAU) at the end of the modeling period, respectively. Table D. Literature estimates of blue and green water use for cultivation of biofuels feedstock. Fig A. Base Year (2008) cropping patterns. Colors indicate the percent of land cropped in each 10 x 10 kilometer pixel. Fig B. BAU cropping patterns at the end of the modeling period, in 2030. Fig C. Mandate scenario cropping patterns at the end of the modeling period, in 2030. Fig D. CFS scenario cropping patterns at the end of the modeling period, in 2030. Fig E. Land converted for cellulosic feedstocks in the Mandate scenario. Area cultivated in miscanthus and switchgrass as a percentage of total regular cropland and marginal land, at the resolution of 10 x 10 kilometers at the end of the modeling period. Total land cropped in miscanthus is the sum of land cropped in regular cropland and in marginal land. Switchgrass is cropped only on regular cropland in the Mandate scenario. Fig F. Land converted for cellulosic feedstocks in the CFS scenario. Area cultivated in miscanthus and switchgrass as a percentage of total land, at the resolution of 10 x 10 kilometers at the end of the modeling period. Total land cropped in both miscanthus and switchgrass is the sum of land cropped in regular cropland and in marginal land. Fig G. Land use change (increase or decrease in cropland, at 10 x 10 km resolution) in the Mandate (top) and CFS (bottom) scenarios, relative to the no-policy counterfactual (B [file pone.0204298.s001.zip › SI 1 Figures Tables/S1 Fig K.tiff]

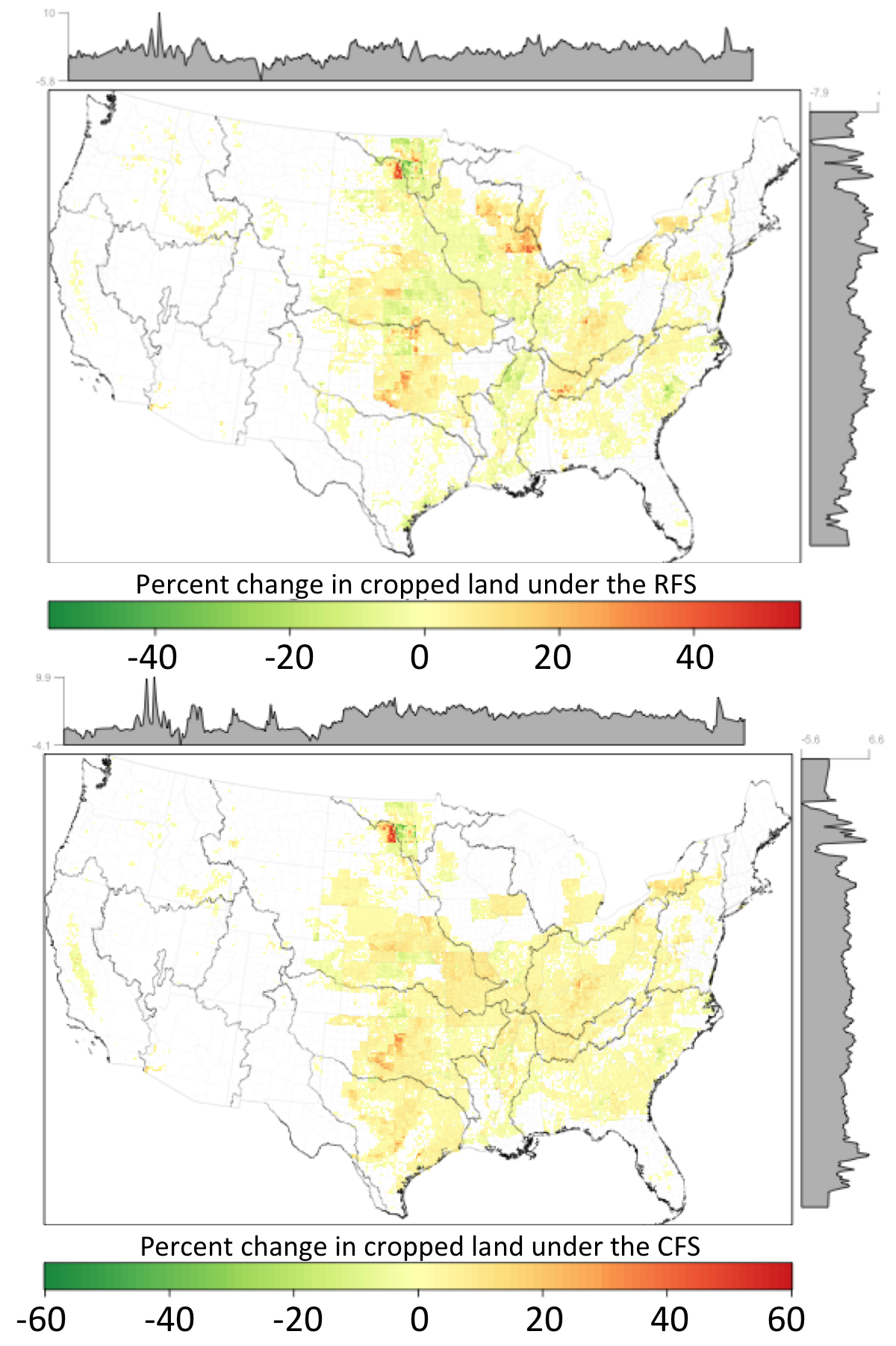

Supplement: S1 File — Table A. Crop and land use categories modeled in BEPAM, CDL, and CropWatR. Table B. Area cropped in million hectares in the base year (2008) and at the end of the modeling period by scenario. Delta values show the percent change in the policy scenario compared with the BAU. Table C. Million hectares irrigated at the end of the modeling period in each scenario. Nationwide changes in irrigated area, by crop, in million hectares. Deltas are the percent difference between the Mandate and CFS scenarios from the counterfactual (no-policy BAU) at the end of the modeling period, respectively. Table D. Literature estimates of blue and green water use for cultivation of biofuels feedstock. Fig A. Base Year (2008) cropping patterns. Colors indicate the percent of land cropped in each 10 x 10 kilometer pixel. Fig B. BAU cropping patterns at the end of the modeling period, in 2030. Fig C. Mandate scenario cropping patterns at the end of the modeling period, in 2030. Fig D. CFS scenario cropping patterns at the end of the modeling period, in 2030. Fig E. Land converted for cellulosic feedstocks in the Mandate scenario. Area cultivated in miscanthus and switchgrass as a percentage of total regular cropland and marginal land, at the resolution of 10 x 10 kilometers at the end of the modeling period. Total land cropped in miscanthus is the sum of land cropped in regular cropland and in marginal land. Switchgrass is cropped only on regular cropland in the Mandate scenario. Fig F. Land converted for cellulosic feedstocks in the CFS scenario. Area cultivated in miscanthus and switchgrass as a percentage of total land, at the resolution of 10 x 10 kilometers at the end of the modeling period. Total land cropped in both miscanthus and switchgrass is the sum of land cropped in regular cropland and in marginal land. Fig G. Land use change (increase or decrease in cropland, at 10 x 10 km resolution) in the Mandate (top) and CFS (bottom) scenarios, relative to the no-policy counterfactual (B [file pone.0204298.s001.zip › SI 1 Figures Tables/S1 Fig G.tiff]

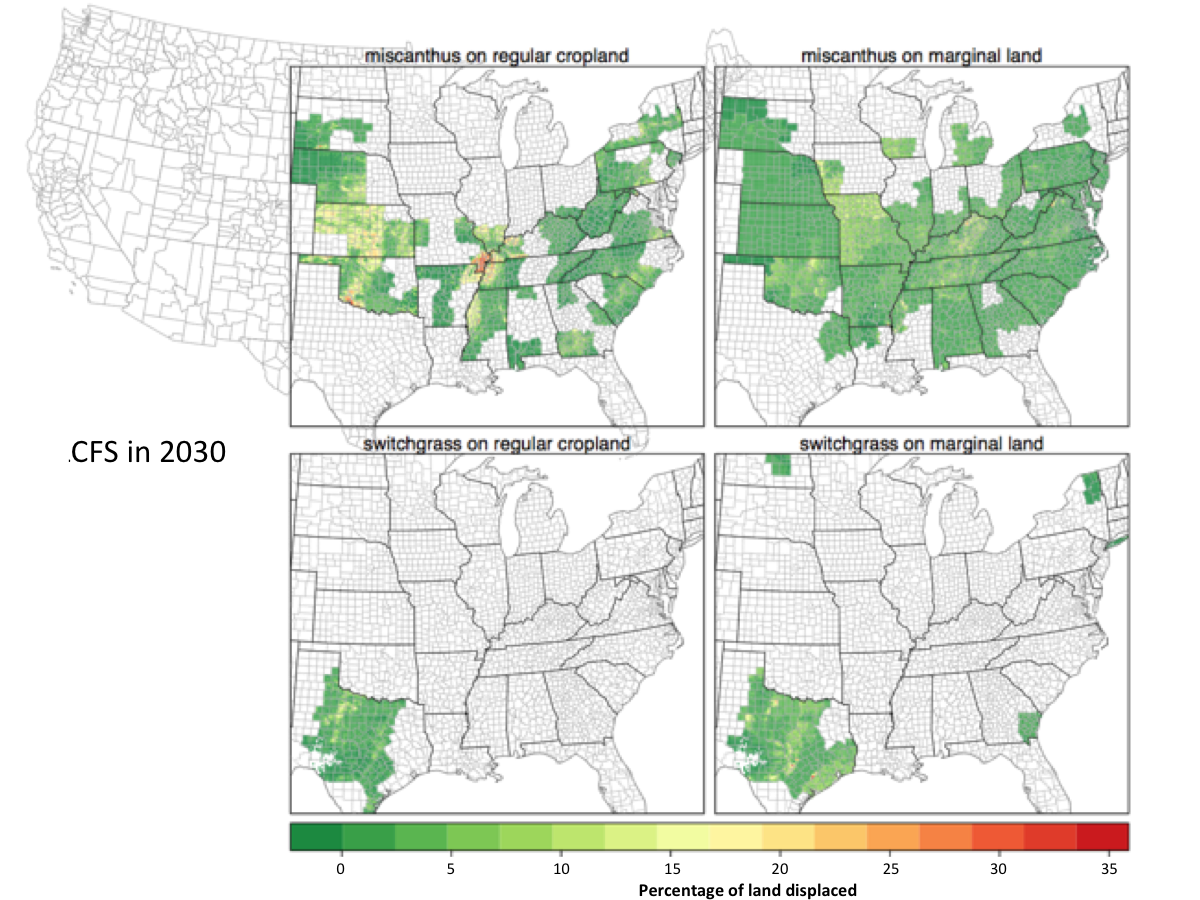

Supplement: S1 File — Table A. Crop and land use categories modeled in BEPAM, CDL, and CropWatR. Table B. Area cropped in million hectares in the base year (2008) and at the end of the modeling period by scenario. Delta values show the percent change in the policy scenario compared with the BAU. Table C. Million hectares irrigated at the end of the modeling period in each scenario. Nationwide changes in irrigated area, by crop, in million hectares. Deltas are the percent difference between the Mandate and CFS scenarios from the counterfactual (no-policy BAU) at the end of the modeling period, respectively. Table D. Literature estimates of blue and green water use for cultivation of biofuels feedstock. Fig A. Base Year (2008) cropping patterns. Colors indicate the percent of land cropped in each 10 x 10 kilometer pixel. Fig B. BAU cropping patterns at the end of the modeling period, in 2030. Fig C. Mandate scenario cropping patterns at the end of the modeling period, in 2030. Fig D. CFS scenario cropping patterns at the end of the modeling period, in 2030. Fig E. Land converted for cellulosic feedstocks in the Mandate scenario. Area cultivated in miscanthus and switchgrass as a percentage of total regular cropland and marginal land, at the resolution of 10 x 10 kilometers at the end of the modeling period. Total land cropped in miscanthus is the sum of land cropped in regular cropland and in marginal land. Switchgrass is cropped only on regular cropland in the Mandate scenario. Fig F. Land converted for cellulosic feedstocks in the CFS scenario. Area cultivated in miscanthus and switchgrass as a percentage of total land, at the resolution of 10 x 10 kilometers at the end of the modeling period. Total land cropped in both miscanthus and switchgrass is the sum of land cropped in regular cropland and in marginal land. Fig G. Land use change (increase or decrease in cropland, at 10 x 10 km resolution) in the Mandate (top) and CFS (bottom) scenarios, relative to the no-policy counterfactual (B [file pone.0204298.s001.zip › SI 1 Figures Tables/S1 Fig F.tiff]

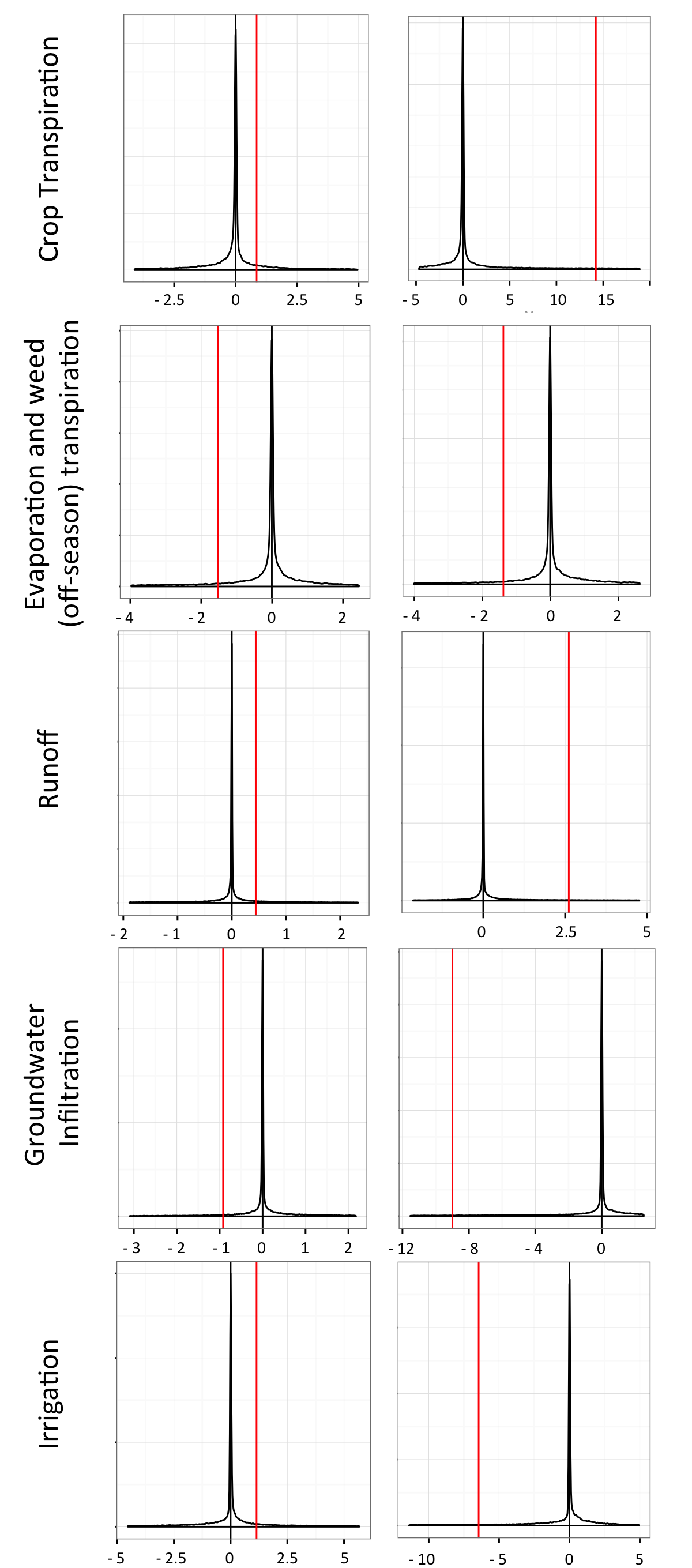

Supplement: S1 File — Table A. Crop and land use categories modeled in BEPAM, CDL, and CropWatR. Table B. Area cropped in million hectares in the base year (2008) and at the end of the modeling period by scenario. Delta values show the percent change in the policy scenario compared with the BAU. Table C. Million hectares irrigated at the end of the modeling period in each scenario. Nationwide changes in irrigated area, by crop, in million hectares. Deltas are the percent difference between the Mandate and CFS scenarios from the counterfactual (no-policy BAU) at the end of the modeling period, respectively. Table D. Literature estimates of blue and green water use for cultivation of biofuels feedstock. Fig A. Base Year (2008) cropping patterns. Colors indicate the percent of land cropped in each 10 x 10 kilometer pixel. Fig B. BAU cropping patterns at the end of the modeling period, in 2030. Fig C. Mandate scenario cropping patterns at the end of the modeling period, in 2030. Fig D. CFS scenario cropping patterns at the end of the modeling period, in 2030. Fig E. Land converted for cellulosic feedstocks in the Mandate scenario. Area cultivated in miscanthus and switchgrass as a percentage of total regular cropland and marginal land, at the resolution of 10 x 10 kilometers at the end of the modeling period. Total land cropped in miscanthus is the sum of land cropped in regular cropland and in marginal land. Switchgrass is cropped only on regular cropland in the Mandate scenario. Fig F. Land converted for cellulosic feedstocks in the CFS scenario. Area cultivated in miscanthus and switchgrass as a percentage of total land, at the resolution of 10 x 10 kilometers at the end of the modeling period. Total land cropped in both miscanthus and switchgrass is the sum of land cropped in regular cropland and in marginal land. Fig G. Land use change (increase or decrease in cropland, at 10 x 10 km resolution) in the Mandate (top) and CFS (bottom) scenarios, relative to the no-policy counterfactual (B [file pone.0204298.s001.zip › SI 1 Figures Tables/S1 Fig J.tiff]

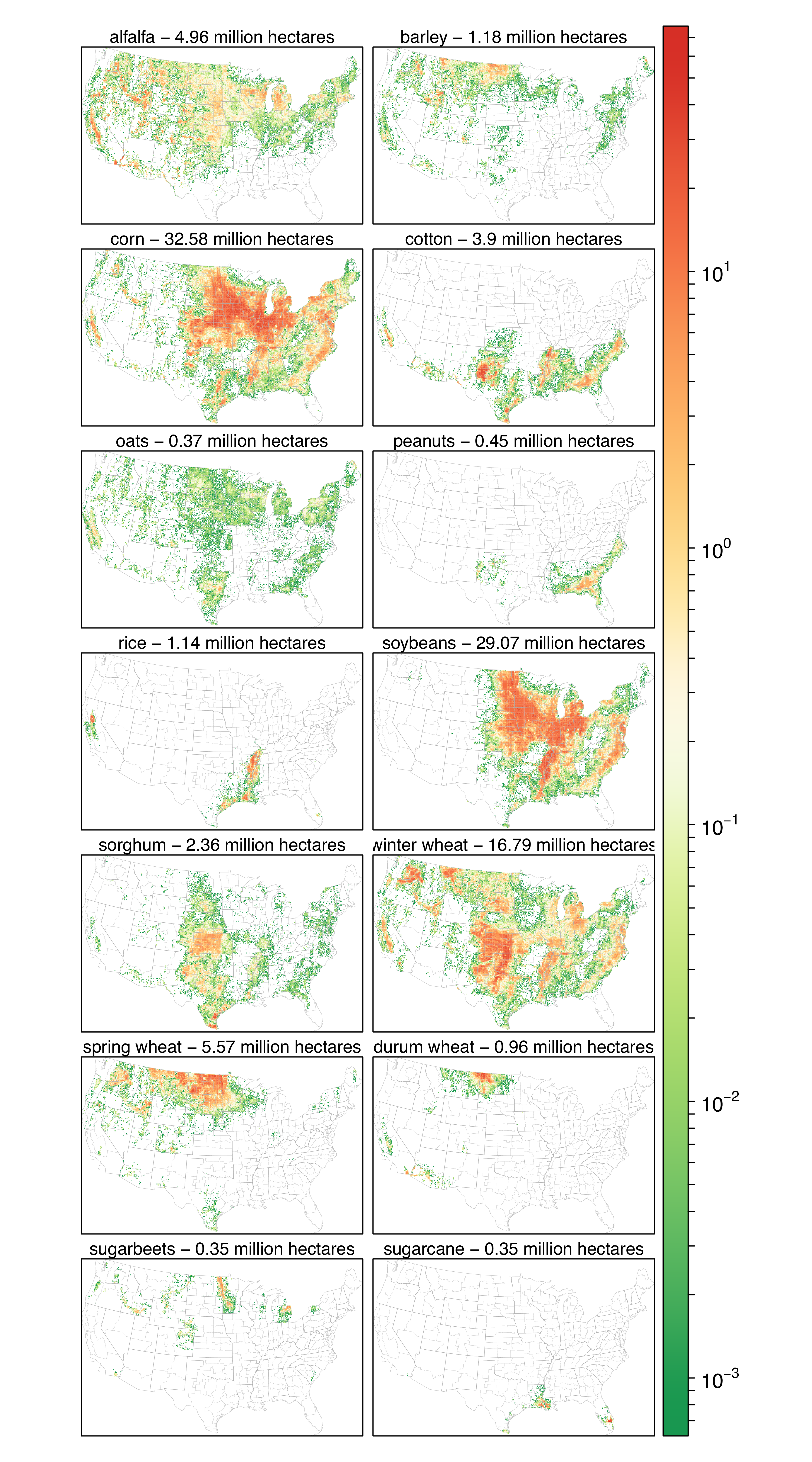

Supplement: S1 File — Table A. Crop and land use categories modeled in BEPAM, CDL, and CropWatR. Table B. Area cropped in million hectares in the base year (2008) and at the end of the modeling period by scenario. Delta values show the percent change in the policy scenario compared with the BAU. Table C. Million hectares irrigated at the end of the modeling period in each scenario. Nationwide changes in irrigated area, by crop, in million hectares. Deltas are the percent difference between the Mandate and CFS scenarios from the counterfactual (no-policy BAU) at the end of the modeling period, respectively. Table D. Literature estimates of blue and green water use for cultivation of biofuels feedstock. Fig A. Base Year (2008) cropping patterns. Colors indicate the percent of land cropped in each 10 x 10 kilometer pixel. Fig B. BAU cropping patterns at the end of the modeling period, in 2030. Fig C. Mandate scenario cropping patterns at the end of the modeling period, in 2030. Fig D. CFS scenario cropping patterns at the end of the modeling period, in 2030. Fig E. Land converted for cellulosic feedstocks in the Mandate scenario. Area cultivated in miscanthus and switchgrass as a percentage of total regular cropland and marginal land, at the resolution of 10 x 10 kilometers at the end of the modeling period. Total land cropped in miscanthus is the sum of land cropped in regular cropland and in marginal land. Switchgrass is cropped only on regular cropland in the Mandate scenario. Fig F. Land converted for cellulosic feedstocks in the CFS scenario. Area cultivated in miscanthus and switchgrass as a percentage of total land, at the resolution of 10 x 10 kilometers at the end of the modeling period. Total land cropped in both miscanthus and switchgrass is the sum of land cropped in regular cropland and in marginal land. Fig G. Land use change (increase or decrease in cropland, at 10 x 10 km resolution) in the Mandate (top) and CFS (bottom) scenarios, relative to the no-policy counterfactual (B [file pone.0204298.s001.zip › SI 1 Figures Tables/S1 Fig A.tiff]

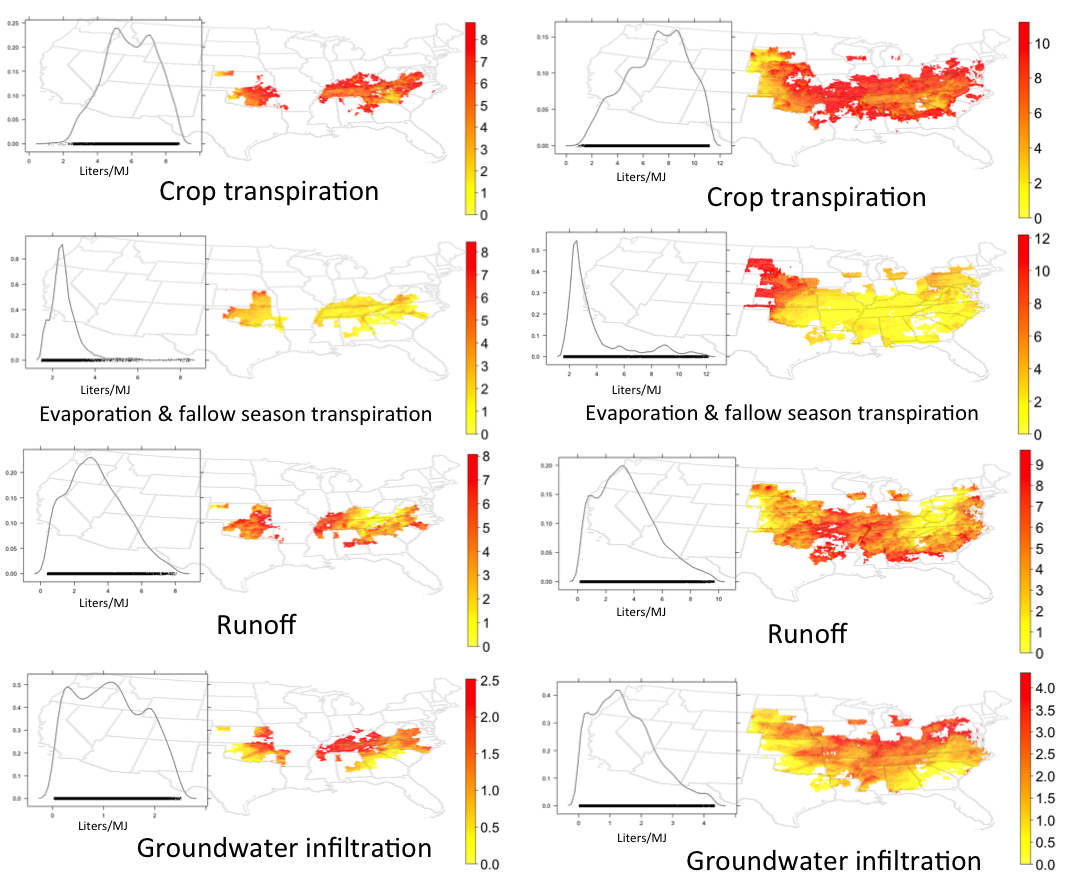

Supplement: S1 File — Table A. Crop and land use categories modeled in BEPAM, CDL, and CropWatR. Table B. Area cropped in million hectares in the base year (2008) and at the end of the modeling period by scenario. Delta values show the percent change in the policy scenario compared with the BAU. Table C. Million hectares irrigated at the end of the modeling period in each scenario. Nationwide changes in irrigated area, by crop, in million hectares. Deltas are the percent difference between the Mandate and CFS scenarios from the counterfactual (no-policy BAU) at the end of the modeling period, respectively. Table D. Literature estimates of blue and green water use for cultivation of biofuels feedstock. Fig A. Base Year (2008) cropping patterns. Colors indicate the percent of land cropped in each 10 x 10 kilometer pixel. Fig B. BAU cropping patterns at the end of the modeling period, in 2030. Fig C. Mandate scenario cropping patterns at the end of the modeling period, in 2030. Fig D. CFS scenario cropping patterns at the end of the modeling period, in 2030. Fig E. Land converted for cellulosic feedstocks in the Mandate scenario. Area cultivated in miscanthus and switchgrass as a percentage of total regular cropland and marginal land, at the resolution of 10 x 10 kilometers at the end of the modeling period. Total land cropped in miscanthus is the sum of land cropped in regular cropland and in marginal land. Switchgrass is cropped only on regular cropland in the Mandate scenario. Fig F. Land converted for cellulosic feedstocks in the CFS scenario. Area cultivated in miscanthus and switchgrass as a percentage of total land, at the resolution of 10 x 10 kilometers at the end of the modeling period. Total land cropped in both miscanthus and switchgrass is the sum of land cropped in regular cropland and in marginal land. Fig G. Land use change (increase or decrease in cropland, at 10 x 10 km resolution) in the Mandate (top) and CFS (bottom) scenarios, relative to the no-policy counterfactual (B [file pone.0204298.s001.zip › SI 1 Figures Tables/S1 Fig M.tiff]

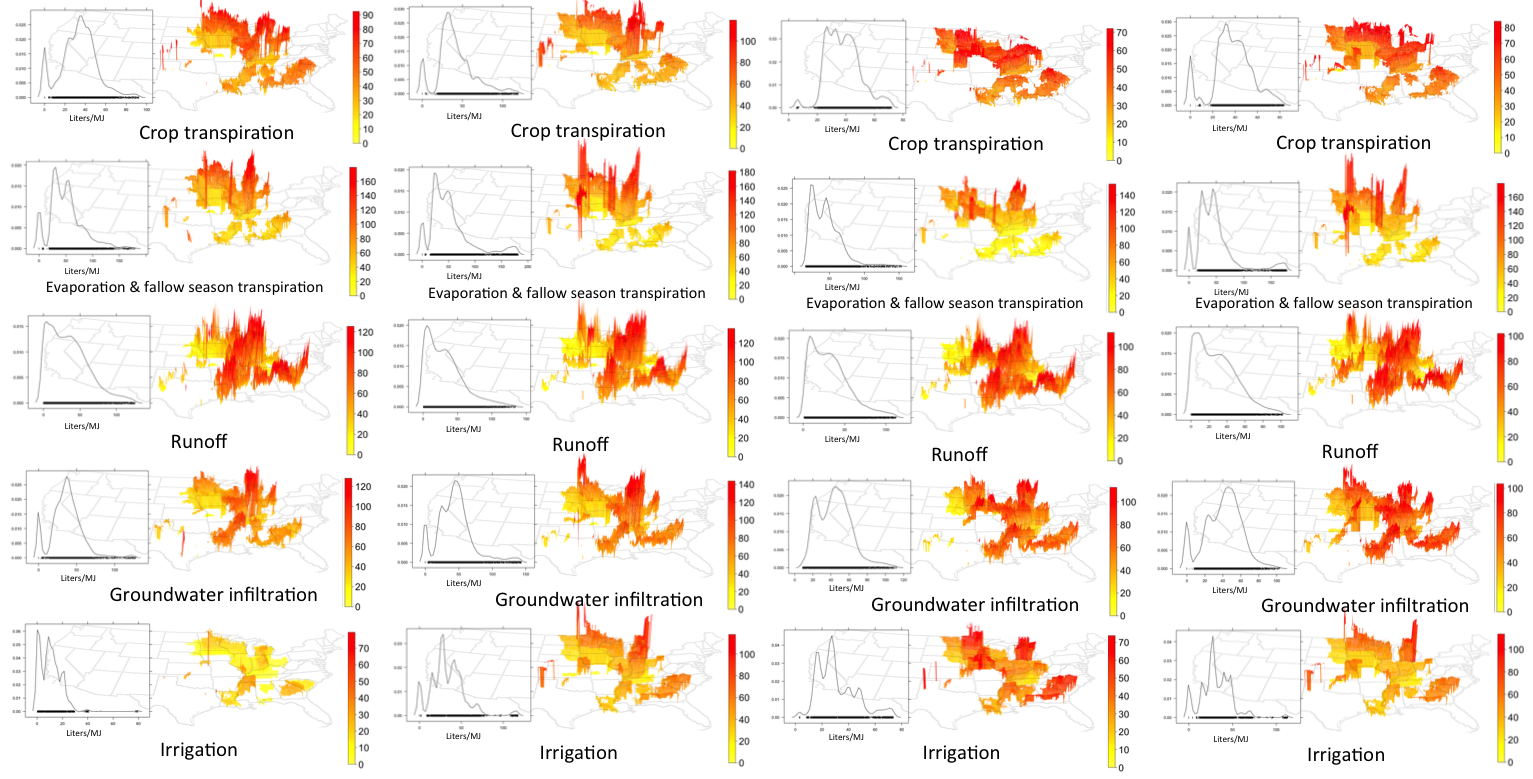

Supplement: S1 File — Table A. Crop and land use categories modeled in BEPAM, CDL, and CropWatR. Table B. Area cropped in million hectares in the base year (2008) and at the end of the modeling period by scenario. Delta values show the percent change in the policy scenario compared with the BAU. Table C. Million hectares irrigated at the end of the modeling period in each scenario. Nationwide changes in irrigated area, by crop, in million hectares. Deltas are the percent difference between the Mandate and CFS scenarios from the counterfactual (no-policy BAU) at the end of the modeling period, respectively. Table D. Literature estimates of blue and green water use for cultivation of biofuels feedstock. Fig A. Base Year (2008) cropping patterns. Colors indicate the percent of land cropped in each 10 x 10 kilometer pixel. Fig B. BAU cropping patterns at the end of the modeling period, in 2030. Fig C. Mandate scenario cropping patterns at the end of the modeling period, in 2030. Fig D. CFS scenario cropping patterns at the end of the modeling period, in 2030. Fig E. Land converted for cellulosic feedstocks in the Mandate scenario. Area cultivated in miscanthus and switchgrass as a percentage of total regular cropland and marginal land, at the resolution of 10 x 10 kilometers at the end of the modeling period. Total land cropped in miscanthus is the sum of land cropped in regular cropland and in marginal land. Switchgrass is cropped only on regular cropland in the Mandate scenario. Fig F. Land converted for cellulosic feedstocks in the CFS scenario. Area cultivated in miscanthus and switchgrass as a percentage of total land, at the resolution of 10 x 10 kilometers at the end of the modeling period. Total land cropped in both miscanthus and switchgrass is the sum of land cropped in regular cropland and in marginal land. Fig G. Land use change (increase or decrease in cropland, at 10 x 10 km resolution) in the Mandate (top) and CFS (bottom) scenarios, relative to the no-policy counterfactual (B [file pone.0204298.s001.zip › SI 1 Figures Tables/S1 Fig L.tiff]

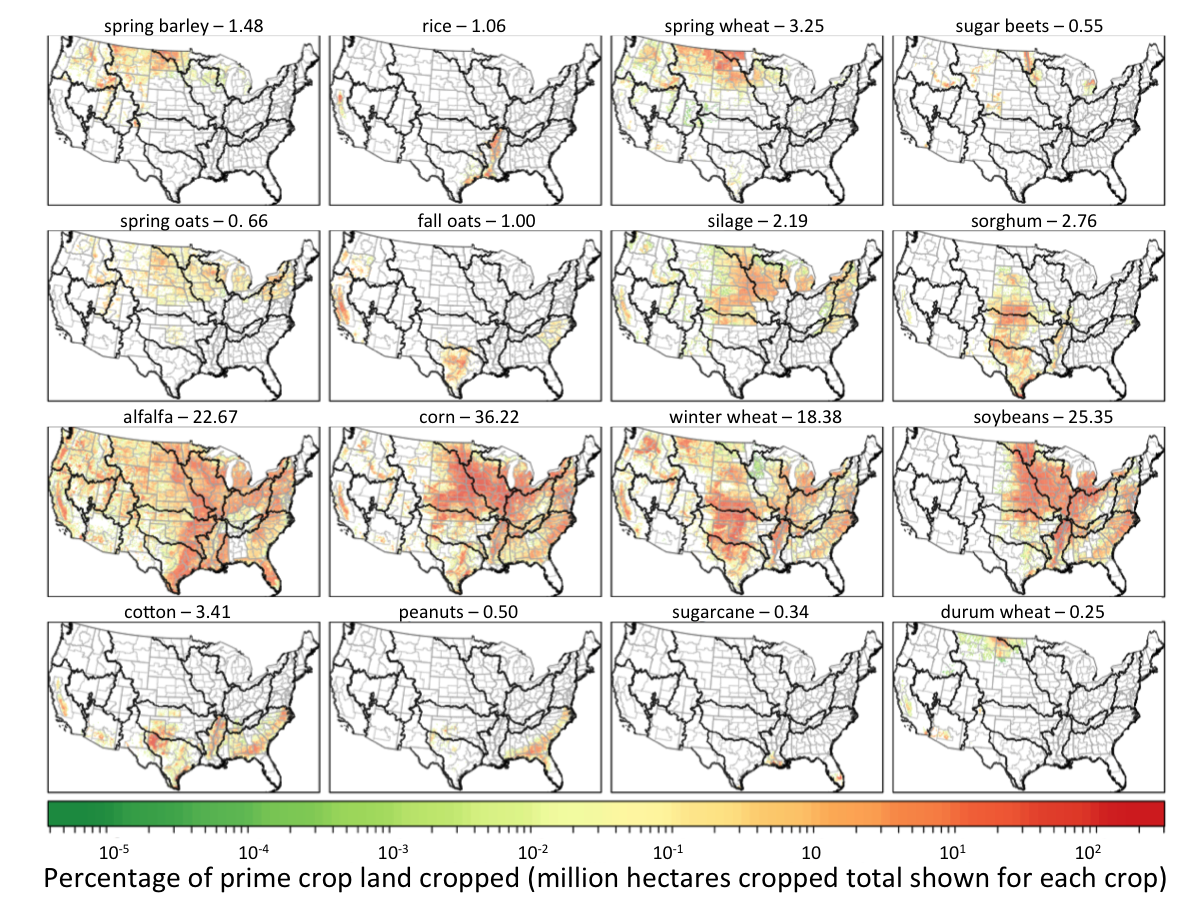

Supplement: S1 File — Table A. Crop and land use categories modeled in BEPAM, CDL, and CropWatR. Table B. Area cropped in million hectares in the base year (2008) and at the end of the modeling period by scenario. Delta values show the percent change in the policy scenario compared with the BAU. Table C. Million hectares irrigated at the end of the modeling period in each scenario. Nationwide changes in irrigated area, by crop, in million hectares. Deltas are the percent difference between the Mandate and CFS scenarios from the counterfactual (no-policy BAU) at the end of the modeling period, respectively. Table D. Literature estimates of blue and green water use for cultivation of biofuels feedstock. Fig A. Base Year (2008) cropping patterns. Colors indicate the percent of land cropped in each 10 x 10 kilometer pixel. Fig B. BAU cropping patterns at the end of the modeling period, in 2030. Fig C. Mandate scenario cropping patterns at the end of the modeling period, in 2030. Fig D. CFS scenario cropping patterns at the end of the modeling period, in 2030. Fig E. Land converted for cellulosic feedstocks in the Mandate scenario. Area cultivated in miscanthus and switchgrass as a percentage of total regular cropland and marginal land, at the resolution of 10 x 10 kilometers at the end of the modeling period. Total land cropped in miscanthus is the sum of land cropped in regular cropland and in marginal land. Switchgrass is cropped only on regular cropland in the Mandate scenario. Fig F. Land converted for cellulosic feedstocks in the CFS scenario. Area cultivated in miscanthus and switchgrass as a percentage of total land, at the resolution of 10 x 10 kilometers at the end of the modeling period. Total land cropped in both miscanthus and switchgrass is the sum of land cropped in regular cropland and in marginal land. Fig G. Land use change (increase or decrease in cropland, at 10 x 10 km resolution) in the Mandate (top) and CFS (bottom) scenarios, relative to the no-policy counterfactual (B [file pone.0204298.s001.zip › SI 1 Figures Tables/S1 Fig C.tiff]

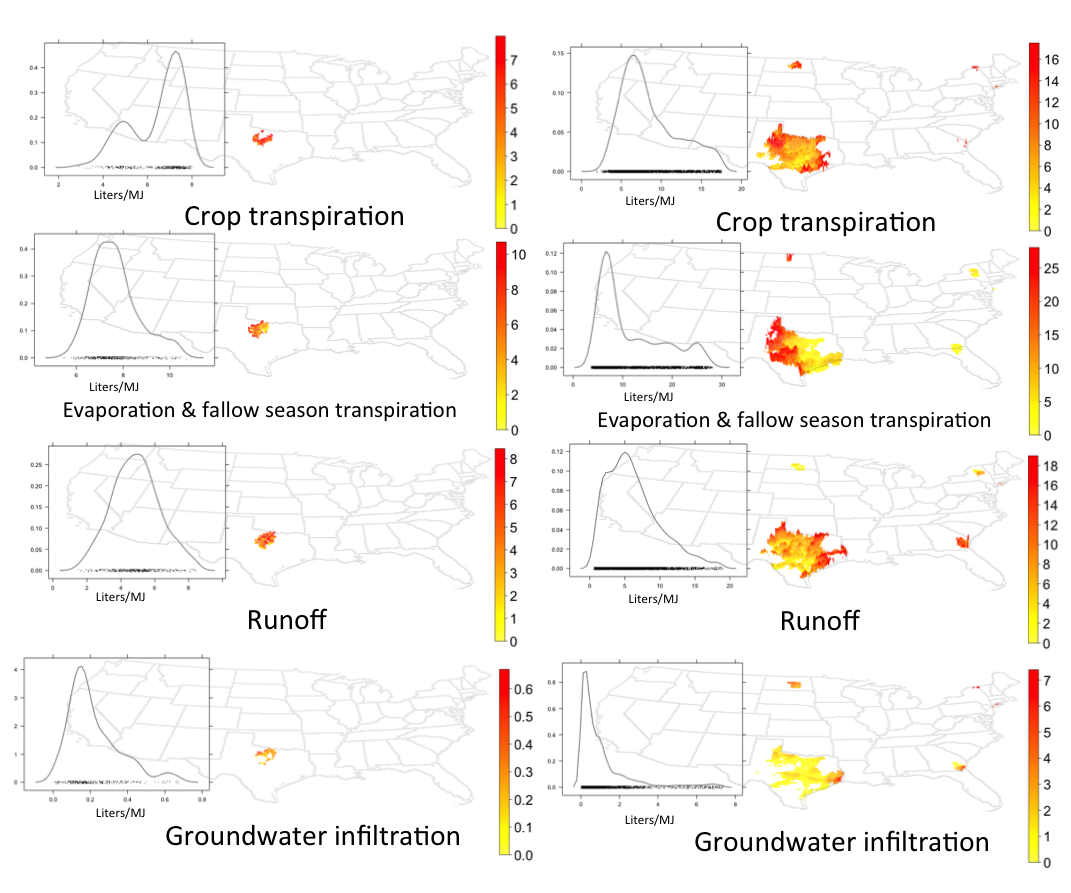

Supplement: S1 File — Table A. Crop and land use categories modeled in BEPAM, CDL, and CropWatR. Table B. Area cropped in million hectares in the base year (2008) and at the end of the modeling period by scenario. Delta values show the percent change in the policy scenario compared with the BAU. Table C. Million hectares irrigated at the end of the modeling period in each scenario. Nationwide changes in irrigated area, by crop, in million hectares. Deltas are the percent difference between the Mandate and CFS scenarios from the counterfactual (no-policy BAU) at the end of the modeling period, respectively. Table D. Literature estimates of blue and green water use for cultivation of biofuels feedstock. Fig A. Base Year (2008) cropping patterns. Colors indicate the percent of land cropped in each 10 x 10 kilometer pixel. Fig B. BAU cropping patterns at the end of the modeling period, in 2030. Fig C. Mandate scenario cropping patterns at the end of the modeling period, in 2030. Fig D. CFS scenario cropping patterns at the end of the modeling period, in 2030. Fig E. Land converted for cellulosic feedstocks in the Mandate scenario. Area cultivated in miscanthus and switchgrass as a percentage of total regular cropland and marginal land, at the resolution of 10 x 10 kilometers at the end of the modeling period. Total land cropped in miscanthus is the sum of land cropped in regular cropland and in marginal land. Switchgrass is cropped only on regular cropland in the Mandate scenario. Fig F. Land converted for cellulosic feedstocks in the CFS scenario. Area cultivated in miscanthus and switchgrass as a percentage of total land, at the resolution of 10 x 10 kilometers at the end of the modeling period. Total land cropped in both miscanthus and switchgrass is the sum of land cropped in regular cropland and in marginal land. Fig G. Land use change (increase or decrease in cropland, at 10 x 10 km resolution) in the Mandate (top) and CFS (bottom) scenarios, relative to the no-policy counterfactual (B [file pone.0204298.s001.zip › SI 1 Figures Tables/S1 Fig N.tiff]

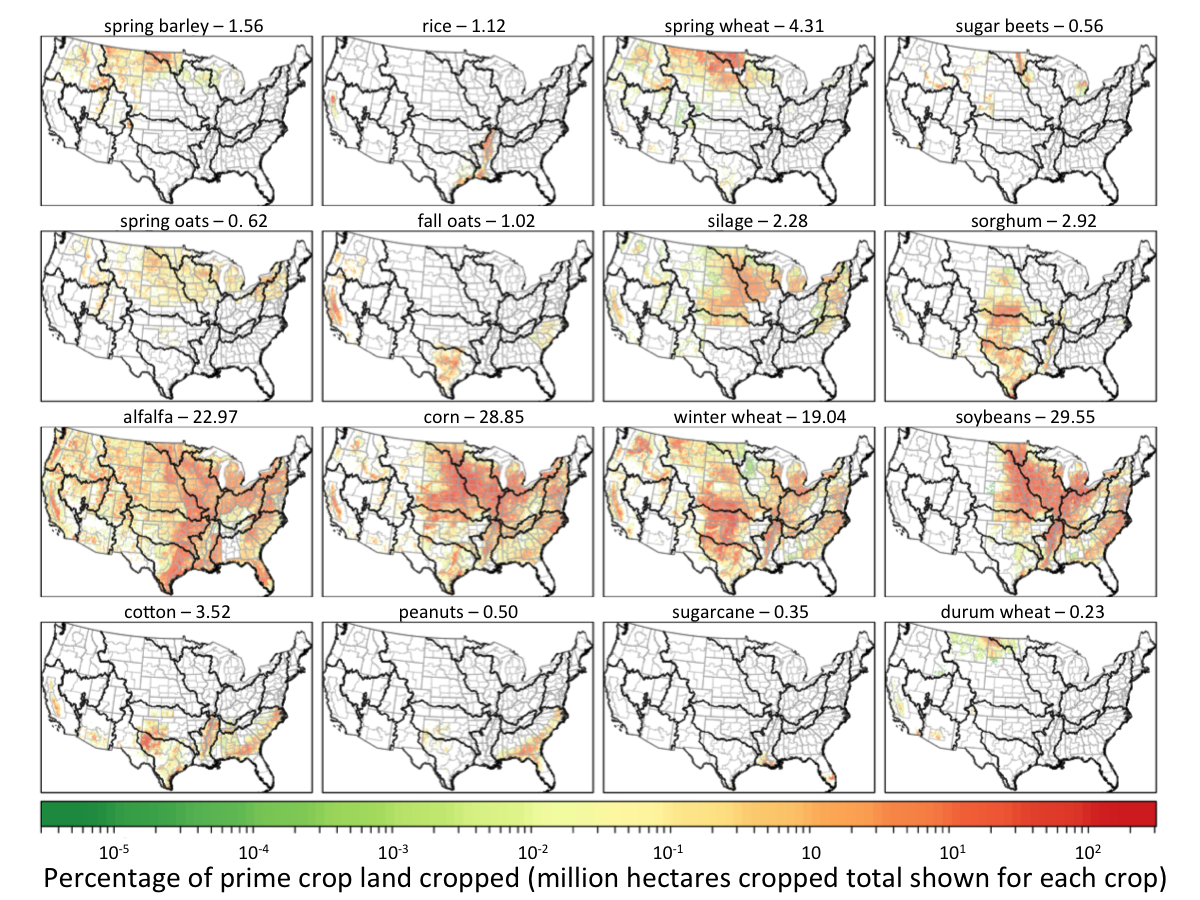

Supplement: S1 File — Table A. Crop and land use categories modeled in BEPAM, CDL, and CropWatR. Table B. Area cropped in million hectares in the base year (2008) and at the end of the modeling period by scenario. Delta values show the percent change in the policy scenario compared with the BAU. Table C. Million hectares irrigated at the end of the modeling period in each scenario. Nationwide changes in irrigated area, by crop, in million hectares. Deltas are the percent difference between the Mandate and CFS scenarios from the counterfactual (no-policy BAU) at the end of the modeling period, respectively. Table D. Literature estimates of blue and green water use for cultivation of biofuels feedstock. Fig A. Base Year (2008) cropping patterns. Colors indicate the percent of land cropped in each 10 x 10 kilometer pixel. Fig B. BAU cropping patterns at the end of the modeling period, in 2030. Fig C. Mandate scenario cropping patterns at the end of the modeling period, in 2030. Fig D. CFS scenario cropping patterns at the end of the modeling period, in 2030. Fig E. Land converted for cellulosic feedstocks in the Mandate scenario. Area cultivated in miscanthus and switchgrass as a percentage of total regular cropland and marginal land, at the resolution of 10 x 10 kilometers at the end of the modeling period. Total land cropped in miscanthus is the sum of land cropped in regular cropland and in marginal land. Switchgrass is cropped only on regular cropland in the Mandate scenario. Fig F. Land converted for cellulosic feedstocks in the CFS scenario. Area cultivated in miscanthus and switchgrass as a percentage of total land, at the resolution of 10 x 10 kilometers at the end of the modeling period. Total land cropped in both miscanthus and switchgrass is the sum of land cropped in regular cropland and in marginal land. Fig G. Land use change (increase or decrease in cropland, at 10 x 10 km resolution) in the Mandate (top) and CFS (bottom) scenarios, relative to the no-policy counterfactual (B [file pone.0204298.s001.zip › SI 1 Figures Tables/S1 Fig B.tiff]

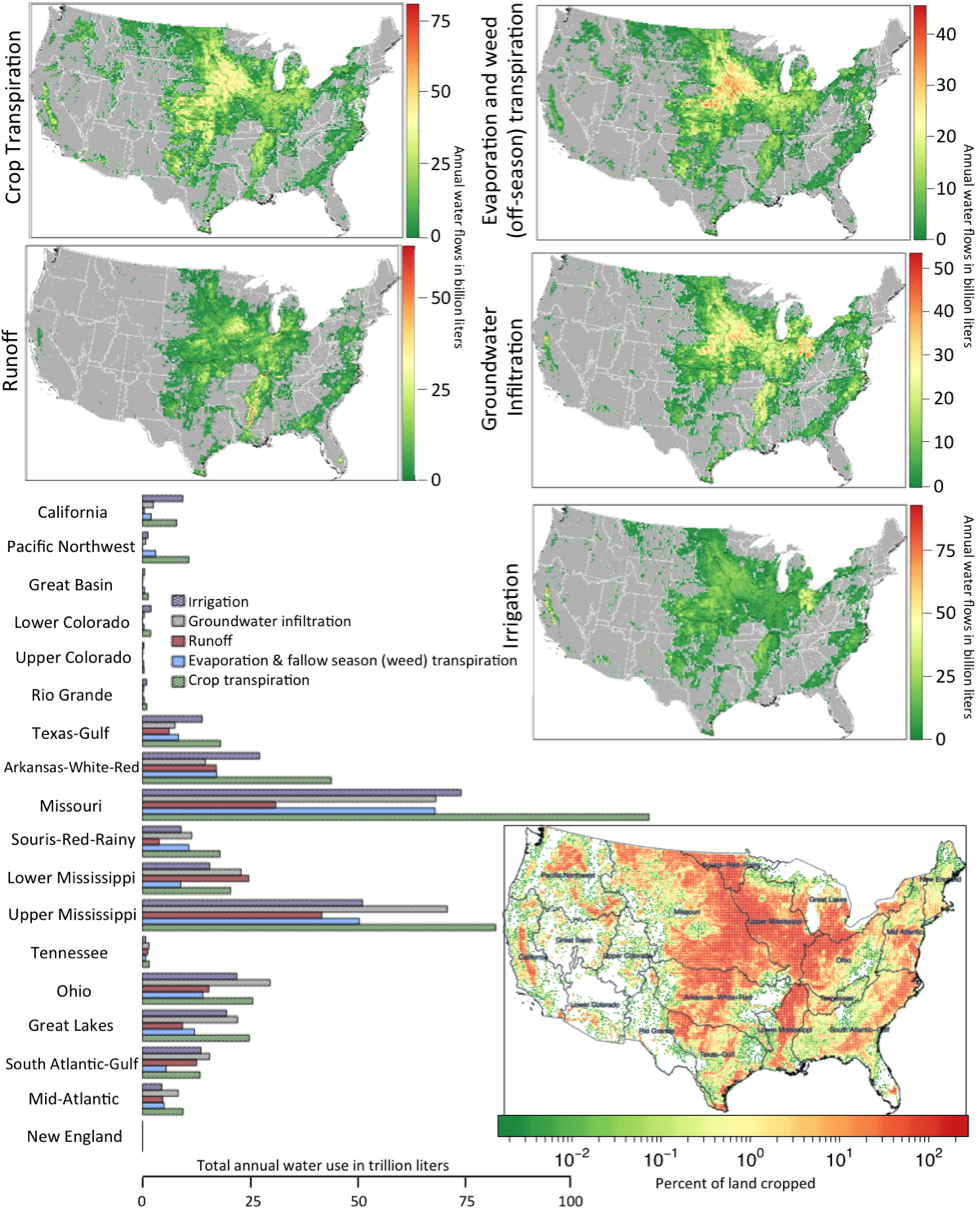

Supplement: S1 File — Table A. Crop and land use categories modeled in BEPAM, CDL, and CropWatR. Table B. Area cropped in million hectares in the base year (2008) and at the end of the modeling period by scenario. Delta values show the percent change in the policy scenario compared with the BAU. Table C. Million hectares irrigated at the end of the modeling period in each scenario. Nationwide changes in irrigated area, by crop, in million hectares. Deltas are the percent difference between the Mandate and CFS scenarios from the counterfactual (no-policy BAU) at the end of the modeling period, respectively. Table D. Literature estimates of blue and green water use for cultivation of biofuels feedstock. Fig A. Base Year (2008) cropping patterns. Colors indicate the percent of land cropped in each 10 x 10 kilometer pixel. Fig B. BAU cropping patterns at the end of the modeling period, in 2030. Fig C. Mandate scenario cropping patterns at the end of the modeling period, in 2030. Fig D. CFS scenario cropping patterns at the end of the modeling period, in 2030. Fig E. Land converted for cellulosic feedstocks in the Mandate scenario. Area cultivated in miscanthus and switchgrass as a percentage of total regular cropland and marginal land, at the resolution of 10 x 10 kilometers at the end of the modeling period. Total land cropped in miscanthus is the sum of land cropped in regular cropland and in marginal land. Switchgrass is cropped only on regular cropland in the Mandate scenario. Fig F. Land converted for cellulosic feedstocks in the CFS scenario. Area cultivated in miscanthus and switchgrass as a percentage of total land, at the resolution of 10 x 10 kilometers at the end of the modeling period. Total land cropped in both miscanthus and switchgrass is the sum of land cropped in regular cropland and in marginal land. Fig G. Land use change (increase or decrease in cropland, at 10 x 10 km resolution) in the Mandate (top) and CFS (bottom) scenarios, relative to the no-policy counterfactual (B [file pone.0204298.s001.zip › SI 1 Figures Tables/S1 Fig I.tiff]

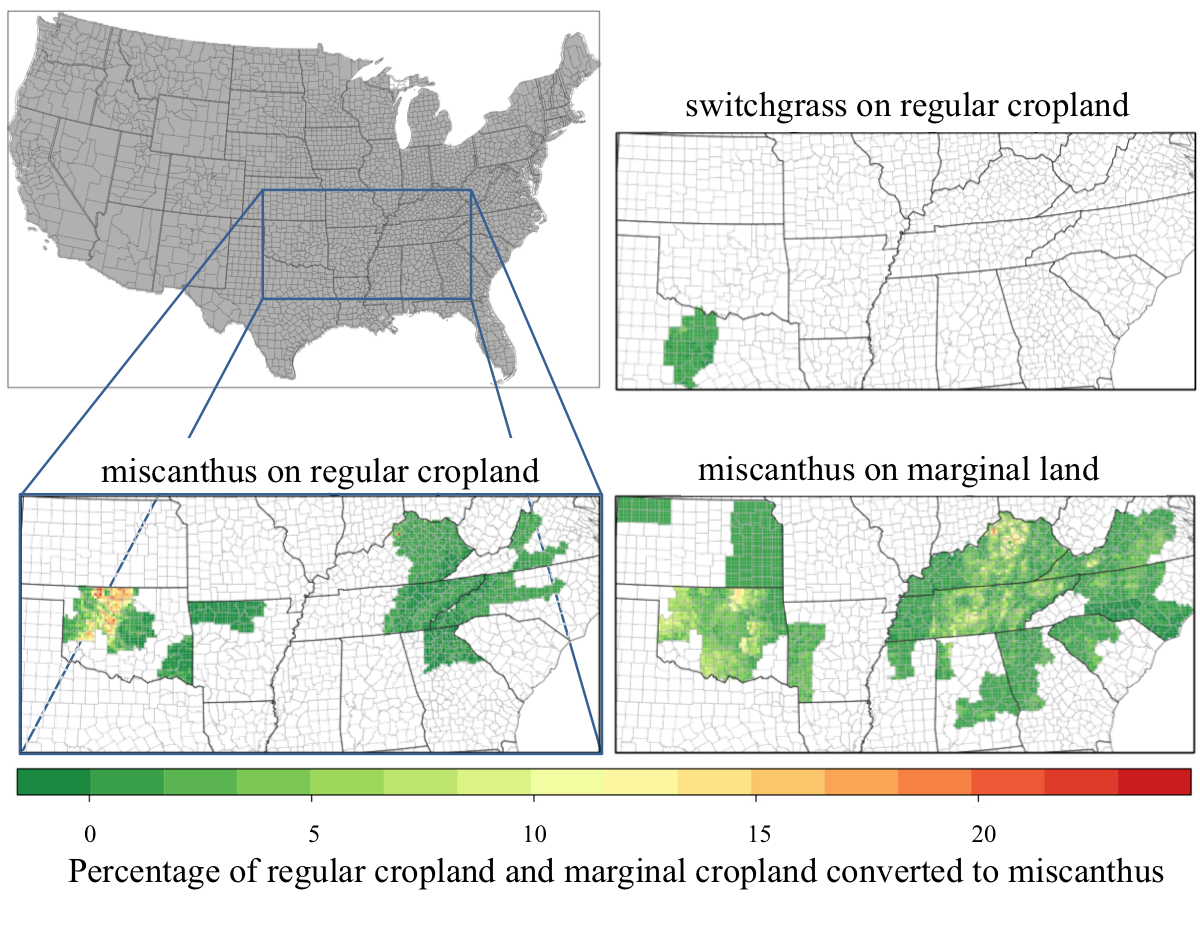

Supplement: S1 File — Table A. Crop and land use categories modeled in BEPAM, CDL, and CropWatR. Table B. Area cropped in million hectares in the base year (2008) and at the end of the modeling period by scenario. Delta values show the percent change in the policy scenario compared with the BAU. Table C. Million hectares irrigated at the end of the modeling period in each scenario. Nationwide changes in irrigated area, by crop, in million hectares. Deltas are the percent difference between the Mandate and CFS scenarios from the counterfactual (no-policy BAU) at the end of the modeling period, respectively. Table D. Literature estimates of blue and green water use for cultivation of biofuels feedstock. Fig A. Base Year (2008) cropping patterns. Colors indicate the percent of land cropped in each 10 x 10 kilometer pixel. Fig B. BAU cropping patterns at the end of the modeling period, in 2030. Fig C. Mandate scenario cropping patterns at the end of the modeling period, in 2030. Fig D. CFS scenario cropping patterns at the end of the modeling period, in 2030. Fig E. Land converted for cellulosic feedstocks in the Mandate scenario. Area cultivated in miscanthus and switchgrass as a percentage of total regular cropland and marginal land, at the resolution of 10 x 10 kilometers at the end of the modeling period. Total land cropped in miscanthus is the sum of land cropped in regular cropland and in marginal land. Switchgrass is cropped only on regular cropland in the Mandate scenario. Fig F. Land converted for cellulosic feedstocks in the CFS scenario. Area cultivated in miscanthus and switchgrass as a percentage of total land, at the resolution of 10 x 10 kilometers at the end of the modeling period. Total land cropped in both miscanthus and switchgrass is the sum of land cropped in regular cropland and in marginal land. Fig G. Land use change (increase or decrease in cropland, at 10 x 10 km resolution) in the Mandate (top) and CFS (bottom) scenarios, relative to the no-policy counterfactual (B [file pone.0204298.s001.zip › SI 1 Figures Tables/S1 Fig E.tiff]

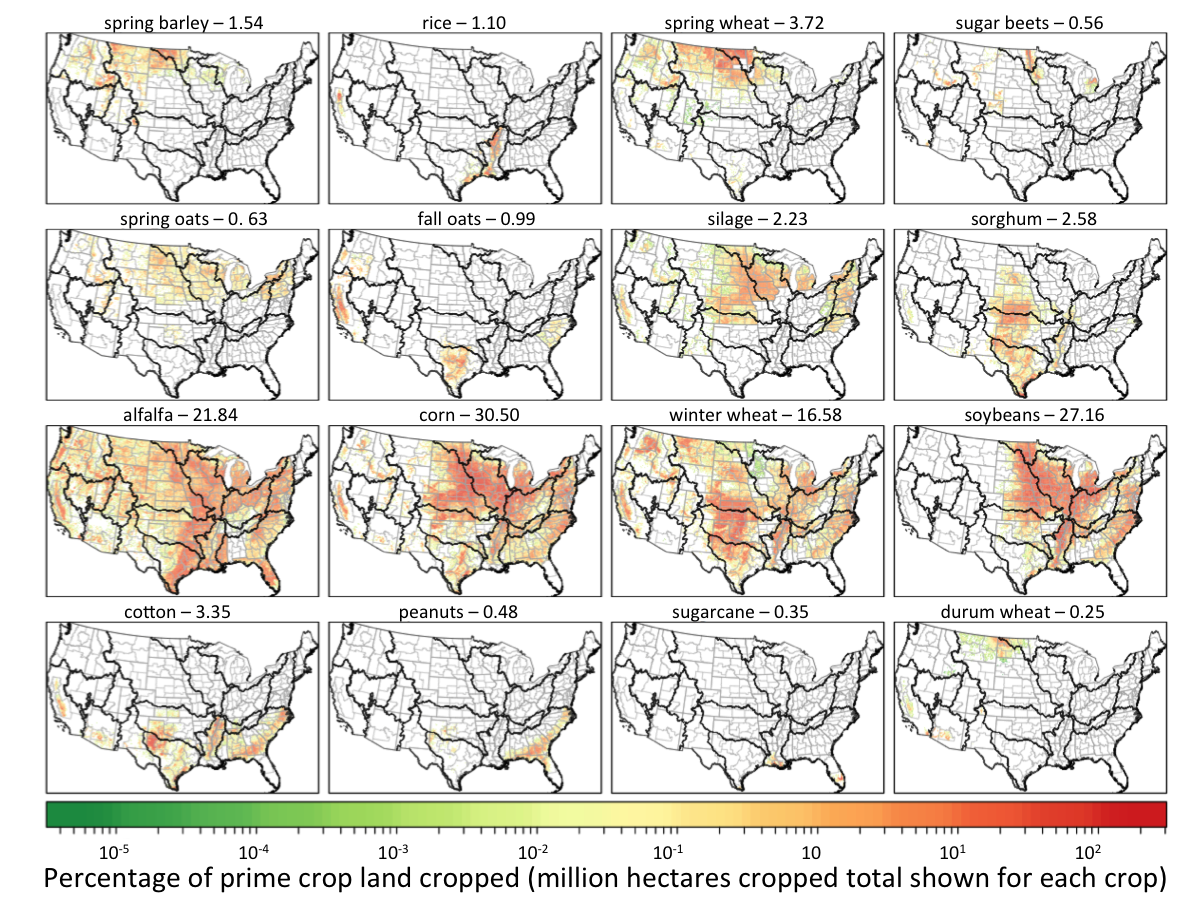

Supplement: S1 File — Table A. Crop and land use categories modeled in BEPAM, CDL, and CropWatR. Table B. Area cropped in million hectares in the base year (2008) and at the end of the modeling period by scenario. Delta values show the percent change in the policy scenario compared with the BAU. Table C. Million hectares irrigated at the end of the modeling period in each scenario. Nationwide changes in irrigated area, by crop, in million hectares. Deltas are the percent difference between the Mandate and CFS scenarios from the counterfactual (no-policy BAU) at the end of the modeling period, respectively. Table D. Literature estimates of blue and green water use for cultivation of biofuels feedstock. Fig A. Base Year (2008) cropping patterns. Colors indicate the percent of land cropped in each 10 x 10 kilometer pixel. Fig B. BAU cropping patterns at the end of the modeling period, in 2030. Fig C. Mandate scenario cropping patterns at the end of the modeling period, in 2030. Fig D. CFS scenario cropping patterns at the end of the modeling period, in 2030. Fig E. Land converted for cellulosic feedstocks in the Mandate scenario. Area cultivated in miscanthus and switchgrass as a percentage of total regular cropland and marginal land, at the resolution of 10 x 10 kilometers at the end of the modeling period. Total land cropped in miscanthus is the sum of land cropped in regular cropland and in marginal land. Switchgrass is cropped only on regular cropland in the Mandate scenario. Fig F. Land converted for cellulosic feedstocks in the CFS scenario. Area cultivated in miscanthus and switchgrass as a percentage of total land, at the resolution of 10 x 10 kilometers at the end of the modeling period. Total land cropped in both miscanthus and switchgrass is the sum of land cropped in regular cropland and in marginal land. Fig G. Land use change (increase or decrease in cropland, at 10 x 10 km resolution) in the Mandate (top) and CFS (bottom) scenarios, relative to the no-policy counterfactual (B [file pone.0204298.s001.zip › SI 1 Figures Tables/S1 Fig D.tiff]

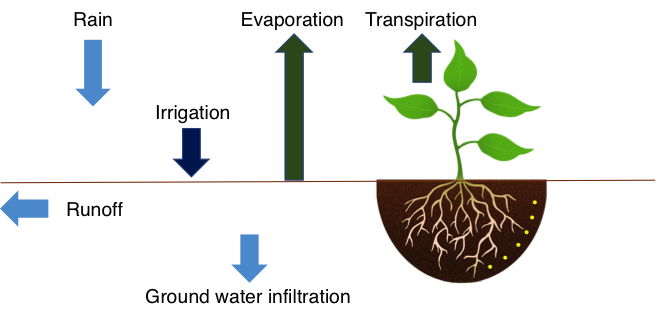

Supplement: S1 File — Table A. Crop and land use categories modeled in BEPAM, CDL, and CropWatR. Table B. Area cropped in million hectares in the base year (2008) and at the end of the modeling period by scenario. Delta values show the percent change in the policy scenario compared with the BAU. Table C. Million hectares irrigated at the end of the modeling period in each scenario. Nationwide changes in irrigated area, by crop, in million hectares. Deltas are the percent difference between the Mandate and CFS scenarios from the counterfactual (no-policy BAU) at the end of the modeling period, respectively. Table D. Literature estimates of blue and green water use for cultivation of biofuels feedstock. Fig A. Base Year (2008) cropping patterns. Colors indicate the percent of land cropped in each 10 x 10 kilometer pixel. Fig B. BAU cropping patterns at the end of the modeling period, in 2030. Fig C. Mandate scenario cropping patterns at the end of the modeling period, in 2030. Fig D. CFS scenario cropping patterns at the end of the modeling period, in 2030. Fig E. Land converted for cellulosic feedstocks in the Mandate scenario. Area cultivated in miscanthus and switchgrass as a percentage of total regular cropland and marginal land, at the resolution of 10 x 10 kilometers at the end of the modeling period. Total land cropped in miscanthus is the sum of land cropped in regular cropland and in marginal land. Switchgrass is cropped only on regular cropland in the Mandate scenario. Fig F. Land converted for cellulosic feedstocks in the CFS scenario. Area cultivated in miscanthus and switchgrass as a percentage of total land, at the resolution of 10 x 10 kilometers at the end of the modeling period. Total land cropped in both miscanthus and switchgrass is the sum of land cropped in regular cropland and in marginal land. Fig G. Land use change (increase or decrease in cropland, at 10 x 10 km resolution) in the Mandate (top) and CFS (bottom) scenarios, relative to the no-policy counterfactual (B [file pone.0204298.s001.zip › SI 1 Figures Tables/S1 Fig H.tiff]

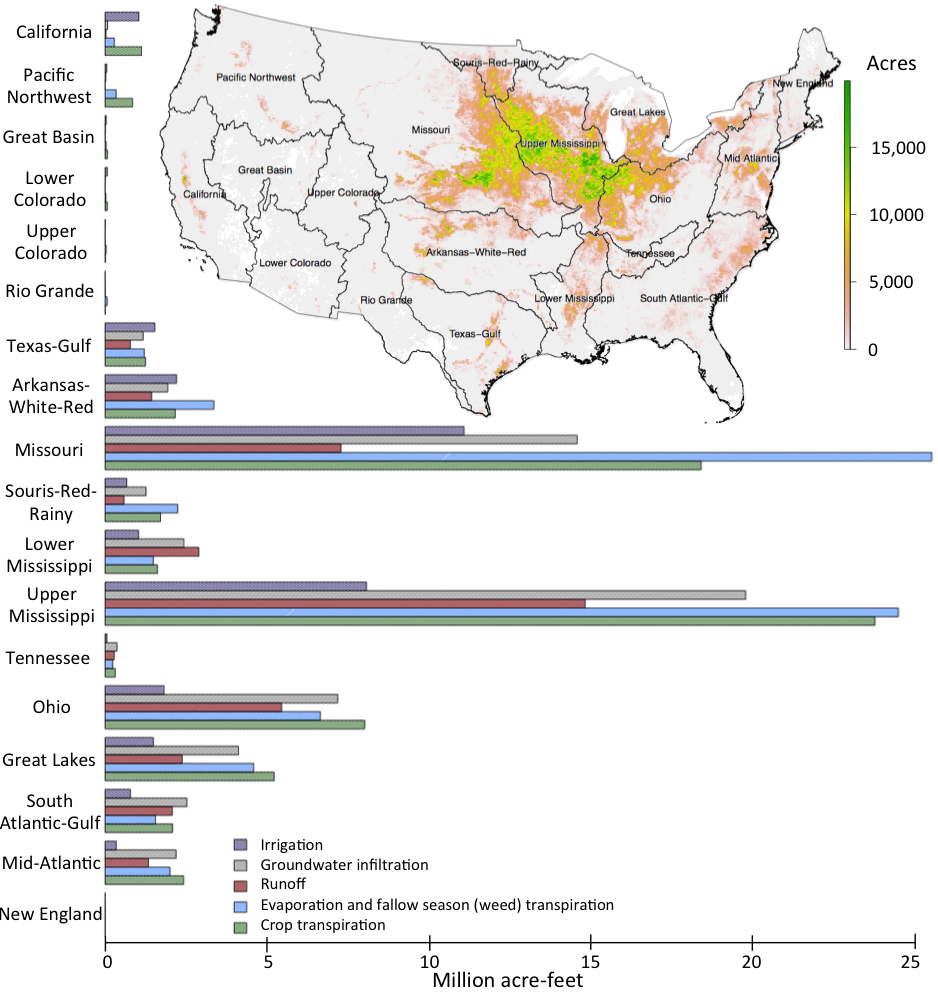

Supplement: S2 File — Table A. Kcb parameter values for perennial grassland and non-crop agricultural land cover types. Table B. Crop water balances that can be estimated for daily, seasonal, or annual time steps. Table C. Literature estimates of evapotranspiration versus modeled evapotranspiration rates. Seasonal and annual evapotranspiration ranges reported in literature sources and model by CropWatR. A range of methods are available for determining evapotranspiration at the field, landscape, and watershed scales, either via direct measurement (e.g. soil moisture measurement via lysimeters), modeling (e.g. via process-based s imulation models, or energy balances using weather data collected via satellite, local instruments, and/or remote sensing). For a summary of common methods, see Connor et al.,2011. Table C. Model performance metrics comparing the results with MODIS 16 estimates. Table E. NASS Classification categories considered in this analysis. Note that double-cropped classifications where both crops were not included among the 14 parameterized crops (e.g. lettuce / upland cotton, lettuce / barley, etc.) were excluded from the analysis. The total acreage of these classifications on the national scale was in all cases much less than 1% of the acreage of the modeled crop. Table F. NASS Accuracy assessments for crops and land types considered in this analysis. Accuracy statistics for crop and land use categories not reported in the above table are not available for 2008. These categories are: alfalfa, other hay, sugarcane, fallow/idle cropland, grassland herbaceous, and pasture/hay. Fig A. Relation between time (day since emergence), crop coefficient (Kcb) and plant height for maize. Crop coefficients are specified for three moments in time. According to the FAO 56 methods, coefficients are estimated by stepwise and linear interpolation (black line). In CropWatR, a Bezier curve (red curve) is used to interpolate daily Kcb values. Fig B. Irrigation calibration results for corn. The [file pone.0204298.s002.zip › S2 File Figures Tables/S2 Fig D.tiff]

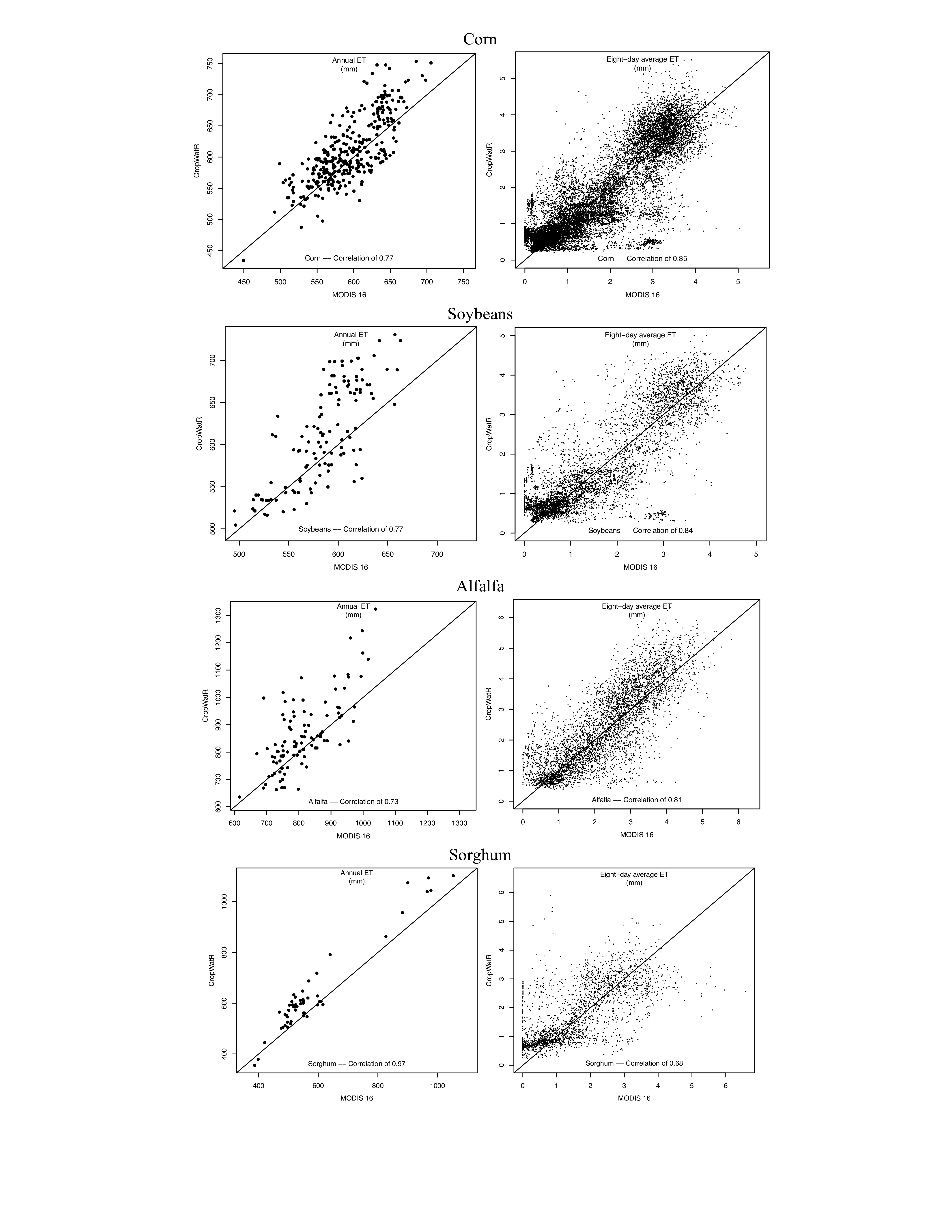

Supplement: S2 File — Table A. Kcb parameter values for perennial grassland and non-crop agricultural land cover types. Table B. Crop water balances that can be estimated for daily, seasonal, or annual time steps. Table C. Literature estimates of evapotranspiration versus modeled evapotranspiration rates. Seasonal and annual evapotranspiration ranges reported in literature sources and model by CropWatR. A range of methods are available for determining evapotranspiration at the field, landscape, and watershed scales, either via direct measurement (e.g. soil moisture measurement via lysimeters), modeling (e.g. via process-based s imulation models, or energy balances using weather data collected via satellite, local instruments, and/or remote sensing). For a summary of common methods, see Connor et al.,2011. Table C. Model performance metrics comparing the results with MODIS 16 estimates. Table E. NASS Classification categories considered in this analysis. Note that double-cropped classifications where both crops were not included among the 14 parameterized crops (e.g. lettuce / upland cotton, lettuce / barley, etc.) were excluded from the analysis. The total acreage of these classifications on the national scale was in all cases much less than 1% of the acreage of the modeled crop. Table F. NASS Accuracy assessments for crops and land types considered in this analysis. Accuracy statistics for crop and land use categories not reported in the above table are not available for 2008. These categories are: alfalfa, other hay, sugarcane, fallow/idle cropland, grassland herbaceous, and pasture/hay. Fig A. Relation between time (day since emergence), crop coefficient (Kcb) and plant height for maize. Crop coefficients are specified for three moments in time. According to the FAO 56 methods, coefficients are estimated by stepwise and linear interpolation (black line). In CropWatR, a Bezier curve (red curve) is used to interpolate daily Kcb values. Fig B. Irrigation calibration results for corn. The [file pone.0204298.s002.zip › S2 File Figures Tables/S2 Fig H.tiff]

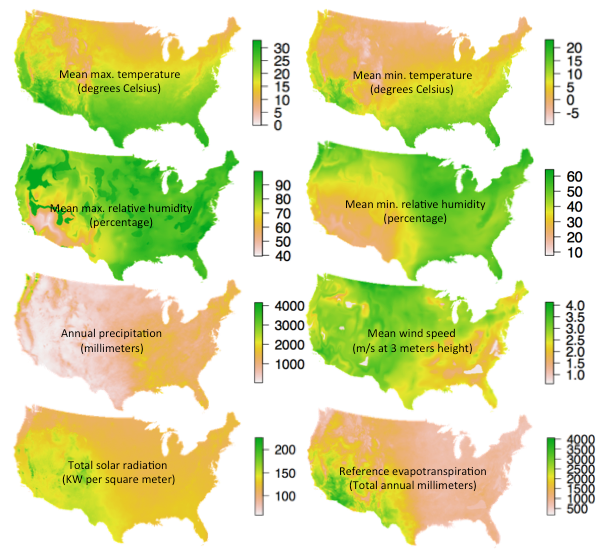

Supplement: S2 File — Table A. Kcb parameter values for perennial grassland and non-crop agricultural land cover types. Table B. Crop water balances that can be estimated for daily, seasonal, or annual time steps. Table C. Literature estimates of evapotranspiration versus modeled evapotranspiration rates. Seasonal and annual evapotranspiration ranges reported in literature sources and model by CropWatR. A range of methods are available for determining evapotranspiration at the field, landscape, and watershed scales, either via direct measurement (e.g. soil moisture measurement via lysimeters), modeling (e.g. via process-based s imulation models, or energy balances using weather data collected via satellite, local instruments, and/or remote sensing). For a summary of common methods, see Connor et al.,2011. Table C. Model performance metrics comparing the results with MODIS 16 estimates. Table E. NASS Classification categories considered in this analysis. Note that double-cropped classifications where both crops were not included among the 14 parameterized crops (e.g. lettuce / upland cotton, lettuce / barley, etc.) were excluded from the analysis. The total acreage of these classifications on the national scale was in all cases much less than 1% of the acreage of the modeled crop. Table F. NASS Accuracy assessments for crops and land types considered in this analysis. Accuracy statistics for crop and land use categories not reported in the above table are not available for 2008. These categories are: alfalfa, other hay, sugarcane, fallow/idle cropland, grassland herbaceous, and pasture/hay. Fig A. Relation between time (day since emergence), crop coefficient (Kcb) and plant height for maize. Crop coefficients are specified for three moments in time. According to the FAO 56 methods, coefficients are estimated by stepwise and linear interpolation (black line). In CropWatR, a Bezier curve (red curve) is used to interpolate daily Kcb values. Fig B. Irrigation calibration results for corn. The [file pone.0204298.s002.zip › S2 File Figures Tables/S2 Fig I.tiff]

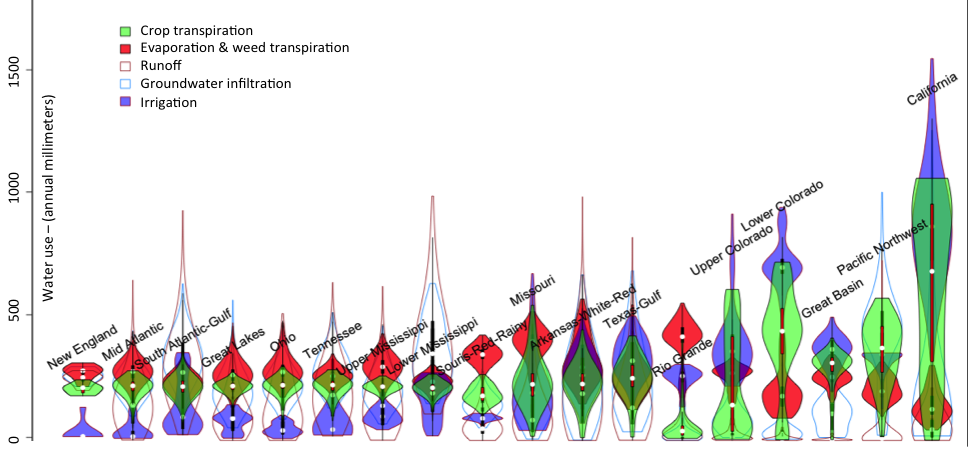

Supplement: S2 File — Table A. Kcb parameter values for perennial grassland and non-crop agricultural land cover types. Table B. Crop water balances that can be estimated for daily, seasonal, or annual time steps. Table C. Literature estimates of evapotranspiration versus modeled evapotranspiration rates. Seasonal and annual evapotranspiration ranges reported in literature sources and model by CropWatR. A range of methods are available for determining evapotranspiration at the field, landscape, and watershed scales, either via direct measurement (e.g. soil moisture measurement via lysimeters), modeling (e.g. via process-based s imulation models, or energy balances using weather data collected via satellite, local instruments, and/or remote sensing). For a summary of common methods, see Connor et al.,2011. Table C. Model performance metrics comparing the results with MODIS 16 estimates. Table E. NASS Classification categories considered in this analysis. Note that double-cropped classifications where both crops were not included among the 14 parameterized crops (e.g. lettuce / upland cotton, lettuce / barley, etc.) were excluded from the analysis. The total acreage of these classifications on the national scale was in all cases much less than 1% of the acreage of the modeled crop. Table F. NASS Accuracy assessments for crops and land types considered in this analysis. Accuracy statistics for crop and land use categories not reported in the above table are not available for 2008. These categories are: alfalfa, other hay, sugarcane, fallow/idle cropland, grassland herbaceous, and pasture/hay. Fig A. Relation between time (day since emergence), crop coefficient (Kcb) and plant height for maize. Crop coefficients are specified for three moments in time. According to the FAO 56 methods, coefficients are estimated by stepwise and linear interpolation (black line). In CropWatR, a Bezier curve (red curve) is used to interpolate daily Kcb values. Fig B. Irrigation calibration results for corn. The [file pone.0204298.s002.zip › S2 File Figures Tables/S2 Fig E.tiff]

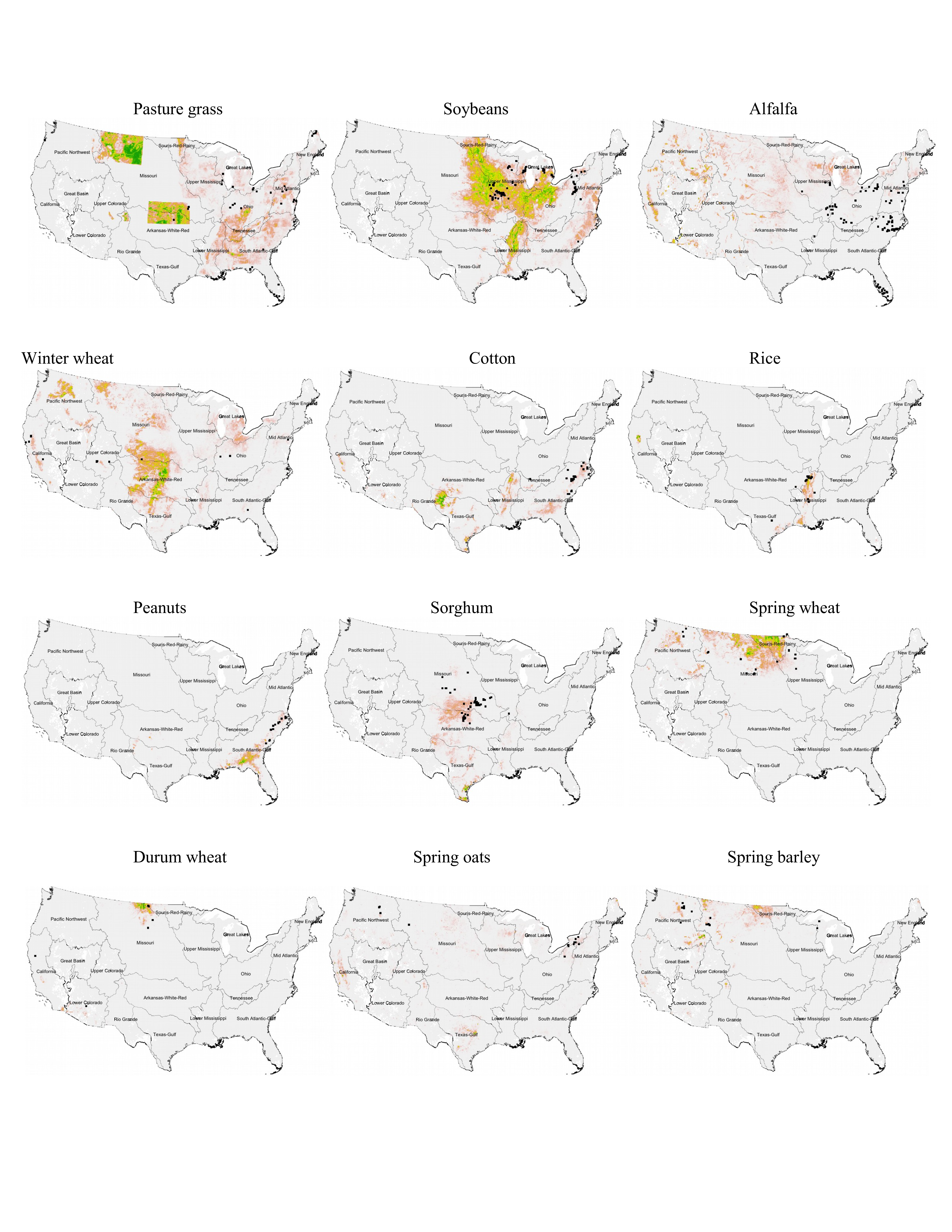

Supplement: S2 File — Table A. Kcb parameter values for perennial grassland and non-crop agricultural land cover types. Table B. Crop water balances that can be estimated for daily, seasonal, or annual time steps. Table C. Literature estimates of evapotranspiration versus modeled evapotranspiration rates. Seasonal and annual evapotranspiration ranges reported in literature sources and model by CropWatR. A range of methods are available for determining evapotranspiration at the field, landscape, and watershed scales, either via direct measurement (e.g. soil moisture measurement via lysimeters), modeling (e.g. via process-based s imulation models, or energy balances using weather data collected via satellite, local instruments, and/or remote sensing). For a summary of common methods, see Connor et al.,2011. Table C. Model performance metrics comparing the results with MODIS 16 estimates. Table E. NASS Classification categories considered in this analysis. Note that double-cropped classifications where both crops were not included among the 14 parameterized crops (e.g. lettuce / upland cotton, lettuce / barley, etc.) were excluded from the analysis. The total acreage of these classifications on the national scale was in all cases much less than 1% of the acreage of the modeled crop. Table F. NASS Accuracy assessments for crops and land types considered in this analysis. Accuracy statistics for crop and land use categories not reported in the above table are not available for 2008. These categories are: alfalfa, other hay, sugarcane, fallow/idle cropland, grassland herbaceous, and pasture/hay. Fig A. Relation between time (day since emergence), crop coefficient (Kcb) and plant height for maize. Crop coefficients are specified for three moments in time. According to the FAO 56 methods, coefficients are estimated by stepwise and linear interpolation (black line). In CropWatR, a Bezier curve (red curve) is used to interpolate daily Kcb values. Fig B. Irrigation calibration results for corn. The [file pone.0204298.s002.zip › S2 File Figures Tables/S2 Fig N.tiff]

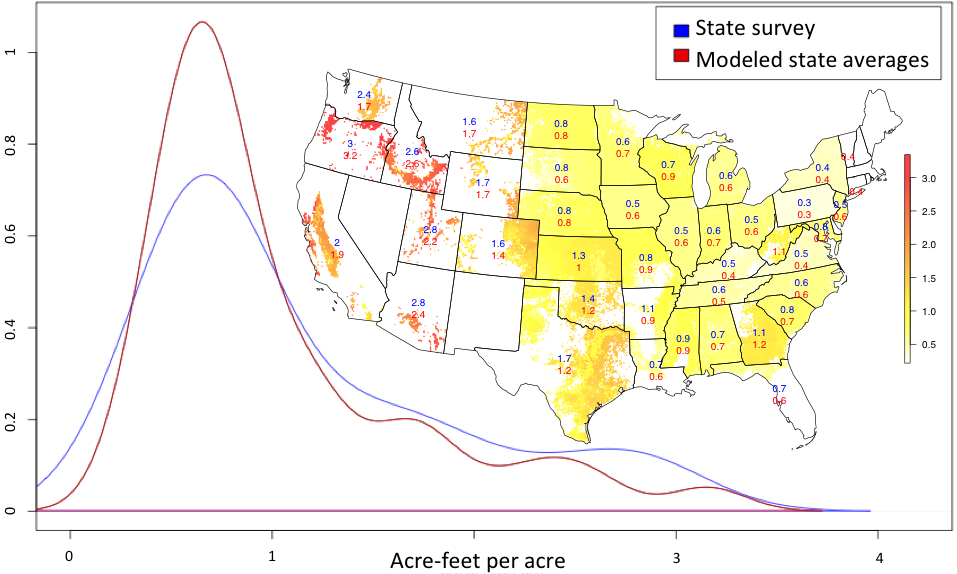

Supplement: S2 File — Table A. Kcb parameter values for perennial grassland and non-crop agricultural land cover types. Table B. Crop water balances that can be estimated for daily, seasonal, or annual time steps. Table C. Literature estimates of evapotranspiration versus modeled evapotranspiration rates. Seasonal and annual evapotranspiration ranges reported in literature sources and model by CropWatR. A range of methods are available for determining evapotranspiration at the field, landscape, and watershed scales, either via direct measurement (e.g. soil moisture measurement via lysimeters), modeling (e.g. via process-based s imulation models, or energy balances using weather data collected via satellite, local instruments, and/or remote sensing). For a summary of common methods, see Connor et al.,2011. Table C. Model performance metrics comparing the results with MODIS 16 estimates. Table E. NASS Classification categories considered in this analysis. Note that double-cropped classifications where both crops were not included among the 14 parameterized crops (e.g. lettuce / upland cotton, lettuce / barley, etc.) were excluded from the analysis. The total acreage of these classifications on the national scale was in all cases much less than 1% of the acreage of the modeled crop. Table F. NASS Accuracy assessments for crops and land types considered in this analysis. Accuracy statistics for crop and land use categories not reported in the above table are not available for 2008. These categories are: alfalfa, other hay, sugarcane, fallow/idle cropland, grassland herbaceous, and pasture/hay. Fig A. Relation between time (day since emergence), crop coefficient (Kcb) and plant height for maize. Crop coefficients are specified for three moments in time. According to the FAO 56 methods, coefficients are estimated by stepwise and linear interpolation (black line). In CropWatR, a Bezier curve (red curve) is used to interpolate daily Kcb values. Fig B. Irrigation calibration results for corn. The [file pone.0204298.s002.zip › S2 File Figures Tables/S2 Fig B.tiff]

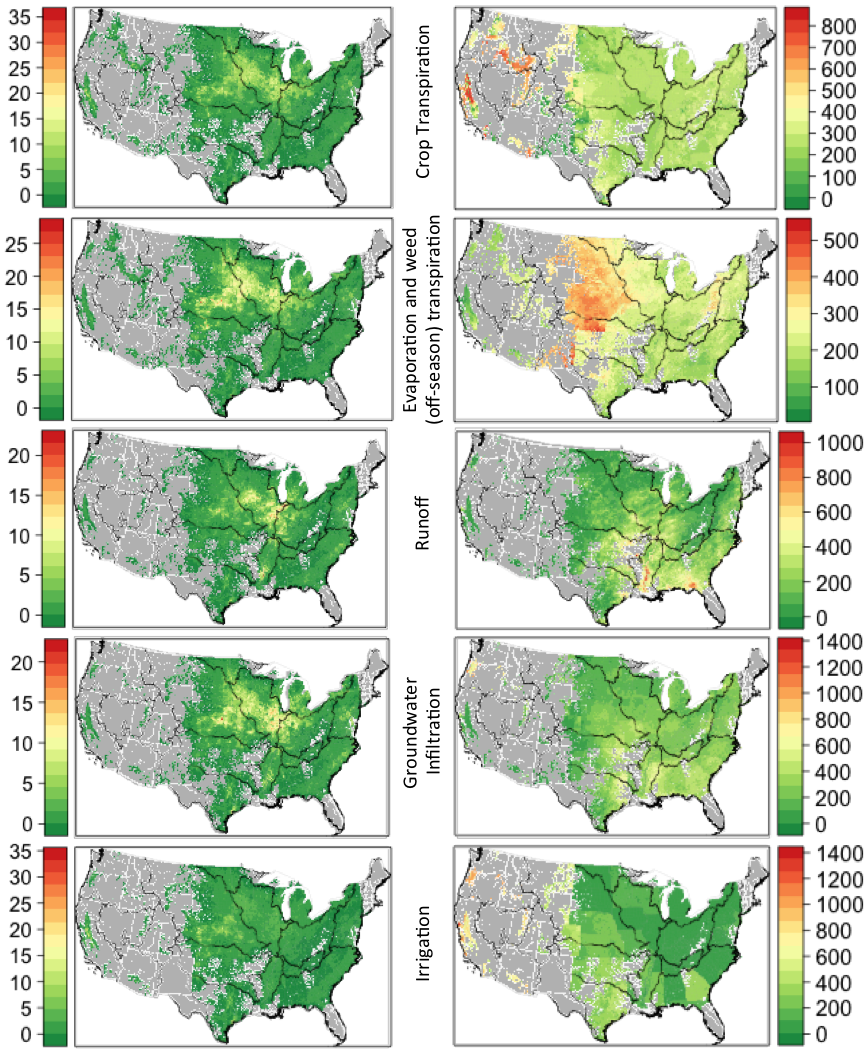

Supplement: S2 File — Table A. Kcb parameter values for perennial grassland and non-crop agricultural land cover types. Table B. Crop water balances that can be estimated for daily, seasonal, or annual time steps. Table C. Literature estimates of evapotranspiration versus modeled evapotranspiration rates. Seasonal and annual evapotranspiration ranges reported in literature sources and model by CropWatR. A range of methods are available for determining evapotranspiration at the field, landscape, and watershed scales, either via direct measurement (e.g. soil moisture measurement via lysimeters), modeling (e.g. via process-based s imulation models, or energy balances using weather data collected via satellite, local instruments, and/or remote sensing). For a summary of common methods, see Connor et al.,2011. Table C. Model performance metrics comparing the results with MODIS 16 estimates. Table E. NASS Classification categories considered in this analysis. Note that double-cropped classifications where both crops were not included among the 14 parameterized crops (e.g. lettuce / upland cotton, lettuce / barley, etc.) were excluded from the analysis. The total acreage of these classifications on the national scale was in all cases much less than 1% of the acreage of the modeled crop. Table F. NASS Accuracy assessments for crops and land types considered in this analysis. Accuracy statistics for crop and land use categories not reported in the above table are not available for 2008. These categories are: alfalfa, other hay, sugarcane, fallow/idle cropland, grassland herbaceous, and pasture/hay. Fig A. Relation between time (day since emergence), crop coefficient (Kcb) and plant height for maize. Crop coefficients are specified for three moments in time. According to the FAO 56 methods, coefficients are estimated by stepwise and linear interpolation (black line). In CropWatR, a Bezier curve (red curve) is used to interpolate daily Kcb values. Fig B. Irrigation calibration results for corn. The [file pone.0204298.s002.zip › S2 File Figures Tables/S2 Fig C.tiff]

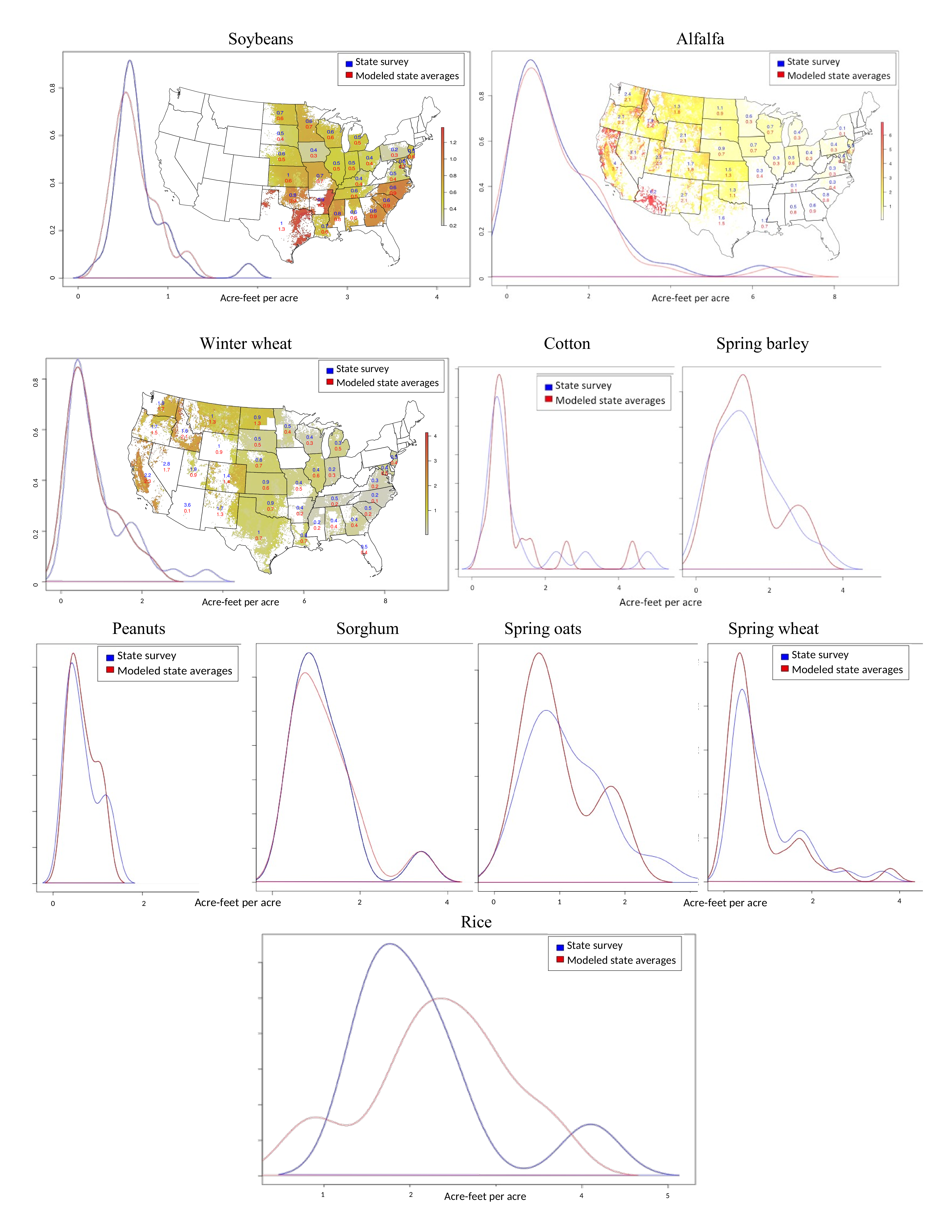

Supplement: S2 File — Table A. Kcb parameter values for perennial grassland and non-crop agricultural land cover types. Table B. Crop water balances that can be estimated for daily, seasonal, or annual time steps. Table C. Literature estimates of evapotranspiration versus modeled evapotranspiration rates. Seasonal and annual evapotranspiration ranges reported in literature sources and model by CropWatR. A range of methods are available for determining evapotranspiration at the field, landscape, and watershed scales, either via direct measurement (e.g. soil moisture measurement via lysimeters), modeling (e.g. via process-based s imulation models, or energy balances using weather data collected via satellite, local instruments, and/or remote sensing). For a summary of common methods, see Connor et al.,2011. Table C. Model performance metrics comparing the results with MODIS 16 estimates. Table E. NASS Classification categories considered in this analysis. Note that double-cropped classifications where both crops were not included among the 14 parameterized crops (e.g. lettuce / upland cotton, lettuce / barley, etc.) were excluded from the analysis. The total acreage of these classifications on the national scale was in all cases much less than 1% of the acreage of the modeled crop. Table F. NASS Accuracy assessments for crops and land types considered in this analysis. Accuracy statistics for crop and land use categories not reported in the above table are not available for 2008. These categories are: alfalfa, other hay, sugarcane, fallow/idle cropland, grassland herbaceous, and pasture/hay. Fig A. Relation between time (day since emergence), crop coefficient (Kcb) and plant height for maize. Crop coefficients are specified for three moments in time. According to the FAO 56 methods, coefficients are estimated by stepwise and linear interpolation (black line). In CropWatR, a Bezier curve (red curve) is used to interpolate daily Kcb values. Fig B. Irrigation calibration results for corn. The [file pone.0204298.s002.zip › S2 File Figures Tables/S2 Fig L.tiff]

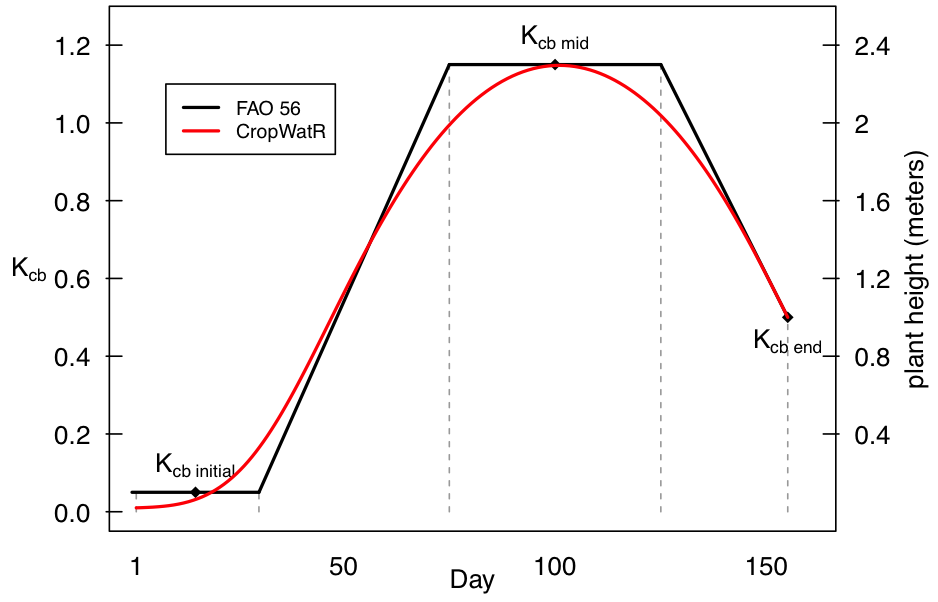

Supplement: S2 File — Table A. Kcb parameter values for perennial grassland and non-crop agricultural land cover types. Table B. Crop water balances that can be estimated for daily, seasonal, or annual time steps. Table C. Literature estimates of evapotranspiration versus modeled evapotranspiration rates. Seasonal and annual evapotranspiration ranges reported in literature sources and model by CropWatR. A range of methods are available for determining evapotranspiration at the field, landscape, and watershed scales, either via direct measurement (e.g. soil moisture measurement via lysimeters), modeling (e.g. via process-based s imulation models, or energy balances using weather data collected via satellite, local instruments, and/or remote sensing). For a summary of common methods, see Connor et al.,2011. Table C. Model performance metrics comparing the results with MODIS 16 estimates. Table E. NASS Classification categories considered in this analysis. Note that double-cropped classifications where both crops were not included among the 14 parameterized crops (e.g. lettuce / upland cotton, lettuce / barley, etc.) were excluded from the analysis. The total acreage of these classifications on the national scale was in all cases much less than 1% of the acreage of the modeled crop. Table F. NASS Accuracy assessments for crops and land types considered in this analysis. Accuracy statistics for crop and land use categories not reported in the above table are not available for 2008. These categories are: alfalfa, other hay, sugarcane, fallow/idle cropland, grassland herbaceous, and pasture/hay. Fig A. Relation between time (day since emergence), crop coefficient (Kcb) and plant height for maize. Crop coefficients are specified for three moments in time. According to the FAO 56 methods, coefficients are estimated by stepwise and linear interpolation (black line). In CropWatR, a Bezier curve (red curve) is used to interpolate daily Kcb values. Fig B. Irrigation calibration results for corn. The [file pone.0204298.s002.zip › S2 File Figures Tables/S2 Fig A.tiff]

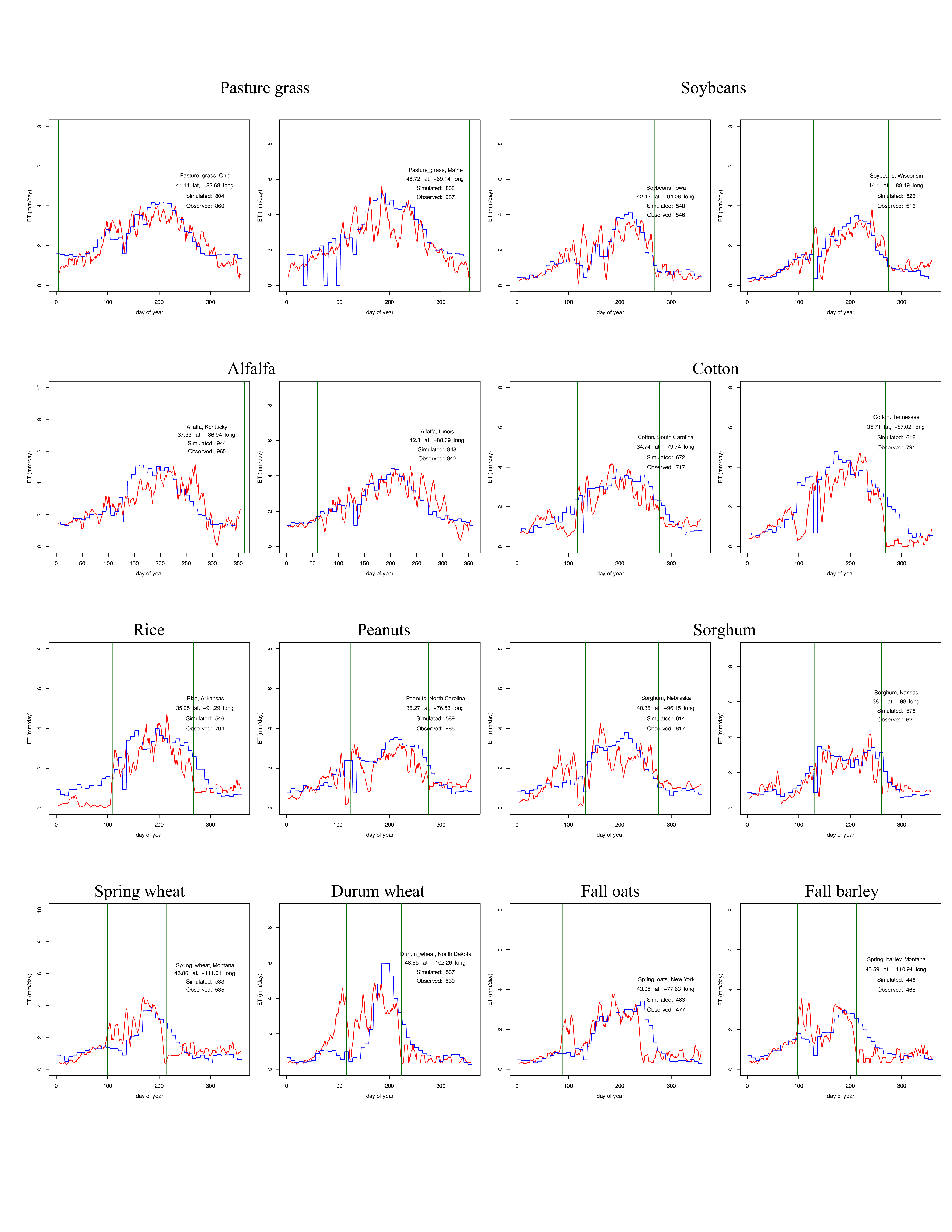

Supplement: S2 File — Table A. Kcb parameter values for perennial grassland and non-crop agricultural land cover types. Table B. Crop water balances that can be estimated for daily, seasonal, or annual time steps. Table C. Literature estimates of evapotranspiration versus modeled evapotranspiration rates. Seasonal and annual evapotranspiration ranges reported in literature sources and model by CropWatR. A range of methods are available for determining evapotranspiration at the field, landscape, and watershed scales, either via direct measurement (e.g. soil moisture measurement via lysimeters), modeling (e.g. via process-based s imulation models, or energy balances using weather data collected via satellite, local instruments, and/or remote sensing). For a summary of common methods, see Connor et al.,2011. Table C. Model performance metrics comparing the results with MODIS 16 estimates. Table E. NASS Classification categories considered in this analysis. Note that double-cropped classifications where both crops were not included among the 14 parameterized crops (e.g. lettuce / upland cotton, lettuce / barley, etc.) were excluded from the analysis. The total acreage of these classifications on the national scale was in all cases much less than 1% of the acreage of the modeled crop. Table F. NASS Accuracy assessments for crops and land types considered in this analysis. Accuracy statistics for crop and land use categories not reported in the above table are not available for 2008. These categories are: alfalfa, other hay, sugarcane, fallow/idle cropland, grassland herbaceous, and pasture/hay. Fig A. Relation between time (day since emergence), crop coefficient (Kcb) and plant height for maize. Crop coefficients are specified for three moments in time. According to the FAO 56 methods, coefficients are estimated by stepwise and linear interpolation (black line). In CropWatR, a Bezier curve (red curve) is used to interpolate daily Kcb values. Fig B. Irrigation calibration results for corn. The [file pone.0204298.s002.zip › S2 File Figures Tables/S2 Fig M.tiff]

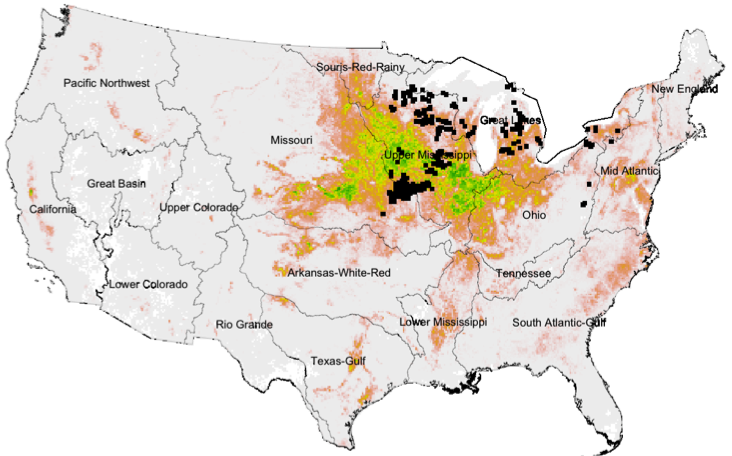

Supplement: S2 File — Table A. Kcb parameter values for perennial grassland and non-crop agricultural land cover types. Table B. Crop water balances that can be estimated for daily, seasonal, or annual time steps. Table C. Literature estimates of evapotranspiration versus modeled evapotranspiration rates. Seasonal and annual evapotranspiration ranges reported in literature sources and model by CropWatR. A range of methods are available for determining evapotranspiration at the field, landscape, and watershed scales, either via direct measurement (e.g. soil moisture measurement via lysimeters), modeling (e.g. via process-based s imulation models, or energy balances using weather data collected via satellite, local instruments, and/or remote sensing). For a summary of common methods, see Connor et al.,2011. Table C. Model performance metrics comparing the results with MODIS 16 estimates. Table E. NASS Classification categories considered in this analysis. Note that double-cropped classifications where both crops were not included among the 14 parameterized crops (e.g. lettuce / upland cotton, lettuce / barley, etc.) were excluded from the analysis. The total acreage of these classifications on the national scale was in all cases much less than 1% of the acreage of the modeled crop. Table F. NASS Accuracy assessments for crops and land types considered in this analysis. Accuracy statistics for crop and land use categories not reported in the above table are not available for 2008. These categories are: alfalfa, other hay, sugarcane, fallow/idle cropland, grassland herbaceous, and pasture/hay. Fig A. Relation between time (day since emergence), crop coefficient (Kcb) and plant height for maize. Crop coefficients are specified for three moments in time. According to the FAO 56 methods, coefficients are estimated by stepwise and linear interpolation (black line). In CropWatR, a Bezier curve (red curve) is used to interpolate daily Kcb values. Fig B. Irrigation calibration results for corn. The [file pone.0204298.s002.zip › S2 File Figures Tables/S2 Fig F.tiff]

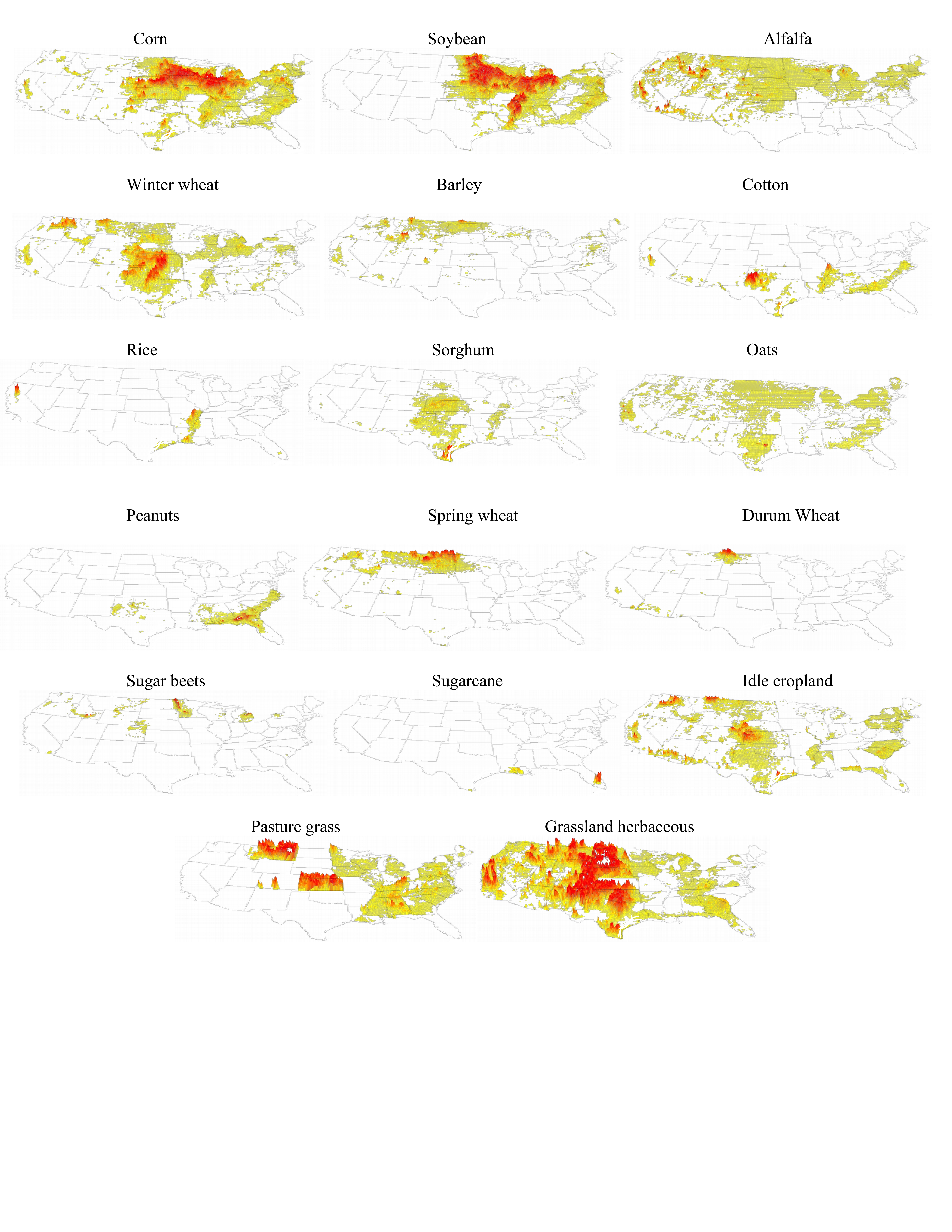

Supplement: S2 File — Table A. Kcb parameter values for perennial grassland and non-crop agricultural land cover types. Table B. Crop water balances that can be estimated for daily, seasonal, or annual time steps. Table C. Literature estimates of evapotranspiration versus modeled evapotranspiration rates. Seasonal and annual evapotranspiration ranges reported in literature sources and model by CropWatR. A range of methods are available for determining evapotranspiration at the field, landscape, and watershed scales, either via direct measurement (e.g. soil moisture measurement via lysimeters), modeling (e.g. via process-based s imulation models, or energy balances using weather data collected via satellite, local instruments, and/or remote sensing). For a summary of common methods, see Connor et al.,2011. Table C. Model performance metrics comparing the results with MODIS 16 estimates. Table E. NASS Classification categories considered in this analysis. Note that double-cropped classifications where both crops were not included among the 14 parameterized crops (e.g. lettuce / upland cotton, lettuce / barley, etc.) were excluded from the analysis. The total acreage of these classifications on the national scale was in all cases much less than 1% of the acreage of the modeled crop. Table F. NASS Accuracy assessments for crops and land types considered in this analysis. Accuracy statistics for crop and land use categories not reported in the above table are not available for 2008. These categories are: alfalfa, other hay, sugarcane, fallow/idle cropland, grassland herbaceous, and pasture/hay. Fig A. Relation between time (day since emergence), crop coefficient (Kcb) and plant height for maize. Crop coefficients are specified for three moments in time. According to the FAO 56 methods, coefficients are estimated by stepwise and linear interpolation (black line). In CropWatR, a Bezier curve (red curve) is used to interpolate daily Kcb values. Fig B. Irrigation calibration results for corn. The [file pone.0204298.s002.zip › S2 File Figures Tables/S2 Fig J.tiff]

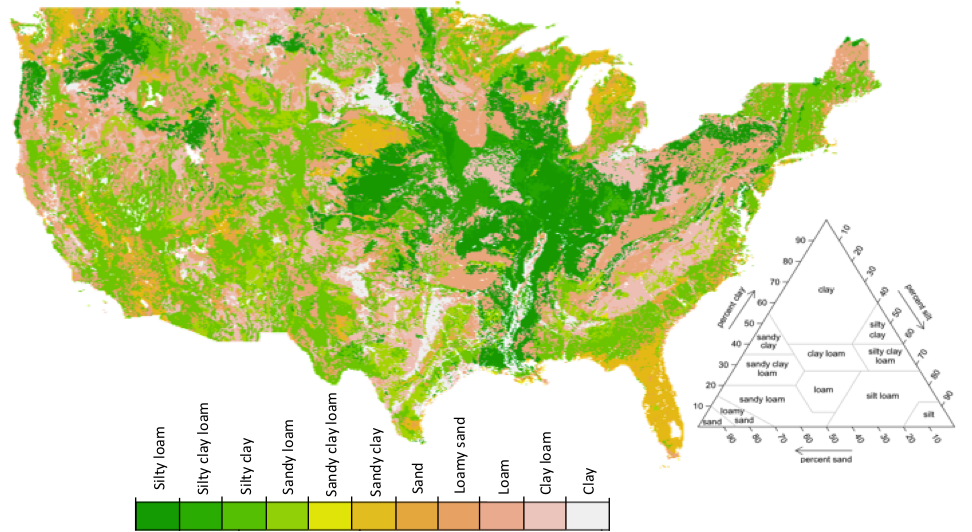

Supplement: S2 File — Table A. Kcb parameter values for perennial grassland and non-crop agricultural land cover types. Table B. Crop water balances that can be estimated for daily, seasonal, or annual time steps. Table C. Literature estimates of evapotranspiration versus modeled evapotranspiration rates. Seasonal and annual evapotranspiration ranges reported in literature sources and model by CropWatR. A range of methods are available for determining evapotranspiration at the field, landscape, and watershed scales, either via direct measurement (e.g. soil moisture measurement via lysimeters), modeling (e.g. via process-based s imulation models, or energy balances using weather data collected via satellite, local instruments, and/or remote sensing). For a summary of common methods, see Connor et al.,2011. Table C. Model performance metrics comparing the results with MODIS 16 estimates. Table E. NASS Classification categories considered in this analysis. Note that double-cropped classifications where both crops were not included among the 14 parameterized crops (e.g. lettuce / upland cotton, lettuce / barley, etc.) were excluded from the analysis. The total acreage of these classifications on the national scale was in all cases much less than 1% of the acreage of the modeled crop. Table F. NASS Accuracy assessments for crops and land types considered in this analysis. Accuracy statistics for crop and land use categories not reported in the above table are not available for 2008. These categories are: alfalfa, other hay, sugarcane, fallow/idle cropland, grassland herbaceous, and pasture/hay. Fig A. Relation between time (day since emergence), crop coefficient (Kcb) and plant height for maize. Crop coefficients are specified for three moments in time. According to the FAO 56 methods, coefficients are estimated by stepwise and linear interpolation (black line). In CropWatR, a Bezier curve (red curve) is used to interpolate daily Kcb values. Fig B. Irrigation calibration results for corn. The [file pone.0204298.s002.zip › S2 File Figures Tables/S2 Fig K.tiff]

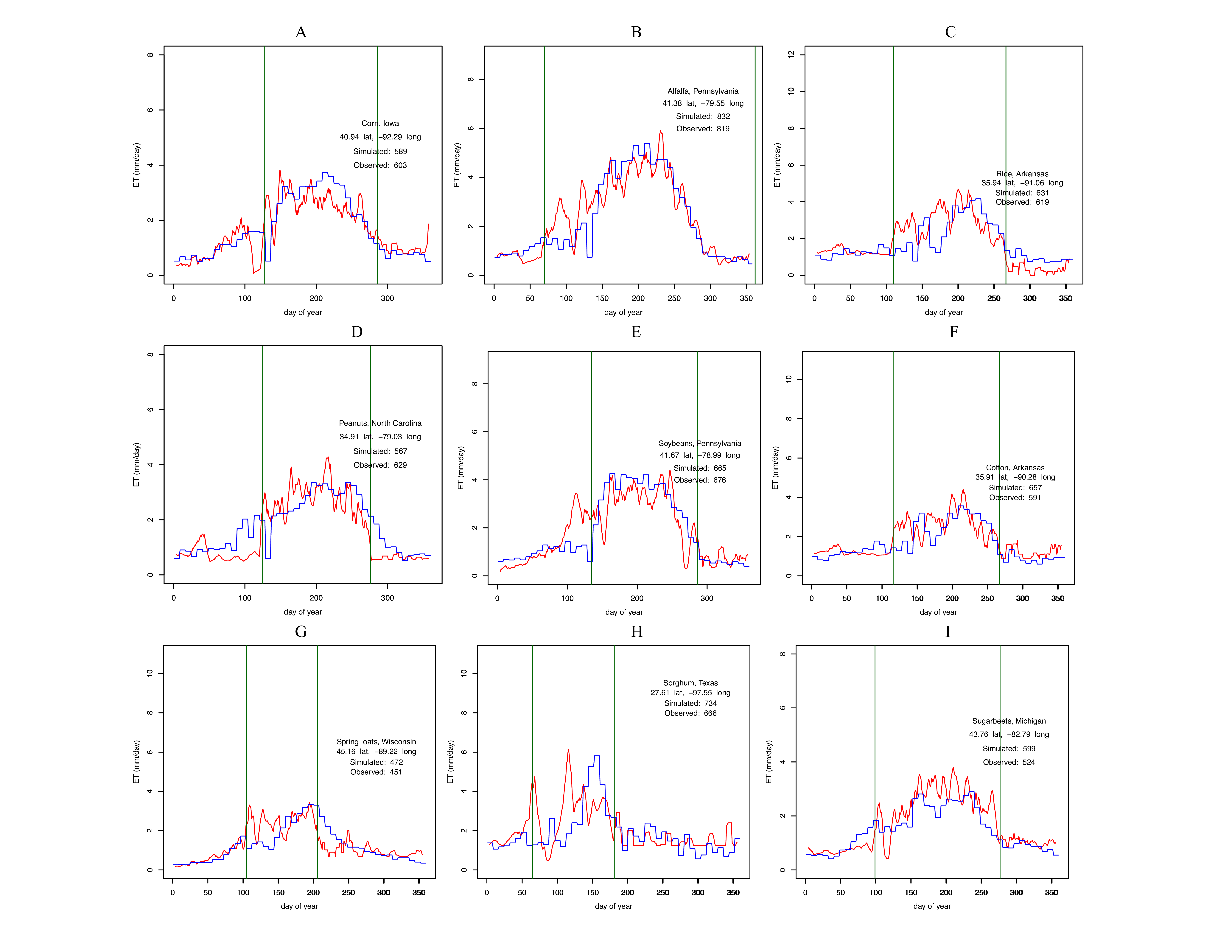

Supplement: S2 File — Table A. Kcb parameter values for perennial grassland and non-crop agricultural land cover types. Table B. Crop water balances that can be estimated for daily, seasonal, or annual time steps. Table C. Literature estimates of evapotranspiration versus modeled evapotranspiration rates. Seasonal and annual evapotranspiration ranges reported in literature sources and model by CropWatR. A range of methods are available for determining evapotranspiration at the field, landscape, and watershed scales, either via direct measurement (e.g. soil moisture measurement via lysimeters), modeling (e.g. via process-based s imulation models, or energy balances using weather data collected via satellite, local instruments, and/or remote sensing). For a summary of common methods, see Connor et al.,2011. Table C. Model performance metrics comparing the results with MODIS 16 estimates. Table E. NASS Classification categories considered in this analysis. Note that double-cropped classifications where both crops were not included among the 14 parameterized crops (e.g. lettuce / upland cotton, lettuce / barley, etc.) were excluded from the analysis. The total acreage of these classifications on the national scale was in all cases much less than 1% of the acreage of the modeled crop. Table F. NASS Accuracy assessments for crops and land types considered in this analysis. Accuracy statistics for crop and land use categories not reported in the above table are not available for 2008. These categories are: alfalfa, other hay, sugarcane, fallow/idle cropland, grassland herbaceous, and pasture/hay. Fig A. Relation between time (day since emergence), crop coefficient (Kcb) and plant height for maize. Crop coefficients are specified for three moments in time. According to the FAO 56 methods, coefficients are estimated by stepwise and linear interpolation (black line). In CropWatR, a Bezier curve (red curve) is used to interpolate daily Kcb values. Fig B. Irrigation calibration results for corn. The [file pone.0204298.s002.zip › S2 File Figures Tables/S2 Fig G.tiff]
