# Supplementary material for: Water impacts of U.S. biofuels: Insights from an assessment combining economic and biophysical models
Source: PLoS One. 2018 Sep 28;13(9):e0204298. doi: 10.1371/journal.pone.0204298 (PMC6161887; doi:10.1371/journal.pone.0204298)
Supplement: S2 File — Table A. Kcb parameter values for perennial grassland and non-crop agricultural land cover types. Table B. Crop water balances that can be estimated for daily, seasonal, or annual time steps. Table C. Literature estimates of evapotranspiration versus modeled evapotranspiration rates. Seasonal and annual evapotranspiration ranges reported in literature sources and model by CropWatR. A range of methods are available for determining evapotranspiration at the field, landscape, and watershed scales, either via direct measurement (e.g. soil moisture measurement via lysimeters), modeling (e.g. via process-based s imulation models, or energy balances using weather data collected via satellite, local instruments, and/or remote sensing). For a summary of common methods, see Connor et al.,2011. Table C. Model performance metrics comparing the results with MODIS 16 estimates. Table E. NASS Classification categories considered in this analysis. Note that double-cropped classifications where both crops were not included among the 14 parameterized crops (e.g. lettuce / upland cotton, lettuce / barley, etc.) were excluded from the analysis. The total acreage of these classifications on the national scale was in all cases much less than 1% of the acreage of the modeled crop. Table F. NASS Accuracy assessments for crops and land types considered in this analysis. Accuracy statistics for crop and land use categories not reported in the above table are not available for 2008. These categories are: alfalfa, other hay, sugarcane, fallow/idle cropland, grassland herbaceous, and pasture/hay. Fig A. Relation between time (day since emergence), crop coefficient (Kcb) and plant height for maize. Crop coefficients are specified for three moments in time. According to the FAO 56 methods, coefficients are estimated by stepwise and linear interpolation (black line). In CropWatR, a Bezier curve (red curve) is used to interpolate daily Kcb values. Fig B. Irrigation calibration results for corn. The [file pone.0204298.s002.zip › S2 File Figures Tables/Supporting Information 2 - CropWatR.docx]

Supporting Information 2: A description of CropWatR

Jacob Teter^1*^

^1^ International Energy Agency, Sustainable Technology Outlooks, Paris, France

* Corresponding author

E-mail: [Jacob.TETER@iea.org](mailto:Jacob.TETER@iea.org) (JT)

# Summary

Estimating crop water use is important to understand the effects of changing agricultural and land use patterns. This paper describes an improved process-based crop-water model, CropWatR, that can be used for high spatial and temporal resolution estimation of crop-water balances in large regions. The computations, components, and capability of the model are described and its use is illustrated by applying it to model crop-water balances for a set of crops grown in the U.S. in 2008. Validation with survey and satellite data show that the model preforms well. It can be used to designate simple irrigation scheduling rules, separately model evaporation and transpiration, and estimate volumes of seasonal and annual runoff and groundwater infiltration.

#

# 1. Introduction

Agricultural crops require a considerable amount of soil water, provided by irrigation or rainfall. Water that is transpired or evaporated from plants is not immediately available to a local catchment, and so land use decisions, such as whether to convert non-cropped land to agricultural use, or to allow primary forest growth on grassland, affect the volumes of water that return to groundwater through infiltration or run off to local ground- and surface water reservoirs.

Much research on crop-water use relies upon process-based models that trace the flows of water between the soil, a crop, and the atmosphere. Available models are often somewhat simplistic and work on a monthly time step, such as CropWat [1-3], or considerably more complicated and require parameterization to local hydrologic processes for which data covering broad geographic areas are often not available [4] , such as EPIC [5], SWAT [6, 7], and AquaCrop [8]. Moreover, even publically available models are typically implemented in non-open source software packages. Regional to national level studies of crop-water requirements, such as those conducted in water footprinting studies or water use lifecycle assessments, require a robust but adaptable, readily implementable, and above all, transparent and parsimonious process-based crop water model.

This paper describes the effort to create a simple and transparent implementation of the computations detailed in the FAO 56 Manual [9] using the *R* environment for statistical computing (R Core Team, 2015). The implementation of this program in *R* is quite similar to the *CropWat* model, with a few key differences. Key differences are that the computations in the model described here include: (1) the parameters can easily be calibrated by the user to match local conditions, (2) input parameters can be specified to match survey or GIS data, and (3) the user can easily incorporate geographic data to batch process calculations across varying locations with different weather, soil, and management parameters.

The model runs on a daily time step and estimates balances of transpired and evaporated water, groundwater infiltration and runoff, and volumes of irrigation water applied. To model each of these key water flows, it uses as its basis the algorithms for computation of the dual crop coefficient (K_cb_) as described in the report by the Food and Agriculture Organization of the United Nations, “*Crop evapotranspiration - Guidelines for computing crop water requirements - FAO Irrigation and drainage paper 56.*” [9] – henceforth referred to as FAO 56. The model developed here is flexible and can be easily specified to match planting and harvesting dates, total annual irrigation application volumes, and other parameters, based on field or survey data. Crop coefficients and other parameters can be calibrated to match and validated against field measurements and/or other estimates of evapotranspiration, such as the MODIS 16 data product [11, 12]. The model can further be used to estimate water balances in the off (or fallow) season, as well as for perennial or non-crop land uses (such as idle crop land or pasture land).

To demonstrate the calibration procedures and the validation of the model, this analysis integrates geographic and survey data from various sources, including satellite derived and interpolated weather data, satellite derived cropping patterns from the Cropland Data Layer [13, 14], and survey data on soil and cropping practices (including cropping practices and planting and harvesting dates), for the contiguous U.S. The model is then run for 18 row crops, as well as other grasslands, idle cropland, and cropland pasture, using 2008 as the validation year. All model runs were for 100 km^2^ spatial resolution grid cells.

First, daily reference evapotranspiration (ET_o_) is derived on the basis of daily weather data using the full Penman-Monteith algorithm. Next, survey and satellite data is used to specify key parameters (e.g. planting and harvesting dates, irrigation intensity, and land area irrigated). Area cropped per 10 km grid cell serves as a mask to determine the grid cells for which the daily computation of crop-water balances is run, for each crop. Finally, daily estimates of evapotranspired water are compared and, to the extent possible, validated against another estimation algorithm – the MODIS 16 8-day actual evapotranspiration (ET) product [12], and against literature values for daily evapotranspiration as measured in field trials for certain crops grown in California (in mm/day). Statistical comparisons indicate a robust fit between annual estimates of evapotranspiration as modeled by *CropWatR* and those derived by MODIS 16. The source code for implementing the algorithms using the dual crop coefficient described in FAO 56 and integrating these with geographic and survey data is available on github (at github.com/jacobteter/CropWatR [15]).

# 2. Methods

#### Model description

Our model follows closely the method documented in “*Crop evapotranspiration - Guidelines for computing crop water requirements - FAO Irrigation and drainage paper 56*” [9], or FAO 56. The documentation describes in detail the key physical processes governing the flow of water between the atmosphere, soils, and plants, and the parameters and algorithms that can be used to estimate these crop-water balances on a daily time step.

The first step in using the model is to derive reference evapotranspiration (ET_o_) using the Penman-Monteith equation. Reference evapotranspiration measures the rate of evapotranspiration (ET) from a hypothetical grass reference crop with specific characteristics under varying weather conditions but with sufficient soil water, and was developed as an empirically measurable construct to isolate soil and leaf surface evaporation and crop transpiration processes from complexities of crop type, development, and management. Together with development stage-specific crop coefficients (K_c_ and K_cb_), it can be used to estimate potential evapotranspiration of a function of the weather and the development stage of a crop. When combined with a soil water balance model, reference evapotranspiration and crop coefficients can be used to estimate water flows between the soil, crop, and atmosphere under various soil types, water deficits, and crop management practices.

## 2.1 Input data requirements

#### Weather data

To compute daily ET_o_ for the contiguous United States in 2008, we used the following data sources. Daily precipitation and minimum, mean, and maximum temperature at 800 meters resolution are from PRISM [16]. Water vapor pressure and incident shortwave radiation at 1 km resolution are from DAYMET [17-19]. All weather variables were resampled and projected to the same Albers Equal Area raster surface projection to a resolution of 10 by 10 kilometers.

Values for wind speed (converted from knots at 10 meters to meters per second at 2 meters height), and minimum and maximum relative humidity were taken from a network of meteorological stations in the U.S., Canada, and Mexico from the RDA dataset number 512.0 [20] and then spatially interpolated using thin-plate splines with elevation as a covariate, using the Tps() function of the R *fields* package, to derive a raster surface of estimated daily wind speed and minimum and maximum humidity.

The appendix provides the computations and maps summarizing the data and resulting ET_o_ spatio-temporal data, and the script “*ETo.calculation.R*” attached source code applies the FAO 56 Penman-Monteith equation (as per FAO 56 chapters 2-4) to derive a raster surface of daily ET_o_.

#### Soils

The percent soil, silt, and sand in the top meter of soil for soils with a depth of at least one meter and consisting of at least 75% soil were extracted from STATSGO2 [21]. Soil composition in each 10 km pixel was estimated as the area-weighted average of this subset of soil deemed to be suitable for agriculture. Top soil (i.e. the top 10 cm of soils with a depth of at least one meter) soil textures were used to derive raster surfaces of total evaporable water (TEW), and readily evaporable water (REW). Soil texture of the entire top meter was used to derive soil water content at field capacity (q_FC_) and at wilting point (q_WP_), as well as the depth of the soil surface layer that is subject to evaporative drying (Z_e_). In grid cells with missing soil TEW, REW, q_FC_, and q_WP_ values, these parameters were interpolated via inverse distance weighting (IDW) using the *gstat* package (and with the inverse distance power set to 0.5). The appendix shows the resulting classification of soil according to the USGS soil pyramid soil texture classification scheme (S2 Fig K in the Appendix).

#### Crop distribution data

Cropping patterns for fourteen crops – corn, cotton, rice, sorghum, soybeans, peanuts, barley, durum wheat, spring wheat, winter wheat, oats, alfalfa (which also includes hay and other pasture/hay products), sugarbeets, sugarcane – and three non-cropped land classifications – fallow/idle cropland, pasture grass, and grassland herbaceous, were extracted from the US Department of Agriculture’s Cropland Data Layer (CDL) for 2008 [13, 14] and the acreage of each crop in each 10 kilometer pixel was computed. Assessments of the accuracy for both crop land cover and non-agricultural land cover classifications have been undertaken both by NASS and by independent groups [14]. The CDL data and accuracy assessments for each crop and land use category used in this study are shown in S2 Table F of the appendix.

S2 Fig J in the Appendix shows the downscaled coverage of the fourteen crops and three non-cropped land classifications modeled in 2008, according to reclassification from cropped and other land covers modeled in the Cropland Data Layer [13]. Given the economic importance and widespread distribution of corn, soybeans, alfalfa, and winter wheat, and the widespread distribution of land cover classified as “grassland/herbaceous” (nearly 246 million acres were classified in 2008 in this category – more than all land cropped in corn, soybeans, spring and winter wheat, alfalfa, and cotton combined) these four crops and “grassland/herbaceous” land are used to illustrate the functionality of the model, including parameterization, calibration, and results.

#### Cropping calendar

The final necessary model input is data on cropping practices, including planting and harvesting dates and irrigation regimes. Crop management data was determined at the state level data from the National Agricultural Statistics Service (NASS) and included average state-level planting and harvesting dates, irrigated acreage and irrigation application intensity (in acre-feet) for 2008 [22]. The planting dates for alfalfa and ‘other hay’ are highly variable and depend on which specific crop is grown (‘other hay’ may include alfalfa in certain Southeastern states, or clover, Bermuda grass, or other wild grasses in other states. For alfalfa and other hay, planting dates were set for each state according to comparison of the resulting daily evapotranspiration with satellite derived 8-day ET averages.

## 2.2 The *CropWatR* model

Chapters 5-7 of the FAO 56 report describe the qualitative considerations, crop-, soil-, and management-specific parameters, and calculation algorithms to estimate crop evapotranspiration (ET_c_) using a single (K_c_) or dual (K_cb_ + K_e_) crop coefficient. The model developed uses the dual crop coefficient approach (described in chapter 6 of the report), as this allows the user to estimate the evaporation and transpiration components of ET separately, although the user should note that the transpiration component is typically overestimated and the evaporation component underestimated, as the former “include[s] a residual diffusive component supplied by soil water below the dry surface and by soil water from beneath dense vegetation” [9]. The model incorporates soil water stress and simple, crop-specific rules to model irrigation scheduling (Chapter 8 of FAO 56). It takes recommendations from FAO 56 Chapters 9 and 11 to parameterize non-crop and fallow (pre- and post-season) ET_c_ for “natural, non-typical, and non-pristine” vegetation types, and for non-growing periods such as bare soil, weed growth during the fallow season, and frozen or snow covered surfaces. All of the computation algorithms described in FAO 56 chapters 6-9 are used in the model, as are simplified versions of the calculations outlined in chapters 9 and 11. Source code, together with references to the relevant equations and discussion in FAO 56, is provided at a github repository file [15].

The following sections briefly describe the main parameters and computations implemented in the model, including growing season evaporation and crop transpiration (Section 2.1); root zone water balance, crop water stress, and irrigation computations implemented at daily time step (Section 2.2); non-cropland and fallow season evaporation and off-season (weed and volunteer) plant transpiration (Section 2.3); and calibration and validation procedures (Section 2.4). Section V of the Appendix provides a more thorough decription, and the attached source code references the sections and equations of FAO 56 applied in each of the functions of the *CropWatR* package.

#### Growing season evaporation and crop transpiration

Based on the crop planting and harvesting dates, the year is first split into a fallow season and a growing season. The initial, mid, and end-stage basal crop coefficients (K_cb_) are rescaled according to the relative length of the crop development stages to match the length of the growing season, and from this a curve is fit (as in Figure 37 of FAO 56) to interpolate daily K_cb_ values, mean rooting depth, and plant height. A Bezier curve is fit to the parameters in FAO 56, so that instead of level and linear changes in rooting depth, crop coefficients (K_cb_ values), and plant heights, as in FAO 56, a continuous growth curve is fit to each of these functions. S2 Fig A illustrates this minor difference in interpolation methods from the original linear interpolation used in FAO 56 by plotting K_cb_ values and crop height for corn (maize).

S2 Fig A.

The K_cb_ values in the mid and late stages are then adjusted in climates where the minimum relative humidity differs from 45% or where the wind speed is larger or smaller than 2 m/s. The remaining calculations are implemented in FAO 56, and are: deriving the daily upper limit evapotranspiration (K_c max_) and the minimum K_c_ for dry bare soil with no ground cover; using these and K_cb_ to estimate the daily soil fraction shaded by vegetation (f_c_) and its complement, the exposed soil fraction (1- f_c_); estimating the fraction of the soil surface that is wetted by irrigation or precipitation (f_w_) – which depends also on whether the crop has triggered irrigation rules in the previous day – and finally deriving a daily estimate of the exposed and wetted soil fraction (f_ew_) from these parameters. Next the evaporation reduction coefficient (K_r_), which is a measure of the proportion of the water depleted by evaporation from the topsoil, is calculated based on the previous day’s cumulative depth of evaporation, and total evaporable water (TEW) and readily evaporable water (REW), which themselves are derived based on three parameters: (1) soil water content at field capacity (q_FC_) and (2) at wilting point (q_WP_), and (3) the depth of the soil surface layer that is subject to evaporative drying (Z_e_). These three parameters vary depending on soil texture.

The above parameters are used to derive the daily soil evaporation coefficient (K_e_) – which is dependent upon the exposed and wetted soil fraction, the maximum value of crop-specific ET following a rain or irrigation event (K_c max_), and the evaporation reduction coefficient (K_r_). The above parameters are used to estimate the (day-end) deep percolation loss from the topsoil (DP_e_) and the cumulative depth of soil evaporation (D_e_) for calculation of the subsequent day’s soil-water balance. The cumulative depth of soil evaporation (D_e_) is bounded by zero (immediately following sufficiently heavy rainfall or irrigation) and the total evaporable water (TEW). Finally, daily evaporation is then calculated as the product of K_e_ and the reference evapotranspiration (ET_o_), and daily transpiration is estimated by the product of K_cb_ and ET_o_.

The above summary of computations illustrates that the soil-water balance calculations are dependent on precipitation, weather, the modeled plant growth, and irrigation over the previous day(s), and hence that the calculation of daily crop-water balances is necessarily iterative, proceeding not only from the first day of the growing season but, as implemented in this model, from the initial day of the preseason, that is, from immediately after the (modeled) harvest of the preceding year.

#### Growing season root zone water balance, crop water stress, and irrigation

Under irrigated conditions, irrigation is triggered when the water stress coefficient (K_s_) falls below some crop-specific threshold (this is true for all crops except rice, where irrigation is triggered when readily available water – RAW – drops below 0.56). Under soil water limited conditions, K_s_ falls below 1, and when there is no water stress, K_s_ equals 1. Water stress is used as a scalar to estimate decline in transpiration such that it results in a reduction in the daily ET_c_.

This section provides a brief overview of the computations of water stress calculation (For more details see FAO 56 report Chapter 8). As with TEW and REW, total available water (TAW) and RAW are derived from soil texture. Both are indicators of the capacity of soils to retain water that is available to plants in the root zone. RAW is derived from TAW and a crop-specific soil depletion fraction for no stress (p), which is the average fraction of TAW that can be depleted from the root zone below the plant experiences moisture stress and a resultant reduction in ET. This crop specific multiplier is modeled as a function of crop specific evapotranspiration (ET_c_) at ET_c_ values of less than 5 mm/day.

Daily root zone soil water depletion (D_r_) is determined as the remaining soil water leftover from the previous day’s depletion minus the sum of the day’s precipitation plus net irrigation that infiltrates the soil root zone, less runoff, crop evapotranspiration, and water loss due to deep percolation (groundwater infiltration). Daily root zone soil depletion (D_r_) is hence bounded between 0 and the TAW, and initial depletion is set at the beginning of the simulation (i.e. immediately following a hypothetical harvest from the previous season, or equivalently, at the beginning of the previous year’s fallow season) at 25% of the TAW. That is, at the end of the previous year’s growing season, 25% of TAW is assumed to be depleted. Results are nearly completely insensitive to initial assumptions into the level of root zone soil water depletion. When soil water content in the root zone is at field capacity, daily deep percolation (DP), or groundwater recharge, is calculated as the remainder of water not allocated in the previous calculations, i.e. as the next sum of precipitation and irrigation minus the net sum of runoff, evapotranspiration, and the previous day’s soil water depletion (equation 88 in FAO 56). Capillary rise is assumed to be zero as it is very close to zero when the water table is more than 1 meter below the root zone. Computations enabling the estimation of the effects of soil salinity on crop growth and crop-water balances, the reduction in yield due to moisture stress, and the interactive effects of salinity and moisture stress have not yet been incorporated into the model.

#### Non-cropland and fallow season evaporation and plant (weed and volunteer) transpiration

We follow the FAO 56 methods for estimating the dual (K_cb_) crop coefficients for natural, non-typical, and non-pristine vegetation. We adjust K_cb_ values in the initial, mid, and late seasons under conditions of sparse vegetation for deriving and calibrating K_cb_ based on leaf area index (LAI) measurements, effective ground cover, and the ratio between actual and potential crop yields. Recommendations in FAO 56 (chapter 9) were used to parameterize the fallow season (weed and volunteer plant growth) – values of K_cb_, plant heights, and rooting depths for perennial vegetation. Specifically, given the low accuracy in distinguishing among fallow/idle cropland, pasture grass, and grassland herbaceous land covers in the CDL, similar K_cb_ values in the initial, mid, and late season; crop heights; and rooting depths have been assumed for all of these land covers nationwide. Note that this does not imply that nationwide heights, rooting depths, and growing seasons were uniform for these land cover classifications; rather, as with all other crops, the growing season and potential transpiration and growth of perennials is sensitive to weather and soil parameters that vary in space and time. S2 Table A shows the parameter values adopted for these land types.

When temperatures fall below zero for sustained periods of time, the daily water balance calculations are implemented assuming that K_cb_ equals 0, otherwise low K_cb_ values have been selected to represent weed- or volunteer plant growth based on the recommendations in chapter 9. Specifically, an initial K_cb_ value of 0.25, root depth of 0.1 meters, and p value of 0.1 (appropriate for shallow-rooted weeds) is adopted and these values increase daily – the K_cb_ value increases by 0.005 and the root depth by 0.002 per day. Since the soil water balances automatically adhere to the conservation of mass in the K_cb_ calculations, there is no risk in overestimating actual weed/volunteer K_cb_. At worst, the fraction of evapotranspiration represented by weeds (and not by evaporation) will be over- or underestimated.

S2 Table A. K_cb_ parameter values for perennial grassland and non-crop agricultural land cover types.

| Land type | K_cb_ and plant height | | | |
| --- | --- | --- | --- | --- |
|  | K_cb_initial_ | K_cb_mid_ | K_cb_end_ | Max height (m) |
| Idle cropland | 0.2 | 1.15 | 0.6 | 1 |
| Pasture grass | 0.2 | 1.15 | 0.4 | 0.7 |
| Grassland herbaceous | 0.2 | 1.15 | 0.6 | 1.25 |
|  | Rooting depths (m) and p value | | | |
|  | Base rooting depth | Max rooting depth | Mean rooting depth | p value |
| Idle cropland | 1 | 1.5 | 1.3 | 0.55 |
| Pasture grass | 1 | 1.5 | 1.3 | 0.55 |
| Grassland herbaceous | 1 | 1.5 | 1.3 | 0.55 |
|  | Crop stages (days or proportions in each stage) | | | |
|  | Initial | Developing | Mid | Late |
| Idle cropland | 20 | 80 | 210 | 30 |
| Pasture grass | 20 | 100 | 150 | 50 |
| Grassland herbaceous | 20 | 50 | 240 | 30 |

No further adjustments have been made to calculate water-balances for frozen or snow covered surfaces, as the reference evapotranspiration (ET_o_) approach has been shown to perform inadequately under such conditions. The fraction of off-season water balances that will be inaccurately estimated as a result of failure to incorporate frozen or snow covered soils will clearly increase at higher latitudes and regions with colder winters, but given the fact that most changes of interest to researchers modeling crop-water balances, such as changing cropping and land use patterns, have little to no direct impact on soil conditions under sustained sub-zero temperatures, correctly modeling such conditions is not a priority and does not significantly effect the fidelity of the overall model given the current advised applications.

#### Irrigation calibration

The area of irrigated land was set to match state level survey data [22]. Irrigation intensity was then calibrated to match state-level survey reported averages by first adopting crop-specific irrigation rules that match mean nationwide irrigation intensity, and then multiplying these by state-level scalar adjustment factors. Remaining deviations from state-level surveyed irrigation intensities were for the most part small, and no attempt was made to calibrate the irrigation intensity further via manual state-by-state adjustments. The resulting calibration for irrigated corn grown in 2008 is shown in S2 Fig B. Density plots and maps showing the calibration fits for all other irrigated crops are given in S2 Fig L in section IV of the appendix.

S2 Fig B.

#### Calibrating and validating crop water balances

The above procedure yields estimates of the six daily, seasonal, and annual (i.e. growing and fallow season) water balances shown in S2 Table B. Water balances can be expressed in mm per relevant time period (day, season, or year), or converted to inches or acre-feet for convenient comparison with literature values (as evapotranspiration is often expressed in inches and irrigation withdrawals in acre-feet in the U.S.). Currently, irrigation volumes are modeled at the point of release to the soil root zone, hence users are advised to apply relevant upstream multipliers as necessary to back-calculate irrigation water withdrawal estimates. Transpiration can be disaggregated between crop transpiration (in the growing season) and weed/volunteer transpiration (in the fallow season), as can all the other water balances.

S2 Table B. Crop water balances that can be estimated for daily, seasonal, or annual time steps.

| Daily water balances |
| --- |
| Transpiration |
| Evaporation |
| Irrigation |
| Runoff |
| Root zone soil water depletion |
| Deep percolation (groundwater infiltration) |

Two approaches were used to validate and calibrate model outputs. The initial step was to identify the locations with the greatest cropped density in each of the HUC 2 regions for each of the crops modeled. At each of these locations, daily evaporation and transpiration volumes as well as input weather, soil, and crop characteristics (e.g. rooting depth, soil coverage fraction, etc.) and input model parameters (e.g. derived reference evapotranspiration or ET_o_, the dual crop coefficient, K_cb_, irrigation volumes, soil water infiltration, etc.) were extracted for the fallow preseason, growing season, and fallow postseason. These parameters were inspected on a case-by-case basis first to verify that the relationships matched the daily algorithms prescribed in the FAO 56 manual [23], to identify and eliminate any erratic or unreasonable spikes (i.e. daily changes greater than ± 200%) in daily water balances, and to verify that the results matched (daily and seasonal) volumes of transpiration, evaporation, runoff, and groundwater infiltration cited in a crop ecology textbook [24] and online sources (e.g. state USDA sources, university agronomy presentations, etc.).

In addition, detailed comparisons of modeled growing season water balances for corn and rice planted in California’s Central Valley were performed and a broader literature review of daily and seasonal evapotranspiration profiles for these and other crops in states west of the Mississippi was performed. It was discovered that the daily and seasonal ET values estimated by the model were far closer to literature-cited values than the MODIS 16 satellite derived estimates for cropping locations in these regions. This leads to the hypothesis that the parameters used in the MODIS 16 estimation are insufficiently sensitive to detect field and landscape scale alterations in crop-water balances resulting from irrigated agriculture in the American West.

Upon obtaining model results, two checks of the models and methods were performed. First, resulting estimates of daily evaporation and transpiration are compared with 8-day average evapotranspiration (ET) as estimated to derive the MODIS 16 data product [12]. Model performance metrics for accuracy (absolute relative error), bias (mean relative error), reliability (absolute error below a threshold of 30%), and efficiency (the Nash-Sutcliffe model efficiency coefficient) were calculated by comparing the resulting 8-day average ET values and ET summed over the growing season and the entire year to MODIS 16 estimates. Second, modeled ET estimates are compared with literature derived ET estimates at field sites and at the state level across the contiguous US. These final validation checks are reported in the Result section.

# 3. Results

## 3.1 Spatial and Graphical Representations

Model results can be presented in a number of basic formats, including maps, bar charts, and violin charts or box plots. This is illustrated below using the example of corn. The right- and left-hand columns of S2 Fig C map two kinds of basic model results. Here the annual aggregated water flows are classified into the following five categories (1) *crop (i.e. corn) transpiration*, (2) *evaporation plus weed / volunteer (i.e. off-season) transpiration*, (3) *irrigation* (applied at the roots), (4) *runoff,* and (5) *groundwater infiltration* (note that this a reclassification of the basic water flows shown in S2 Table B). The maps in the right-hand column show of the annual (off-season plus growing season) total volumes of water flows used to grow corn in 2008 in total annual millimeters. The maps in the left-hand column show the water budget for same categories, but here the volumes are multiplied by the number of acres grown in corn in each grid cell. Hence, the right-hand maps show the total (aggregated annual) water requirements for corn grown across the contiguous U.S. in 2008. The model allows to easily create maps like those shown in S2 Fig C for all the water balances shown in S2 Table B, or for a combination or subset of those balances, at any desired temporal resolution (e.g. daily, monthly, or for the growing season, fallow season, or, as shown here, for the entire year).

The model enables the user to present results easily in various other non-spatial formats. One useful way to present results is to aggregate or summarize (e.g. showing means, medians, or entire distributions of) water use across spatial regions. S2 Fig D is a bar chart of the total annual water requirements for each of the same five categories for each of twelve Water Resource Regions (equivalent to Hydrologic Unit Code 2 or HUC 2). The results are superimposed on a map of corn cropped in each of these (labeled) HUC 2 regions. That is, S2 Fig D aggregates the water volumes shown in the left-hand column of S2 Fig C in each HUC 2 region – units are adjusted to *million* annual acre-feet of water going to corn in each region. The average water use in the right-hand column of S2 Fig B can also be shown as a bar chart, and similar bar charts can be made for any other relevant regional disaggregation (e.g. counties, HUC 4, HUC 6, or HUC 8 regions, states, USDA crop growing regions, etc.).

S2 Fig E shows a violin plot giving the *distribution* of water balances across all pixels in each of the zones. A violin plot provides similar information to a standard box plot but shows the entire distribution (across all pixels) rather than giving discrete values. The standard box plot is another option for visualizing distributions. Within each violin plot is the box plot showing the median (white dot), 50% range (black box), and 25/75% range (lines). The map overlays the boundary of the HUC 2 regions a map of corn growth (concentrated in the Midwest and Corn Belt), given in acres grown per 10 by 10 kilometer pixel. The bar chart estimates total water use in million acre-feet in each HUC 2 in 2008. The figure illustrates the wide distribution in transpiration rates achievable between primarily regions that cultivate corn primarily under rainfed or dryland conditions (e.g. in hydrologic regions spanning the Midwest and Corn Belt: Missouri, Upper Mississippi, Great Lakes, and Ohio), versus the much higher transpiration rates and water requirements of irrigated corn cultivation achievable in Frontier and Western States (e.g. Missouri, Upper and Lower Colorado, California). It also shows the wide distribution in runoff, particularly in the South Atlantic-Gulf, Lower Mississippi, Arkansas Red-White, and Texas-Gulf water resource regions. This runoff is a function high precipitation events at daily resolution, high annual precipitation rates, and variations in soil types.

S2 Fig C.

S2 Fig D.

S2 Fig E.

## 3.2 Validation of Evapotranspiration

Modeled daily evapotranspiration (ET) was compared against the MODIS 16 ET data product, an established algorithm that uses MODIS satellite data to model average eight-day actual ET at ~1 km resolution. Locations were chosen for each crop and land use classification in each of the 18 water resource regions (or Hydrologic Unit Code 2 [HUC2] classifications) whenever possible. Coordinates were chosen where the density of cropping (or uncultivated land) was greatest in each HUC2 region. S2 Fig M in Appendix shows daily modeled ET plotted together with MODIS 16 derivations of 8-day average ET (as shown below in S2 Fig F), and S2 Fig N in Appendix maps the locations chose for validation against MODIS. In most instances, the models matched with fairly high fidelity. S2 Fig G shows a few representative comparisons between the daily ET modeled by the process-based model described here, versus the 8-day average ET estimated by MODIS 16.

Although MODIS 16 resolution (~1 km by 1 km) is substantially higher than the resolution at which the crop water balance estimates are run (~10 km by 10 km), due to the fact that evapotranspiration is extremely heterogeneous on a plot scale (i.e. at resolutions of less than 1 square km), the MODIS 16 data product relies on inputs with insufficient resolution to estimate evapotranspiration of cropped land in all cases except where large areas of land are cropped in a monoculture. In contrast, by using crop-specific inputs and representative soil and daily weather parameters, the model developed here provides estimates of ET that fall within ranges estimated by plot-scale experiments conducted at agricultural research stations across the country. S2 Table C lists selected validation sources from online and literature sources, together with ET as estimated by pan methods, and compared with the outputs of the model developed here.

Locations were identified for each crop and land use pattern where contiguous cropped area was sufficiently large to use MODIS as a validation tool. S2 Fig F shows the validation locations for corn in black, most of which are in the Corn Belt. Validation locations of all crops and land types are shown in S2 Fig N of the Appendix.

S2 Fig F.

S2 Fig G.

Differences between ET as modeled by the tool developed here and as estimated by MODIS 16 can be attributed to many factors. Aside from the many uncertainties stemming from the modeling (e.g. conceptual and parameter uncertainty, measurement error), some of the mismatch between the modeled and satellite-derived ET can be attributed to differences in planting and harvesting dates and in actual irrigation scheduling. Specifically, since the planting and harvesting dates were taken as the mean value of state-level survey reported dates, and since the irrigation rules were triggered by crop-specific water soil moisture thresholds and then calibrated by a simple scalar to match state average irrigation intensity levels, some mismatch with actual planting and harvesting dates and irrigation regimes is to be expected.

Differences in planting and harvesting dates are likely the cause of some of the mismatches shown above – for instance, in the case of alfalfa grown in Vermont shown in S2 Fig G(B), the growing season seems to be earlier at the location shown, in the case of peanuts grown in North Caroline (S2 Fig G(D)) it seems to be longer, and for spring oats in Wisconsin (S2 Fig G(E)) it is perhaps later. Similarly, irrigation scheduling as chosen by the model seems to be more aggressive in the early season than the actual irrigation regime, and this is the likely cause of early peaking of modeled EV in the cases of corn in Iowa (S2 Fig G(A)), spring wheat in North Dakota (S2 Fig G(J)), and sorghum in Texas (S2 Fig G(H)). These discrepancies can be corrected in practice by model users via site-by-site input modifications, however as the model is designed for application over wide geographic ranges and the results are acceptable to a first order of approximation, such calibration is only advised for plot level studies or when a gross mismatch has been identified.

S2 Table C. Literature estimates of evapotranspiration versus modeled Evapotranspiration rates.

| Source | Location | Crop | Method(s) | Period | Cited ET  (mm) (mean) | Modeled ET (mm) (mean) | Irrigated | Link |
| --- | --- | --- | --- | --- | --- | --- | --- | --- |
| Kanemasu, 1977a | Kansas | Corn | Measurements; simulation model | Growing season (May-August) | 500 | 410 - 590 (445) | Unspecified | [Kansas State](http://www.ksre.ksu.edu/historicpublications/Pubs/SRL32.pdf) |
| Hatfield & Prueger, 2003 | Central Iowa | Corn | Measurements | Annual | 290 - 580 (410) | 485 - 625 (550) | Both | [Link](http://cdn.intechopen.com/pdfs-wm/22687.pdf) |
| Hatfield & Prueger, 2003 | Central Iowa | Corn | Measurements | Growing season (April - Oct) | 275 - 500 | 400 - 450 (430) | Both | [Link](http://cdn.intechopen.com/pdfs-wm/22687.pdf) |
| Hatfield & Prueger, 2003 | Walnut Creek, Iowa | Corn | Measurements | Growing season (May-August) | 185 -275 | 380 - 445 (400) | Unspecified | [Link](http://cdn.intechopen.com/pdfs-wm/22687.pdf) |
| Hatfield & Prueger, 2003 | Walnut Creek, Iowa | Corn | Measurements | Growing season (May-August) | 210 - 240 | 385 - 425 (390) | Unspecified | [Link](http://cdn.intechopen.com/pdfs-wm/22687.pdf) |
| Serbina & Miller, 2014 | Eastern Colorado | Corn | Measurements; METRIC model | Growing season (May-Sept) | 515 - 570 | 325 - 675 (450) | Yes | [Link](http://pubs.usgs.gov/of/2014/1108/pdf/ofr2014-1108.pdf) |
| Medellín-Azuara & Howitt, 2013 | Sac.-San Joaquin, CA | Corn | SEBAL; simulation models | Growing season (March-Sept) | 660 - 830 | 600 - 860 (730) | Yes | [Link](http://www.delta.ca.gov/%5C/res/docs/landscapes/UCD_ET_Report_9-2013.pdf) |
| Masoner et al., 2003 | Lake Altus, OK / TX | Corn | Radation method | Growing season (Feb - July) | 730 - 820 | 390 - 565 (440) | Yes | [Link](http://pubs.usgs.gov/wri/wri034155/pdf/wri034155.pdf) |
| Howell et al., 1997 | Bushland, Texas | Corn | Measured; multiple models | Growing season (Sept - July) | 700 - 830 | 320 - 565 (400) | Yes |  |
| Kanemasu, 1977a | Kansas | Soybeans | Measurements; simulation model | Growing season (May-August) | 520 | 390 - 500 (410) | Unspecified | [Kansas State](http://www.ksre.ksu.edu/historicpublications/Pubs/SRL32.pdf) |
| Masoner et al., 2003 | Lake Altus, OK / TX | Soybeans | Radation method | Growing season (April - Sept) | 730 - 820 | 450 - 520 (465) | Yes | [Link](http://pubs.usgs.gov/wri/wri034155/pdf/wri034155.pdf) |
| Masoner et al., 2003 | Lake Altus, OK / TX | Alfalfa | Radation method | Growing season (Jan - Dec) | 990 - 1115 | 780 - 1050 (840) | Yes | [Link](http://pubs.usgs.gov/wri/wri034155/pdf/wri034155.pdf) |
| Medellín-Azuara & Howitt, 2013 | Sac.-San Joaquin, CA | Alfalfa | SEBAL; simulation models | Growing season (March-Sept) | 905 - 980 | 400 - 1200 (680) | Yes | [Link](http://pubs.usgs.gov/wri/wri034155/pdf/wri034155.pdf) |
| WPAWS, 2000 | Mount Vernon, WA | Alfalfa | Kimberly-Penman | Growing season (January-Dec) | 770 | 710 - 900 (775) | Unspecified | [USGS](http://wa.water.usgs.gov/projects/wria01/data/refet.htm) |
| Shewmaker et al., 2013 | Kimberly, Idaho | Alfalfa | Kimberly-Penman | Growing season (April - Sept) | 900 | 330 - 865 (460) | Yes | [U. of Idaho](http://www.extension.uidaho.edu/forage/Fact%20Sheets/Alfalfa%20Irrigation%20Facts%202013%20Final%5B1%5D.pdf) |
| Serbina & Miller, 2014 | Eastern Colorado | Alfalfa | Measurements; METRIC model | Growing season (April - Oct) | 660 - 680 | 530 - 840 (560) | Yes | [Link](http://pubs.usgs.gov/of/2014/1108/pdf/ofr2014-1108.pdf) |
| Samis & Smeal, 1983 | Clovis, New Mexico | Winter wheat | Measured; multiple models | Growing season (October - June) | 285 - 615 (445) | 300 - 420 (325) | Unspecified |  |
| Jensen & Sletten, 1965 | Bushland, Texas | Winter wheat | Measurements | Growing season (October - July) | 450 - 710 | 300 - 865 (435) | Yes |  |
| Howell et al., 1997 | Bushland, Texas | Winter wheat | Measured; multiple models | Growing season (Sep - July) | 790 - 930 | 300 - 865 (435) | Yes |  |
| Masoner et al., 2003 | Lake Altus, OK / TX | Winter wheat | Radation method | Growing season (August - Dec) | 660 - 1070 | 380 - 900 (440) | Yes | [Link](http://pubs.usgs.gov/wri/wri034155/pdf/wri034155.pdf) |
| Kanemasu, 1977a | Kansas | Winter wheat | Measurements; simulation model | Growing season (August - Dec) | 380 - 480 | 325 - 450 (350) | Unspecified | [Kansas State](http://www.ksre.ksu.edu/historicpublications/Pubs/SRL32.pdf) |
| Kanemasu, 1977b | Manhattan, Kansas | Winter wheat | Measurements; Landsat model | Growing season (August - Dec) | 410 - 490 (430) | 460 - 500 (470) | Unspecified |  |
| McAuley et al., 1978 | Goodwell, Oklahoma | Sorghum | Measurements | Growing Season (June - Nov) | 330 - 760 | 550 - 630 (570) | Yes |  |
| Masoner et al., 2003 | Lake Altus, OK / TX | Sorghum | Radation method | Growing season (April - Sept) | 650 - 730 | 415 - 500 (430) | Yes | [Link](http://pubs.usgs.gov/wri/wri034155/pdf/wri034155.pdf) |
| Kanemasu, 1997a | Kansas | Sorghum | Measurements; PEM | Growing season (June-Sept) | 500 | 330 - 610 (385) | Unspecified | [Link](http://www.ksre.ksu.edu/historicpublications/Pubs/SRL32.pdf) |
| Zeng & Heilman, 1997 | West Texas (Lubbock) | Sorghum | ENWATBAL simulation model | Growing season (March-August) | 565 | 460 - 550 (475) | Unspecified | [Article](http://link.springer.com/article/10.1007%2FBF00863616) |
| Howell et al., 1997 | Bushland, Texas | Sorghum | Measured; multiple models | Growing season (Sept - July) | 535 - 640 | 460 - 550 (475) | Yes |  |
| Masoner et al., 2003 | Lake Altus, OK / TX | Cotton | Radation method | Growing season (Feb - July) | 850 - 960 | 660 - 770 (680) | Yes | [Link](http://pubs.usgs.gov/wri/wri034155/pdf/wri034155.pdf) |
| Zeng & Heilman, 1997 | West Texas (Lubbock) | Cotton | ENWATBAL simulation model | Growing season (Feb - July) | 360 | 130 - 500 (400) | No | [Link](http://link.springer.com/article/10.1007%2FBF00863616) |
| Zeng & Heilman, 1997 | West Texas (Lubbock) | Cotton | ENWATBAL simulation model | Growing season (Feb - July) | 415 | 360 - 800 (530) | Yes | [Link](http://link.springer.com/article/10.1007%2FBF00863616) |
| Fisher, 2012 | Stoneville, Mississippi | Cotton | Lysimeter; Penman | Growing season (April-Sept) | 500 - 570 | 415 - 500 (430) | Yes |  |
| Fisher, 2012 | Stoneville, Mississippi | Cotton | Penman-Monteith | Growing season (April-Sept) | 615 - 740 | 415 - 500 (430) | Yes |  |
| Serbina & Miller, 2014 | Eastern Colorado | Sugar beets | Measurements; METRIC model | Growing season (April - October) | 530 - 575 | 380 - 650 (445) | Yes |  |
| Samis & Smeal, 1983 | Clovis, New Mexico | Spring barley | Measured; multiple models | Growing season (May - Sept) | 335 - 525 (400) | 300 - 800 (500) | Yes | [Link](http://www.wrri.nmsu.edu/publish/techrpt/tr179/tr179.pdf) |
| Robertson & Stark, 2003 | Southern Idaho | Spring barley | Measured (estimated mean) | Growing season (April - Sept) | 650 | 700 - 900 (755) | Yes | [U. of Idaho](http://www.extension.uidaho.edu/forage/Fact%20Sheets/Alfalfa%20Irrigation%20Facts%202013%20Final%5B1%5D.pdf) |
| Serbina & Miller, 2014 | Eastern Colorado | "small grains" | Measurements; METRIC model | Growing season (April - August) | 435 - 445 | 300 - 800 (500) | Unspecified | [Link](http://pubs.usgs.gov/of/2014/1108/pdf/ofr2014-1108.pdf) |
| Masoner et al., 2003 | Lake Altus, OK / TX | Peanuts | Radation method | Growing season | 530 - 690 | 450 - 640 (540) | Yes | [Link](http://pubs.usgs.gov/wri/wri034155/pdf/wri034155.pdf) |
| McAuley et al., 1978 | Fort Cobb, Oklahoma | Peanuts | Measurements | Growing Season (May - October) | 435 - 615 | 450 - 610 (520) | Yes |  |
| Sumner & Jacobs, 2005 | Ferris farms, Florida | Grassland pasture | Measured; multiple models | Annual | 690 - 900 | 640 - 910 (740) | No |  |
| Douglas et al., 2009 | Florida | Grassland pasture | Measured; multiple models | Annual | 675 - 1090 | 660 - 1000 (735) | No |  |
| Serbina & Miller, 2014 | Eastern Colorado | Grassland pasture | Measurements; METRIC model | Annual | 565 - 670 | 215 - 580 (250) | No | [Link](http://pubs.usgs.gov/of/2014/1108/pdf/ofr2014-1108.pdf) |

Seasonal and annual evapotranspiration ranges reported in literature sources and model by CropWatR. A range of methods are available for determining evapotranspiration at the field, landscape, and watershed scales, either via direct measurement (e.g. soil moisture measurement via lysimeters), modeling (e.g. via process-based s imulation models, or energy balances using weather data collected via satellite, local instruments, and/or remote sensing). For a summary of common methods, see Connor et al.,2011 [24].

#### Model performance

S2 Table D reports model performance metrics for *accuracy* (absolute relative error – equation 1 below), *bias* or systematic under- or overprediction (mean relative error – equation 2), *reliability* (absolute error below a threshold of 20% - equation 3), and *efficiency* (the Nash-Sutcliffe model efficiency coefficient – equation 4) for all crop and land cover classifications except the cold season small grain varieties (fall and winter wheat, fall barley, and fall oats). The Nash-Sutcliffe efficiency is a commonly used measure of predictive accuracy of a model, and is most widely used in hydrologic models to measure accuracy against measured discharge. NSE values can range from ∞ to 1, and values ranging from 0 to 1 perform better than the mean of the observed data.

Equations for the performance metrics are identified below. In each instance, *Obs* stands for observed (MODIS 16) values, and *Sim* for modeled results from the *CropWatR* model. *Correlations* are calculated using the Pierson’s correlation coefficient. Correlations and reliability metrics were calculated using two methods: first by comparing 8-day average ET values from the model with the direct MODIS 16 data product (8-day average) and second by comparing the ET summed over the entire year to annual summed MODIS 16 estimates across all validation locations. Model efficiency was compared at the 8-day level. Scatterplots showing daily and annual correlations, on the left and right hand side, respectively, for corn, soybeans, alfalfa, and sorghum are shown in S2 Fig H below.

*Model accuracy* – ***a***bsolute ***p***ercentage ***e***rror: $APE=\left| \frac{O_{i}-M_{i}}{M_{i}} \right|\times100$ (Equation 1)

*Model bias* – ***m***ean ***r***elative ***e***rror: $MRE= \frac{1}{n}\sum_{i=1}^{n} |\frac{O_{i}-M_{i}}{M_{i}}| \times100$ (Equation 2)

*Model* *reliability* – absolute error below 20%: $Reliability= \frac{\left| MRE \right|[|MRE| > 0.2]}{n}$ (Equation 3)

*Model efficiency* – ***N***ash-***S***utcliffe model ***e***fficiency: $NSE= 1-\frac{\sum_{i=1}^{n} ({O_{i}-M_{i})}^{2}}{\sum_{i=1}^{n} ({O_{i}-\bar{O}_{i})}^{2}}$ (Equation 4)

Absolute relative error (*accuracy*) falls within the range of ±22% for all crops. Bias measures the average absolute relative error (i.e. the mean relative error); annual estimates of ET are within ±10% for all crops except spring wheat and rice, and for these crops it is within ±15% of MODIS estimates. Patterns of ET correspond fairly well both within a year, as shown by average 8-day correlations between the MODIS 16 and modeled ET ranging from 0.58 (for spring wheat) to 0.85 (corn). The range of correlations across validation sites is similar – from 0.55 (rice) to 0.96 (spring wheat). Interestingly, the model bias seems to be slightly *underestimating* annual ET relative to the MODIS 16 for most crops – this may be a consequence of the area bound by (i.e. integral of) Bezier splines being less than the area enclosed by a simple linear interpolation of crop coefficients (as shown in S2 Fig A).

Future refinements may focus on adapting crop coefficients to account for this systematic underestimation. Annual model reliability across all crops except alfalfa, rice, and durum wheat was 1, i.e. annual estimated annual evapotranspiration was within 20% of MODIS 16 actual ET for the majority of comparison sites identified. The model *reliability* performance indicates that the majority of eight-day readings are within 0.40 with a standard deviation of ± 0.078. Model efficiency is positive at all validation locations with the exception of a few pasture grass sites and averages 0.45 with a standard deviation of ± 0.20 indicating that, even at a the maximum feasible temporal resolution (i.e. eight-days), the model performs consistently better than a simple assumption of mean values.

S2 Table D Model performance metrics comparing the results with MODIS 16 estimates.

| Crop | # obs. | Accuracy | Correlations | | Bias | Reliability | | Efficiency |
| --- | --- | --- | --- | --- | --- | --- | --- | --- |
|  |  | annual | 8-day measure | annual | annual | 8-day | annual | 8-day measure |
| Corn | 327 | 0.02% - 15.5% | 0.85 (0.72 - 0.93) | 0.77 | 4.90% | 0.44 | 1 | 0.70 (0.33 - 0.86) |
| Soybeans | 122 | 0.3% - 15.3% | 0.84 (0.69 - 0.91) | 0.77 | 5.90% | 0.41 | 1 | 0.68 (0.37 - 0.83) |
| Alfalfa | 84 | 0.3% - 20.7% | 0.83 (0.61 - 0.91) | 0.78 | 7.7% | 0.51 | 0.940 | 0.63 (0.14 - 0.83) |
| Pasture grass | 38 | 0.03% - 19.0% | 0.80 (0.48 - 0.93) | 0.82 | 9.39% | 0.55 | 1 | 0.53 (-0.26 - 0.84) |
| Durum wheat | 3 | 1.5% - 21.2% | 0.75 (0.71 - 0.79) | 0.92 | 8.87% | 0.34 | 0.667 | 0.77 (0.70 - 0.84) |
| Spring wheat | 9 | 0.1% - 12.6% | 0.58 (0.49 - 0.78) | 0.96 | 12.23% | 0.30 | 1 | 0.14 (0.03 - 0.28) |
| Spring barley | 8 | 0.5% - 16.6% | 0.65 (0.52 - 0.83) | 0.91 | 8.17% | 0.39 | 1 | 0.30 (0.06 - 0.64) |
| Rice | 12 | 5.08% - 20.2% | 0.69 (0.58 - 0.76) | 0.55 | 11.54% | 0.27 | 0.917 | 0.30 (0.10 - 0.47) |
| Peanuts | 15 | 0.37% - 17.8% | 0.68 (0.57 - 0.83) | 0.69 | 8.14% | 0.37 | 1 | 0.25 (0.03 - 0.63) |
| Cotton | 35 | 0.10% - 16.0% | 0.79 (0.68 - 0.91) | 0.86 | 7.83% | 0.40 | 1 | 0.52 (0.14 - 0.82) |
| Spring oats | 8 | 1.2% - 15.3% | 0.71 (0.56 - 0.87) | 0.52 | 8.43% | 0.40 | 1 | 0.29 (0.01 - 0.64) |
| Sorghum | 39 | 0.13% - 19.1% | 0.75 (0.56 - 0.90) | 0.97 | 8.87% | 0.33 | 1 | 0.36 (0.03 - 0.68) |

S2 Fig H.

The match between the model and the MODIS 16 algorithm was found to be sufficiently robust in regions to justify considering the MODIS 16 as an acceptable, but not ideal, gauge against which to report measures of model fit. As discussed in greater detail below, MODIS 16 lacks sufficient resolution to capture the signal of field and landscape scale ET when crops are grown on fields of substantially less than 1 square kilometer. Model performance metrics for accuracy, bias, reliability, and efficiency are reported for these states, but despite acceptable performance across all metrics, given the uncertainty likelihood that MODIS 16 estimates are themselves imprecise, they are not considered to be sufficiently reliable for the purposes of either validating or discrediting the model. For this reason, literature estimates of ET were collected as a further test of model performance.

The lack of suitable validation sites in the case of wheat, oats, and barley varieties seems to be a consequence of insufficient resolution in the MODIS 16 product – the signal of the ET profile of durum and spring wheat and spring barley is likely ‘diluted’ by the wider landscape ET, making the MODIS 16 an unreliable instrument for validation. For sake of comparison, the typical soy field in Iowa is about 0.25 km^2^, and soy fields range from 0.01 to 1.3 km^2^ [25]. Indeed no locations were identified where MODIS 16 had sufficient resolution to capture ET profiles of fall and winter small grain varieties.

An independent method of gauging the accuracy and reliability of the model is to compare seasonal and annual ET estimates against literature cited values. Evapotranspiration is derived either by direct measurement of soil moisture, typically using lysimeters, or via a range of physical models that incorporate diverse parameters (e.g. readings of pan evaporation, atmometers, or remote sensing measurements, or satellite measurements of surface temperature, heat flux, albedo, climatic parameters for deriving potential evapotranspiration, etc.) to estimate energy and water exchanges across the soil, plant, and atmosphere.

S2 Table D compares literature cited ET estimates with *CropWatR* model outputs for crops modeled by across the contiguous US. Methods used in each summary are summarized as either direct measurement (e.g. via lysimeter), models, or a combination of both. The period covered (seasonal or annual) is indicated for the model estimated evapotranspiration, and the ET modeled by *CropWatR*. Some studies indicate whether the crop ET measurements are for irrigated, rainfed, a combination of both, others do not specify. Links are given to publically available data – published studies, including those that require academic affiliation to access, are cited in the references. The range of ET within and among studies is a function of the geographic extent (and hence variability in weather and soil characteristics), number of years, and variability in cropping practices. Most modeled results are within the range of cited literature. Some seemingly contradictory results were found in the literature itself, though this may also reflect differences in cultivation practices, soil, or inter-annual variability.

**4. Discussion**

The model described above serves as a simple and transparent implementation of the computations detailed in the FAO 56 Manual [9]. The implementation of this program in *R* is quite similar to the *CropWat* model, with a few key differences. First, the computations are transparent and the parameters can easily be calibrated by the user to match local conditions. Moreover, as illustrated by the above application, input parameters – including but by no means limited to irrigation intensity or planting and harvesting dates – can be specified to match survey or GIS data. The second major modification is that the model easily downloads and incorporates geographic data and enables batch processing of calculations across varying locations with different weather, soil, and management parameters. The final minor difference is the smoothed Bezier curve interpolation of dual crop coefficients from the initial to the mid and late season, and of plant height and rooting depth. It is impossible to ascertain the exact differences between this model and *CropWat* (which is not publically available). However, given the transparency of this model, users are free to download the *CropWatR* model and compare with CropWat or any other models to which they have access.

A systematic validation of the model accuracy, bias, reliability, and efficiency proved impossible due to a dearth of empirical data on national evapotranspiration balances for the crops modeled. In addition, the modeled runoff and groundwater infiltration volumes can serve at best only as useful first-approximations of plot and landscape scale processes, as the model necessarily trades off low fidelity of fine-scale hydrologic processes for allowing modeling over wide geographic areas and minimal and easily understood and calibrated parameters – in the interests of simple implementation and transparent and maximally accurate results over a wide geographic scope. In conclusion, the model does not seek to replicate the fidelity of fine-scale hydrologic models such as SWAT [6] or EPIC [5], but instead makes the simple crop-water balance equations very similar to those implemented in *CropWat* [1] available for quick, transparent, high-resolution and wide scope modeling. The model is ideally suited to studies seeking to estimate absolute levels or changes in water volumes induced by land use changes of basic crop-water budgets flows: transpiration, evaporation, irrigation, and approximations of runoff and groundwater infiltration. In particular, the soil water balance budgets should be interpreted with caution, given the fact that the calculation method does not incorporate basic hydrologic properties and is intended to give basic plot and landscape level estimates.

# References

1. FAO, *CROPWAT 8.0: A computer program for irrigation planning and management.* Irrigation and Drainage Paper, 2012. **46**.

2. Smith, M., *CROPWAT: Manual and guidelines.* FAO of UN, Rome, 1991.

3. Smith, M., *CROPWAT: A computer program for irrigation planning and management*. Vol. 46. 1992: Food & Agriculture Org.

4. Engel, B., et al., *Biofuels and water quality: challenges and opportunities for simulation modeling.* Biofuels, 2010. **1**(3): p. 463-477.

5. Williams, J.R. and V. Singh, *The EPIC model.* Computer models of watershed hydrology., 1995: p. 909-1000.

6. Arnold, J. and G. Soil, *SWAT (Soil and Water Assessment Tool)*. 1994: Grassland, Soil and Water Research Laboratory, USDA, Agricultural Research Service.

7. Gassman, P.W., et al., *Soil and Water Assessment Tool: Historical Development, Applications, and Future Research Directions, The.* 2007.

8. Vanuytrecht, E., et al., *AquaCrop: FAO's crop water productivity and yield response model.* Environmental Modelling & Software, 2014. **62**: p. 351-360.

9. Allen, R.G., et al., *FAO-56 dual crop coefficient method for estimating evaporation from soil and application extensions.* Journal of irrigation and drainage engineering, 2005. **131**(1): p. 2-13.

10. Team, R.C., *R: A language and environment for statistical computing.* R Foundation for Statistical Computing, Vienna, Austria., 2015.

11. Mu, Q., M. Zhao, and S. Running, *MODIS global terrestrial evapotranspiration (ET) product (NASA MOD16A2/A3) Algorithm Theoretical Basis Document Collection 5, College of Forestry and Conservation, University of Montana, Missoula.* MT. USA, 2013.

12. Mu, Q., M. Zhao, and S.W. Running, *Improvements to a MODIS global terrestrial evapotranspiration algorithm.* Remote Sensing of Environment, 2011. **115**(8): p. 1781-1800.

13. Craig, M. *The NASS cropland data layer program*. in *Third International Conference on Geospatial Information in Agriculture and Forestry, Denver, Colorado*. 2001.

14. Boryan, C., et al., *Monitoring US agriculture: the US department of agriculture, national agricultural statistics service, cropland data layer program.* Geocarto International, 2011. **26**(5): p. 341-358.

15. Teter, J., *CropWatR version 1.0.* 2015.

16. Di Luzio, M., et al., *Constructing retrospective gridded daily precipitation and temperature datasets for the conterminous United States.* Journal of Applied Meteorology and Climatology, 2008. **47**(2): p. 475-497.

17. Thornton, P.E., H. Hasenauer, and M.A. White, *Simultaneous estimation of daily solar radiation and humidity from observed temperature and precipitation: an application over complex terrain in Austria.* Agricultural and forest meteorology, 2000. **104**(4): p. 255-271.

18. Thornton, P.E. and S.W. Running, *An improved algorithm for estimating incident daily solar radiation from measurements of temperature, humidity, and precipitation.* Agricultural and Forest Meteorology, 1999. **93**(4): p. 211-228.

19. Thornton, P.E., S.W. Running, and M.A. White, *Generating surfaces of daily meteorological variables over large regions of complex terrain.* Journal of Hydrology, 1997. **190**(3): p. 214-251.

20. RDA, *Dataset number 512.0.* The Research Data Archive (RDA) is maintained by the Computational and Information Systems Laboratory (CISL) at the National Center for Atmospheric Research (NCAR). NCAR is sponsored by the National Science Foundation (NSF). accessed 2014.

21. USDA, *U.S. General Soil Map (STATSGO2).* Soil Survey Staff, Natural Resources Conservation Service, United States Department of Agriculture., Accessed 6/9/2013.

22. NASS, U., *Farm and ranch irrigation survey.* US Department of Agriculture, National Agricultural Statistics Service, 2008.

23. Allen, R.G., et al., *Crop evapotranspiration-Guidelines for computing crop water requirements-FAO Irrigation and drainage paper 56.* FAO, Rome, 1998. **300**: p. 6541.

24. Connor, D.J., R.S. Loomis, and K.G. Cassman, *Crop ecology: productivity and management in agricultural systems*. 2011: Cambridge University Press.

25. Eichinger, W., et al., *MEASURING EVAPOTRANSPIRATION AND VIRTUAL POTENTIAL HEAT FLUX USING LIDAR.* 2006.

26. Sentelhas, P.C., T.J. Gillespie, and E.A. Santos, *Evaluation of FAO Penman–Monteith and alternative methods for estimating reference evapotranspiration with missing data in Southern Ontario, Canada.* Agricultural Water Management, 2010. **97**(5): p. 635-644.

27. Tabari, H., M.E. Grismer, and S. Trajkovic, *Comparative analysis of 31 reference evapotranspiration methods under humid conditions.* Irrigation Science, 2013. **31**(2): p. 107-117.

28. Gesch, D., et al., *New products from the shuttle radar topography mission.* Eos, Transactions American Geophysical Union, 2006. **87**(18): p. 174-174.

29. Stern, A.J., P.C. Doraiswamy, and E.R. Hunt, *Changes of crop rotation in Iowa determined from the United States Department of Agriculture, National Agricultural Statistics Service cropland data layer product.* Journal of Applied Remote Sensing, 2012. **6**(1): p. 063590-063590.

30. Han, W., et al., *CropScape: A Web service based application for exploring and disseminating US conterminous geospatial cropland data products for decision support.* Computers and Electronics in Agriculture, 2012. **84**: p. 111-123.

31. Boryan, C., et al., *A New Automatic Stratification Method for US Agricultural Area Sampling Frame Construction Based on the Cropland Data Layer.* 2014.

# Appendix

The appendix is organized into the following sections:

1. Calculating Reference Evapotranspiration
2. The NASS Cropland Data Layer
3. Soil Texture
4. Calibration and Validation Results
5. Overview of Calculations

## I. Calculating Reference Evapotranspiration

The general Penman-Monteith equation is: $\lambda ET= \frac{\Delta\left( R_{n}-G \right)+\rho_{a}c_{p}\frac{e_{s}}{e_{a}}}{\Delta+\gamma(1+\frac{r_{s}}{r_{a}})}$

From this general formulation, the FAO derives the following daily time step implementation (also known as FAO Penman-Monteith):

$${ET}_{0}= \frac{0.408\Delta\left( R_{n}-G \right)+\gamma\frac{900}{T+273}u_{2}({e_{s}-e}_{a})}{\Delta+\gamma(1+{0.34u}_{2})}$$

The FAO document 56 recommends this FAO Penman-Monteith method over all other methods for estimating reference evapotranspiration. Recent studies [26, 27] confirm the superior performance of FAO Penman-Monteith as compared with other methods, including Priestley-Taylor, Hargreaves, and Thornthwaite, provided complete data (and in particular net incident radiation (Rn) data) are available. The sole drawback of FAO Penman-Monteith is the requirement that all the data are available and are measured with a high degree of accuracy and precision. This was the rationale for importing data from multiple sources, most of which have been extensively validated, improved over multiple cycles, and are hence authoritative data. Data sources include USGS [28]; PRISM [16]; DAYMET [17-19]; and the RDA dataset number 512.0 [20].

***ET_o_*** is the reference evapotranspiration in mm per day, as derived from the following variables:

***R_n_***, net radiation, is taken from DAYMET: *SRAD* – daylight average incident shortwave radiation. DAYMET generates daily observations as a function of Sun-slope geometry and interpolated diurnal temperature range. Units were converted from watts to MJ per square meter per day.

***G*** is the soil heat flux in MJ per square meter per day, which is taken to be zero in calculations at a daily time step.

***T*** is the daily air temperature at 2 meters height in degrees Celsius. Daily minimum, average, and maximum temperature were all taken from PRISM, and are all used in the derivation of ***ET_o_***.

***u_2_*** is average daily wind speed at 2 meters height. It is calculated from observations taken at 10 meters height from the RDA dataset number 512.0. 

***e_s_*** is the saturation vapor pressure (0.622 kPa), and ***e_a_*** is the actual vapor pressure. Δ is the slope of the vapor pressure curve in kPa per degrees Celsius. ***e_s_*** minus ***e_a_*** is the saturation vapor pressure deficit. Actual daily vapor pressure was calculated both as a function of minimum and maximum daily relative humidity (interpolated via TPS from the RDA 512.0 data), and taken directly from DAYMET estimated vapor pressure in kPa.

***γ***is the psychrometric constant, measured in kPa per degrees Celsius.

All parameters and physical constants are explained in detail in the FAO 56 documentation [9].

All parameters were derived at a daily time step at approximately 10 x 10 km resolution. S2 Fig I summarizes the key input parameters that are used in the calculation of daily ET_o_ per Penman-Monteith. These are maps shown the annual aggregated summary statistics (means or sums), from top left to bottom right, of (1) mean daily maximum temperature (°C) (2) mean daily minimum temperature (°C) (3) mean daily maximum relative humidity (%) (4) mean daily minimum relative humidity (%) (5) total annual precipitation (mm) (6) mean wind speed (m/s at 3 meters height) (7) total incident solar radiation (kW per square meter) (8) derived reference evapotranspiration (ET_o_) (mm).

The scripts “*DayMet.R*”, “*PRISM.R*”, “*Soil.R*”, and “*TPS.R*”, among others (for the complete listing and descriptions, see appendix section V) download and process input data. The script “*ETo.Calculation.R*” derives a raster ET_o_ given raster input files according to the FAO Penman-Monteith equation at a daily time step.

S2 Fig I.

## II. The NASS Cropland Data Layer

The Cropland Data Layer (CDL), also known as CropScape, is the USDA National Agricultural Statistical Service’s online GIS data portal that compiles annual cropland and land use data as ‘censused by satellite’ and ground trothed by actual survey sampling. The CDL collects these data in a user-friendly format for visualization, summary and statistical analysis, and download in tabular and raster format. The CDL has undergone many cycles of revision, validation, and methodological improvement since its inception in 1997. More details on the CDL, including an overview of its history, verification exercises, and analysis using the CDL, are available in numerous articles [14, 29-31].

S2 Table E shows the reclassification from CDL categories to the ones used in this illustration and test of the modeling tool developed here.

S2 Table E. NASS Classification categories considered in this analysis.

| NASS Code | NASS classification | Reclassification |  | NASS Code | NASS classification | Reclassification |
| --- | --- | --- | --- | --- | --- | --- |
| 1 | Corn | Corn |  | 45 | Sugarcane | Sugarcane |
| 2 | Cotton | Cotton |  | 225 | Dbl. Crop WinWht/Corn | Dbl. Crop WinWht/Corn |
| 3 | Rice | Rice |  | 226 | Dbl. Crop Oats/Corn | Dbl. Crop Oats/Corn |
| 4 | Sorghum | Sorghum |  | 234 | Dbl. Crop Durum Wht/Sorghum | Dbl. Crop Durum Wht/Sorghum |
| 5 | Soybeans | Soybeans |  | 235 | Dbl. Crop Barley/Sorghum | Dbl. Crop Barley/Sorghum |
| 10 | Peanuts | Peanuts |  | 236 | Dbl. Crop WinWht/Sorghum | Dbl. Crop WinWht/Sorghum |
| 21 | Barley | Barley |  | 237 | Dbl. Crop Barley/Corn | Dbl. Crop Barley/Corn |
| 22 | Durum Wheat | Durum Wheat |  | 238 | Dbl. Crop WinWht/Cotton | Dbl. Crop WinWht/Cotton |
| 23 | Spring Wheat | Spring Wheat |  | 239 | Dbl. Crop Soybeans/Cotton | Dbl. Crop Soybeans/Cotton |
| 24 | Winter Wheat | Winter Wheat |  | 240 | Dbl. Crop Soybeans/Oats | Dbl. Crop Soybeans/Oats |
| 26 | Dbl. Crop WinWht/Soy | Dbl. Crop WinWht/Soy |  | 241 | Dbl. Crop Corn/Soybeans | Dbl. Crop Corn/Soybeans |
| 28 | Oats | Oats |  | 254 | Dbl. Crop Barley/Soybeans | Dbl. Crop Barley/Soybeans |
| 36 | Alfalfa/hay | Alfalfa/hay |  | *61* | *Fallow/Idle Cropland* | *Fallow/Idle Cropland* |
| 37 | Other Hay | Alfalfa/hay |  | *171* | *Grassland Herbaceous* | *Grassland_pasture* |
| 41 | Sugarbeets | Sugarbeets |  | *181* | *Pasture/Hay* | *Grassland_pasture / Alfalfa/hay* |

Note that double-cropped classifications where both crops were not included among the 14 parameterized crops (e.g. lettuce / upland cotton, lettuce / barley, etc.) were excluded from the analysis. The total acreage of these classifications on the national scale was in all cases much less than 1% of the acreage of the modeled crop.

S2 Table F shows national accuracy statistics for the 2008 CDL for the crops and land types modeled in this exercise. Generally speaking, the higher the crop coverage in a given state, the higher the accuracy. State-by-state breakdowns of these statistics are available upon request, or can be obtained directly from NASS at the following urls:

Spatial data - <http://www.nass.usda.gov/research/Cropland/metadata/meta.htm>.

Summary tables - <http://www.nass.usda.gov/research/Cropland/sarsfaqs2.html#Section1_12.0>

*Producer’s accuracy* reports the probability of “errors of omission” – i.e. that a pixel is *excluded* from the *correct* category. User’s accuracy refers to the probability of “errors of commission” – i.e. that a pixel is *included* in an *incorrect* category. The *Kappa Coefficient* is a statistical measures of non-chance agreement between two maps (here, between the output CDL classifications and a ground truth map).

*Total pixels* indicates the number of (30 or 50 meter) pixels in the 2008 CDL designated under the specified crop or land use category.

S2 Table F. NASS Accuracy assessments for crops and land types considered in this analysis.

| **Crop** | **User** | **Commission** | **Kappa** | **Total pixels** |
| --- | --- | --- | --- | --- |
| Corn | 0.95 | 0.05 | 0.91 | 14,732,113 |
| Cotton | 0.88 | 0.12 | 0.85 | 1,096,608 |
| Rice | 0.96 | 0.04 | 0.95 | 819,200 |
| Sorghum | 0.79 | 0.21 | 0.76 | 710,375 |
| Soybeans | 0.94 | 0.06 | 0.91 | 11,643,167 |
| Peanuts | 0.77 | 0.23 | 0.62 | 109,602 |
| Barley | 0.78 | 0.22 | 0.75 | 945,837 |
| Durum Wheat | 0.72 | 0.28 | 0.68 | 695,813 |
| Spring Wheat | 0.80 | 0.20 | 0.72 | 3,757,894 |
| Winter Wheat | 0.90 | 0.10 | 0.79 | 8,404,641 |
| Dbl. Crop WinWht / Soy | 0.86 | 0.14 | 0.85 | 838,259 |
| Oats | 0.53 | 0.47 | 0.52 | 136,798 |
| Sugarbeets | 0.96 | 0.04 | 0.96 | 147,414 |
| Dbl. Crop WinWht / Corn | 0.63 | 0.37 | 0.62 | 21,057 |
| Dbl. Crop Oats / Corn | 0.72 | 0.28 | 0.71 | 38,026 |
| Dbl. Crop Durum Wht / Sorghum | 0.90 | 0.10 | 0.90 | 7,711 |
| Dbl. Crop WinWht / Sorghum | 0.25 | 0.75 | 0.25 | 1,090 |
| Dbl. Crop Barley / Corn | 0.95 | 0.05 | 0.95 | 56 |
| Dbl. Crop WinWht / Cotton | 0.30 | 0.70 | 0.30 | 462 |
| Dbl. Crop Soybeans / Oats | 0.40 | 0.60 | 0.40 | 363 |

Accuracy statistics for crop and land use categories not reported in the above table are not available for 2008. These categories are: alfalfa, other hay, sugarcane, fallow/idle cropland, grassland herbaceous, and pasture/hay.

The script “*Aggregate.Classify.CDL*” downloads, reclassifies, and aggregates cropping locations and acreage to the 10 by 10 km resolution. “*Perspective.Plot.R*” creates the plots in S2 Fig J.

S2 Fig J shows the density of coverage of crops and other land cover classifications modeled in 2008, according to the reclassification and downscaled to a resolution of approximately 10 x 10 km. All maps are shown in the same scale.

S2 Fig J.

## III. Soil Texture

S2 Fig K shows the derived classification of soil type for soils of at least one meter depth, according to the USDA soil pyramid. Data were extracted from STATSGO2 [21], and the classification was done according to the percentage of silt, sand and clay in this subset of soils (i.e. those with a minimum depth of one meter). Missing pixels were interpolated by inverse distance weighting (IDW).

S2 Fig K.

The script “*Soil.Texture.R*” downloads, aggregates, and classifies the STATSGO2 soil texture data.

## IV. Calibration and Validation Results

S2 Fig L shows the irrigation calibration results. Maps and density plots are given for the three main row crops other than corn (soybeans, alfalfa, and winter wheat), and density plots of statewide mean irrigation intensity (in acre-feet per acre) are shown for irrigated varieties. S2 Fig M shows the locations of the validation runs for each crop against the MODIS 16 data product.

S2 Fig L.

S2 Fig M.

S2 Fig N.

## V. Overview of Calculations

This section provides a detailed outline of the calculations and considerations discussed in FAO 56 [9] and implemented in *CropWatR*. It provides a conceptual overview of the model, as well as references to scripts and functions and their corresponding discussion in the FAO 56 document. It outlines each of the following functionality of the model:

1. Downloading and processing GIS input data
2. Deriving ET_o_
3. Water balance calculations (dual crop coefficient, soil evaporation coefficient, irrigation & water stress, daily soil water balances, and non-cropland, & weed/volunteer [off-season] ET estimation)
4. Processing, aggregating, and visualizing outputs

Throughout this overview, **functions** are highlighted in bold, *scripts* are in italics, and equations directly referenced from FAO 56 are underlined.

#### V.1. Downloading and processing GIS input data

The following scripts were used to download and preprocess (including reclassifying, reprojecting, masking, and aggregating to the 10 by 10 km resolution) the input raster data:

*Elevation.R* – Downloads and processes elevation data from the USGS.

*DayMet.R* – Downloads and processes DayMet inputs (temperature, solar radiation, vapor pressure).

*PRISM.R* – Downloads and processes PRISM inputs (min, mean, and max temperature; precipitation).

*Soil.Texture.R – Downloads and processes the STATSGO2 soil texture data.*

*Soil.R – sourced by Soil.Texture.R; soilGo function processes soil data (from the ‘dirt’ package by Robert Hijmans).*

*Aggregate.Classify.CDL* – Downloads and processes crop locations from the Cropland Data Layer, outputs a raster file with layers reporting the acreage in each 10 by 10 km grid cell of crops of interest.

*MODIS.16.R* – Downloads & processes MODIS16 data (requires external software: gdal drivers, MRT).

*NCAR.R* – Downloads and processes weather station data (min and max relative humidity, wind speed, and other variables not used in the test) (requires external software: Fortran).

*Elevation.Station.Check.R* – checks the location (lat/long) data of NCAR weather stations based on reported elevation and corrected misidentified coordinates (i.e. those specified as 1-60 rather than decimal values).

*TPS.R* – applies the thin-plate splines function Tps() from the *R* package fields to interpolate relative minimum and maximum humidity and wind speeds based on point (station) data.

*Match.Raster.R* – Reprojects and masks raster files to match a template.

#### V.2. Deriving ET_o_

Section I of this appendix outlines the calculations used to derive reference evapotranspiration. The script “*ETo.Calculation.R*” derives a raster ET_o_ given raster input files according to the FAO Penman-Monteith equation at a daily time step.

#### V.3. Water balance calculations

Chapters 5-7 of the FAO 56 report describe the qualitative considerations, crop-, soil-, and management-specific parameters, and calculation algorithms to estimate crop evapotranspiration (ET_c_) using a single (K_c_) or dual (K_cb_ + K_e_) crop coefficient. The model developed uses the dual crop coefficient approach (described in chapter 6 of the report), as this allows the user to estimate the evaporation and transpiration components of ET separately, although the user should note that the evaporation component is typically overestimated and the evaporation component underestimated, as the former “include[s] a residual diffusive component supplied by soil water below the dry surface and by soil water from beneath dense vegetation” [9]. Chapter 8 of FAO 56 describes the computations and parameters used to model ET_c_ under soil water stress conditions, as well as simple rules to model irrigation scheduling. Chapter 9 describes approaches for estimating ET_c_ for “natural, non-typical, and non-pristine” vegetation types, and chapter 11 for non-growing periods such as bare soil, weed growth during the fallow season, and frozen or snow covered surfaces. All of the computation algorithms described in chapters 6-9 are used in the model, as are simplified versions of the calculations outlined in chapters 9 and 11. What follows is a brief description of the main parameters and computations implemented in the model.

##### Dual crop coefficient (K_cb_)

Based on the user input planting and harvesting dates, the year is first split into a fallow season and a growing season. The initial, mid, and end-stage basal crop coefficients (K_cb_) given in Table 17 of FAO 56 are rescaled according to the relative length of the appropriate region-specific crop development stages (Table 11 of FAO 56) to match the length of the growing season, and from this a curve is fit (as in Figure 37 of FAO 56) to interpolate daily K_cb_ values, mean rooting depth (Table 22), and plant height (Table 12). A Bezier curve is fit to the parameters outlined in the FAO 56 document, so that instead of level and linear changes in rooting depth, crop coefficients (K_cb_ values), and plant heights – as instructed in the FAO 56 report –, a continuous growth curve is fit to each of these functions. The functions in *WB.functions.R* under the heading “Subset Growth Season” execute this split. The functions are: **Split.Seasons**, **Rescale.And.Save**, **Save.Crops.List**, and **Rescale.Season, bezierCurve** and **bez**, and **Daily.Crop.Curves**. All of these functions are called by the wrapper and master function, **Subset.Growth.Season.** Further details on these functions can be found in comments in the source code.

The basal dual crop coefficient is the basis for calculations estimating the actual crop coefficient in extreme weather conditions and for estimating the percentage of soil surface effectively covered by vegetation. These functions performing these calcuations are in the script *WB.functions.R* under the heading “Daily Crop Parameters.” The K_cb_ values in the mid and late stages are then adjusted in climates where the minimum relative humidity differs from 45% or where the wind speed is larger or smaller than 2 m/s as per equation 70 of FAO 56 (this is implemented in the function **Calc.Basal.Crop.Coeff**).

The remaining calculations proceed in the order designated in chapter 7 of FAO: deriving the daily upper limit evapotranspiration (K_c max_) as well as the minimum K_c_ for dry bare soil with no ground cover, following equation 72 (**KcMAX** and **KcMAX.fallow**). Next, the functions **Calc.Fc.Few** and **Fallow.Few.Calc** execute equations 75 and 76, using the outputs of the above calcuations and K_cb_ to estimate the daily soil fraction shaded by vegetation (f_c_) and its complement, the exposed soil fraction (1- f_c_). The wrapper function **Daily.Crop.Parameters** executes all the above functions in “Daily Crop Parameters.”

##### Daily water balance calculations

The remainder of the calculations require iterative computation, and thus are all computed in the third subsection of the *WB.functions.R* script: “Daily ET Calcs.” An explanation the sequence of the computations performed in the functions of that subsection follows a brief discussion of the conceptual considerations outlined in FAO 56.

##### Soil evaporation coefficient (K_e_)

estimating the fraction of the soil surface that is wetted by irrigation or precipitation (f_w_) – which depends also on whether the crop has triggered irrigation rules in the previous day – and finally deriving a daily estimate of the exposed and wetted soil fraction (f_ew_) from these parameters. Next the evaporation reduction coefficient (K_r_), which is a measure of the proportion of the water depleted by evaporation from the topsoil, is calculated based on the previous day’s cumulative depth of evaporation, and total evaporable water (TEW) and readily evaporable water (REW), which themselves are derived based on three parameters: (1) soil water content at field capacity (q_FC_) and (2) at wilting point (q_WP_), and (3) the depth of the soil surface layer that is subject to evaporative drying (Z_e_). These three parameters vary depending on soil texture.

The above parameters (K_cb_, F_ew_, and F_c_) are used to derive the daily soil evaporation coefficient (K_e_) – which is dependent upon the exposed and wetted soil fraction, the maximum value of crop-specific ET following a rain or irrigation event (K_c max_), and the evaporation reduction coefficient (K_r_). The above parameters are used to estimate the (day-end) deep percolation loss from the topsoil (DP_e_) and the cumulative depth of soil evaporation (D_e_) for calculation of the subsequent day’s soil-water balance. The cumulative depth of soil evaporation (D_e_) is bounded by zero (immediately following sufficiently heavy rainfall or irrigation) and the total evaporable water (TEW). Finally, daily evaporation is then calculated as the product of K_e_ and the reference evapotranspiration (ET_o_), and daily transpiration is estimated by the product of K_cb_ and ET_o_.

It should be clear from the above summary of computations that the soil-water balance calculations are dependent on precipitation, weather, the modeled plant growth, and irrigation over the previous day(s), and hence that the calculation of daily crop-water balances is necessarily iterative, proceeding indeed not only from the first day of the growing season but, as implemented in this model, from the initial day of the preseason, that is, from immediately after the (modeled) harvest of the proceeding year.

##### Irrigation and water stress impacts

Irrigation is triggered when the water stress coefficient (K_s_) falls below some crop-specific threshold (this is true for all crops except rice, where irrigation is triggered when readily available water – RAW – drops below 0.56). Under soil water limited conditions, K_s_ falls below 1, and when there is no water stress, K_s_ equals 1. Water stress is used as a scalar to estimate the compromised metabolic capacity and corresponding in the capacity of a crop to transpire under water stress, as such it results in a reduction in the daily ET_c_.

Water stress calculations are the topic of chapter 8 in FAO 56. All calculations and parameters in that chapter are incorporated in the model. What follows is a brief overview of the computations; for more details on any of the computations, the reader is referred to the original FAO 56 report, which is free and easily accessible online. As with TEW and REW, total available water (TAW) and readily available water (RAW) are derived properties that vary according to soil composition. Both are indicators of the capacity of soils to retain water that is available to plants in the root zone. RAW is derived from TAW and a crop-specific soil depletion fraction for no stress (p), which is the average fraction of TAW that can be depleted from the root zone below the plant experiences moisture stress and a resultant reduction in ET. This crop specific multiplier is modeled as a function of crop specific evapotranspiration (ET_c_) at ET_c_ values of less than 5 mm/day.

##### Daily soil water balances

Daily root zone soil water depletion (D_r_) is determined as the remaining soil water leftover from the previous day’s depletion minus the sum of the day’s precipitation plus net irrigation that infiltrates the soil root zone, less runoff, crop evapotranspiration, and water loss due to deep percolation (groundwater infiltration). Daily root zone soil depletion (D_r_) is hence bounded between 0 and the TAW, and initial depletion is set at the beginning of the simulation (i.e. immediately following a hypothetical harvest from the previous season, or equivalently, at the beginning of the previous year’s fallow season) at 25% of the TAW. That is, at the end of the previous year’s growing season, 25% of TAW is assumed to be depleted. Results are nearly completely insensitive to initial assumptions into the level of root zone soil depletion. When soil water content in the root zone is at field capacity, daily deep percolation (DP), or groundwater recharge, is calculated as the remainder of water not allocated in the previous calculations, i.e. as the next sum of precipitation and irrigation minus the net sum of runoff, evapotranspiration, and the previous day’s soil water depletion (equation 88 in FAO 56). As outlined in FAO 56, capillary rise is assumed to be zero as it is very close to zero when the water table is more than 1 meter below the root zone. Also outlined in chapter 8 are computations enabling the estimation of the effects of soil salinity on crop growth and crop-water balances, the reduction in yield due to moisture stress, and the interactive effects of salinity and moisture stress. None of these have yet been incorporated into the model as they were not needed for the author’s current research, but all could easily be added.

##### Non-cropland ET estimation

Chapter 9 of the FAO 56 report outlines methods for estimating single- and dual (K_cb_) crop coefficients for natural, non-typical, and non-pristine vegetation. It outlines methods for adjusting K_cb_ values in the initial, mid, and late seasons under conditions of sparse vegetation and for deriving and calibrating K_cb_ based on leaf area index (LAI) measurements, effective ground cover, and the ratio between actual and potential crop yields. The recommendations in this chapter were used to parameterize K_cb_ values, plant heights, and rooting depths for perennial vegetation. Specifically, given the low accuracy in distinguishing among fallow / idle cropland, pasture grass, and grassland herbaceous land covers in the CDL, similar K_cb_ values in the initial, mid, and late season; crop heights; and rooting depths have been assumed for all of these land covers nationwide. Note that this does not imply that nationwide heights, rooting depths, and growing seasons were uniform for these land cover classifications; rather, the growing season and potential transpiration and growth of perennials is sensitive to weather and soil parameters that vary in space and time.

##### Fallow season ET estimation

Estimation of ET_c_ during non-growing periods is the topic of chapter 11 of FAO 56. As advised in this chapter, when temperatures fall below zero for sustained periods of time, the daily water balance calculations are implemented assuming that K_cb_ equals 0, otherwise low K_cb_ values have been selected to represent weed- or volunteer plant growth based on the recommendations in chapter 9. Specifically, an initial K_cb_ value of 0.25, root depth of 0.1 meters, and p value of 0.1 (appropriate for shallow-rooted weeds) is adopted and these values increase daily – the K_cb_ value increases by 0.005 and the root depth by 0.002 per day. As noted in chapter 11, since the soil water balances automatically adhere to the conservation of mass in the K_cb_ calculations, there is no risk in overestimating actual weed/volunteer K_cb_. At worst, the fraction of evapotranspiration represented by weeds (and not by evaporation) will be over- or underestimated. No further adjustments have been made to calculate water-balances for frozen or snow covered surfaces, as the reference evapotranspiration (ET_o_) approach has been shown to perform inadequately under such conditions. The fraction of off-season water balances that will be inaccurately estimated as a result of failure to incorporate frozen or snow covered soils will clearly increase at higher latitudes and regions with colder winters, but given the fact that most changes of interest to researchers modeling crop-water balances, such as changing cropping and land use patterns, have little to no direct impact on soil conditions under sustained sub-zero temperatures, correctly modeling such conditions is not a priority and does not significantly effect the fidelity of the overall model given the current advised applications.

##### Daily computations

The functions listed below are called by the master function **Final.Daily.ET.Calc** and perform the calculations of water flows at a daily time step for a list of data.frames (written and saved by the previous functions) for all locations and crop management parameters for a given crop. Each of the functions computes the same general set of calculations, but with slightly differing assumed parameter values (e.g. Kcb, root growth, irrigation timing, etc.) as appropriate to the season (i.e. for the fallow preseason and postseason, and for the growing season under rainfed and irrigated conditions), given the above discussion:

**Fallow.Preseason.Daily.ET.Calc**– calculates water balances for the preseason

**Main.Growing.Season.Daily.ET.Calc**– calculates water balances for the crop growing season; applies irrigation rules

**Main.Rainfed.Growing.Season.Daily.ET.Calc**– calculates water balances for crop growing season; assumes rainfed cultivation

**Fallow.Postseason.Daily.ET.Calc**– calculates water balances for the postseason

The computations **Main.Growing.Season.Daily.ET.Calc**, which performs calculations for an irrigated crop, is taken as an example for illustrating the basic workings of all of the above functions, and then the assumptions and alterations of the other functions are explained.

Each daily water balance function first loads outputs of the previous sets of functions: lists of data.frames containing a matrix of crop-specific (1) daily ET_o_ [mm] (2) precipitation [mm] and (3) soil texture data for each location (grid cell), as well as crop-specific daily parameters (3) max height and (4) root depth, (5) daily K_cb_ values, (6) effective ground cover (Few). From these lists of data.frames (where each data.frame represents a cluster of crops modeled as having growing seasons of the same length), new lists of data.frames are initialized with the same dimensions, to represent the following parameters, which are estimated in the order listed below, as per the FAO 56 algorithm (see “order of calculation” in Chapter 7), as outlined conceptually above, and in detail in FAO 56 chapters 7-9. In each case, *i* is a daily subscript:

##### Base calculations (from FAO Chapter 7)

Exposed and wetted soil fraction (*F_ew,i_*)

(1) $f_{ew,i}=min(f_{ew,i}, f_{w,i})$ where $0<F_{ew,i}<1$ Equation 75

The evaporation reduction coefficient (*K_r,i_*)

(2) $K_{r,i}= \frac{TEW-D_{e, i-1}}{TEW-REW}$where $D_{e, i-1}>REW$ and $0<K_{r,i}<1$ Equation 74

The soil evaporation coefficient (*K_e,i_*)

(3) $K_{e,i}=min(K_{r,i}\left( K_{c max,i}-K_{cb,i} \right), {f_{ew,i} K}_{c max,i})$ where $K_{e,i}>0$ Equation 71

Evaporation (*E_i_*)

(4) $E_{i}= K_{e,i}{ET}_{o,i}$ (evaporation on day *i*, where the fraction $\frac{E_{i}}{f_{ew,i}}$ provides for the actual concentration of the evaporation over the fraction of the soil that is both exposed and wetted)

Topsoil deep percolation loss (*DP_e,i_*)

(5) ${DP}_{e,i}=\left( P_{i}-{RO}_{i} \right)+ \frac{I_{i}}{f_{w}}-D_{e,i} \geq0$ Equation 79

In cases where the soil water content in the evaporation layer is below field capacity (i.e. *D_e,i_* > 0), the soil will not drain and hence *DP_e,i_* > 0.

Crop evapotranspiration (*ET_c,i_*)

(6) ${ET}_{c,i}=\left( K_{cb,i}+K_{e,i}{ET}_{o,i} \right)$ Equation 69

##### Water stress calculations (from Chapter 8)

P value (*p_i_*), which is a function of *p*, a crop-specific parameter (taken from FAO Table 22) representing the average fraction of TAW that can be depleted from the root zone before moisture stress (reduction in ET) occurs, and *ET_c_*.

(7)$p_{i}=p_{Table 22}+(5-{ET}_{c,i})$

Daily root depth (*Z_r,i_*)

(8)$Z_{r,i}=\frac{Z_{r,i}}{Z_{r Max}}$

Total available water (*TAW_i_*) [mm]

(9) ${TAW}_{i}=1000\left( q_{FC}-q_{WP} \right)Z_{r,i}$ Equation 82

Readily available water (*RAW_i_*) [mm]

(10) ${RAW}_{i}=p_{i} \times{TAW}_{i}$ Equation 83

Water stress coefficient (*K_s_*) (pre-irrigation value)

(11) $K_{s,i}=\frac{{TAW}_{i}-D_{r,i}}{{TAW}_{i}-{RAW}_{i}}$ where $0{<K}_{s,i}<1$ Equation 84

##### Application of irrigation (from Chapter 8)

Irrigation depth (*I_i_*)

(12)$I_{i}={RAW}_{i}\times I_{Crop}$ if $K_{s,i}>T$, where T is a crop-specific threshold

Irrigation rules apply a given (calibrated and crop-specific) fraction of *RAW_i_* once a certain threshold of water stress (*K_s,i_*) is exceeded. The fraction of irrigation water applied was calibrated to approximate the average irrigation water intensity across all survey units (U.S. states, in the application shown in this article), and then multiplied by scalars representing the unit-specific deviation from this average value.

Water stress coefficient (*K_s_*) (post-irrigation value)

(13) $K_{s,i}=\frac{{TAW}_{i}-D_{r,i}}{{TAW}_{i}-{RAW}_{i}}$ where $0{<K}_{s,i}<1$ Equation 84

Adjusted (post-irrigation) crop transpiration

(14) ${ET}_{c\_adj,i}=(K_{s,i}K_{cb,i}+K_{e,i}){ET}_{o,i}$ Equation 80

Soil water balance in the root zone (*D_r,i_*)

(15) $D_{r,i}=D_{r,i-1}\left( P_{i}-{RO}_{i} \right)-I_{i}+{ET}_{c_{adj},i}+{DP}_{i}$ where $0<D_{r,i}<{TAW}_{i}$ Eqs. 85 and 86

and where *P_i_* is precipitation on day *i* and *RO_i_* is runoff on day *i*.

Deep percolation (groundwater infiltration) (*DP_i_*)

(16) ${DP}_{i}=\left( P_{i}-{RO}_{i} \right)+I_{i}-{ET}_{c_{adj},i}-D_{r,i-1}$ Equation 88

where if the soil water content is below field capacity (i.e. *D_r,i_* > 0), soil does not drain & *D_pi_* = 0

Soil water balance – cumulative depth of evaporation (*D_e,i_*) [mm]

(17)$D_{e,i}=D_{e,i-1}-\left( P_{i}-{RO}_{i} \right)-\frac{I_{i}}{f_{w,i}}+\frac{E_{i}}{f_{ew,i}}-TEW+{DP}_{e,i}$ Equation 77

subject to:

(18) ${0<D}_{e,i}<TEW$ Equation 78

Where *TEW* is a parameter that is a function of soil texture – see Equation 73 and Table 19 in FAO 56, as well as the script *Soil.Texture.R*.

Having computed each of the above calculations for each day of the growing season in succession, the function saves the values of each of the following parameters for each day, in each grid cell (as separate lists of data.frames, which are merged and saved as raster outputs – see the following section):

- *K_r_* (Evaporation reduction coefficient) 0 < *K_r_* < 1
- *K_s_* (Water stress coefficient) 0 < *K_s_* < 1
- *P_i_-value* (Crop specific and *K_cb,i_* dependent coefficient) 0 < *P_i_* < 1
- *D_r_* (root zone depletion) [mm]
- *D_e_* (soil water balance) [mm]
- *DP* (deep percolation) [mm]
- *RO* (runoff) [mm]
- *E* (soil evaporation) [mm]
- *I* (irrigation) [mm]
- Transpiration [mm]

All of the daily water balance calculations except for the preseason files load the following crop-specific output files from the previous time period (i.e. the growing season function loads preseason output files and the postseason function loads outputs from the growing season function) as a basis for using appropriate inputs from the previous day *i*:

- Deep percolation (i.e. groundwater infiltration – abbreviated as *DP* in FAO 56)
- Root zone depletion (*D_r_*)
- Runoff (*RO*)
- Soil Evaporation (*E*)
- Water Balance (or cumulative depth of evaporation – *D_e_*)
- Exposed and wetted soil fraction (*F_ew_*)

The following assumptions regarding weed growth seem to fit MODIS 16 ET estimates best (although mismatches are indeed most notable in the preseason and postseason, as might be expected given the generalized and non-mechanistic assumptions adopted in modeling these periods):

Preseason *K_cb_* is initialized at 0.35 and increases by an increment of 0.005 daily, the p-value is initialized at 0.1 (appropriate, according to FAO 56 chapter 9, for ‘shallow-rooted weeds’) and grows by an increment of 0.02 × (5 – *K_cb_*) daily (bounded by 0.1 and 0.8), and rooting depth begins at 0.1 meters and increases 0.002 (i.e. 2 mm) per day.

Postseason *K_cb_* is initialized at 0.75 and decreases by an increment of 0.003 daily, the p-value is initialized at 0.1 and grows by an increment of 0.02 × (5 – *K_cb_*) daily (bounded as with the preseason), and rooting depth begins at 0.1 meters and increases 0.002 (i.e. 2 mm) per day.

#### V.4 Processing, calibrating, and visualizing raster outputs

##### Saving raster outputs

Finally, the list of data.frames output by the above functions is recombined into a single data.frame, and this is converted to a raster format. The functions in the “Save Raster Outputs” subsection of the *Products.R* script create the following various output types:

**Sum.Save.Water.Balances** – sums estimated water balances over the entire year or over just the growing season, allowing user to specify the option of saving ‘blue’ (irrigation) and ‘green’ water (evaporation + transpiration) volumes.

**Sum.Save.Daily.Evapotranspiration** – creates a raster brick of daily water balances over the entire year.

**Generate.Land.Use.R –** generates acreage, by crop, according to the CDL.

**SuperImpose.WB.on.LU** – Superimposes **w**ater **b**alances on **l**and **u**se rasters to derive aggregate water use over land cropped in a given crop.

##### Calibrating irrigation intensity

**Plot.Check.Annual.Water.Balances** – For manually calibrating crop-specific irrigation rules based on survey reported irrigation intensity. Creates maps and distribution plots as in S2 Fig B and S2 Fig L.

##### Visualizing outputs

The following functions make use of the functionality of various R packages (e.g. RasterVis, ggplot2, to produce visualizations of raster output data.

*Perspective.Plotting.R* – creates perspective plots, as in S2 Fig J

*Plots.Maps.R* – Creates barplots, violinplots, and maps (e.g. Figures 3-5 in the article), as well as other graphics.

The following functions create the plots in the article:

**RasterVis.Or.Plot.Map.Water.Balances** – makes Figure 4

**Vioplot.Water.Balances** – makes Figure 5
