# Supplementary material for: Water impacts of U.S. biofuels: Insights from an assessment combining economic and biophysical models
Source: PLoS One. 2018 Sep 28;13(9):e0204298. doi: 10.1371/journal.pone.0204298 (PMC6161887; doi:10.1371/journal.pone.0204298)
Supplement: S2 File — Table A. Kcb parameter values for perennial grassland and non-crop agricultural land cover types. Table B. Crop water balances that can be estimated for daily, seasonal, or annual time steps. Table C. Literature estimates of evapotranspiration versus modeled evapotranspiration rates. Seasonal and annual evapotranspiration ranges reported in literature sources and model by CropWatR. A range of methods are available for determining evapotranspiration at the field, landscape, and watershed scales, either via direct measurement (e.g. soil moisture measurement via lysimeters), modeling (e.g. via process-based s imulation models, or energy balances using weather data collected via satellite, local instruments, and/or remote sensing). For a summary of common methods, see Connor et al.,2011. Table C. Model performance metrics comparing the results with MODIS 16 estimates. Table E. NASS Classification categories considered in this analysis. Note that double-cropped classifications where both crops were not included among the 14 parameterized crops (e.g. lettuce / upland cotton, lettuce / barley, etc.) were excluded from the analysis. The total acreage of these classifications on the national scale was in all cases much less than 1% of the acreage of the modeled crop. Table F. NASS Accuracy assessments for crops and land types considered in this analysis. Accuracy statistics for crop and land use categories not reported in the above table are not available for 2008. These categories are: alfalfa, other hay, sugarcane, fallow/idle cropland, grassland herbaceous, and pasture/hay. Fig A. Relation between time (day since emergence), crop coefficient (Kcb) and plant height for maize. Crop coefficients are specified for three moments in time. According to the FAO 56 methods, coefficients are estimated by stepwise and linear interpolation (black line). In CropWatR, a Bezier curve (red curve) is used to interpolate daily Kcb values. Fig B. Irrigation calibration results for corn. The [file pone.0204298.s002.zip › S2 File Figures Tables/S2 Table C.docx]

**S2 Table C. Literature estimates of evapotranspiration versus modeled evapotranspiration rates.**

| Source | Location | Crop | Method(s) | Period | Cited ET  (mm) (mean) | Modeled ET (mm) (mean) | Irrigated | Link |
| --- | --- | --- | --- | --- | --- | --- | --- | --- |
| Kanemasu, 1977a | Kansas | Corn | Measurements; simulation model | Growing season (May-August) | 500 | 410 - 590 (445) | Unspecified | [Kansas State](http://www.ksre.ksu.edu/historicpublications/Pubs/SRL32.pdf) |
| Hatfield & Prueger, 2003 | Central Iowa | Corn | Measurements | Annual | 290 - 580 (410) | 485 - 625 (550) | Both | [Link](http://cdn.intechopen.com/pdfs-wm/22687.pdf) |
| Hatfield & Prueger, 2003 | Central Iowa | Corn | Measurements | Growing season (April - Oct) | 275 - 500 | 400 - 450 (430) | Both | [Link](http://cdn.intechopen.com/pdfs-wm/22687.pdf) |
| Hatfield & Prueger, 2003 | Walnut Creek, Iowa | Corn | Measurements | Growing season (May-August) | 185 -275 | 380 - 445 (400) | Unspecified | [Link](http://cdn.intechopen.com/pdfs-wm/22687.pdf) |
| Hatfield & Prueger, 2003 | Walnut Creek, Iowa | Corn | Measurements | Growing season (May-August) | 210 - 240 | 385 - 425 (390) | Unspecified | [Link](http://cdn.intechopen.com/pdfs-wm/22687.pdf) |
| Serbina & Miller, 2014 | Eastern Colorado | Corn | Measurements; METRIC model | Growing season (May-Sept) | 515 - 570 | 325 - 675 (450) | Yes | [Link](http://pubs.usgs.gov/of/2014/1108/pdf/ofr2014-1108.pdf) |
| Medellín-Azuara & Howitt, 2013 | Sac.-San Joaquin, CA | Corn | SEBAL; simulation models | Growing season (March-Sept) | 660 - 830 | 600 - 860 (730) | Yes | [Link](http://www.delta.ca.gov/%5C/res/docs/landscapes/UCD_ET_Report_9-2013.pdf) |
| Masoner et al., 2003 | Lake Altus, OK / TX | Corn | Radation method | Growing season (Feb - July) | 730 - 820 | 390 - 565 (440) | Yes | [Link](http://pubs.usgs.gov/wri/wri034155/pdf/wri034155.pdf) |
| Howell et al., 1997 | Bushland, Texas | Corn | Measured; multiple models | Growing season (Sept - July) | 700 - 830 | 320 - 565 (400) | Yes |  |
| Kanemasu, 1977a | Kansas | Soybeans | Measurements; simulation model | Growing season (May-August) | 520 | 390 - 500 (410) | Unspecified | [Kansas State](http://www.ksre.ksu.edu/historicpublications/Pubs/SRL32.pdf) |
| Masoner et al., 2003 | Lake Altus, OK / TX | Soybeans | Radation method | Growing season (April - Sept) | 730 - 820 | 450 - 520 (465) | Yes | [Link](http://pubs.usgs.gov/wri/wri034155/pdf/wri034155.pdf) |
| Masoner et al., 2003 | Lake Altus, OK / TX | Alfalfa | Radation method | Growing season (Jan - Dec) | 990 - 1115 | 780 - 1050 (840) | Yes | [Link](http://pubs.usgs.gov/wri/wri034155/pdf/wri034155.pdf) |
| Medellín-Azuara & Howitt, 2013 | Sac.-San Joaquin, CA | Alfalfa | SEBAL; simulation models | Growing season (March-Sept) | 905 - 980 | 400 - 1200 (680) | Yes | [Link](http://pubs.usgs.gov/wri/wri034155/pdf/wri034155.pdf) |
| WPAWS, 2000 | Mount Vernon, WA | Alfalfa | Kimberly-Penman | Growing season (January-Dec) | 770 | 710 - 900 (775) | Unspecified | [USGS](http://wa.water.usgs.gov/projects/wria01/data/refet.htm) |
| Shewmaker et al., 2013 | Kimberly, Idaho | Alfalfa | Kimberly-Penman | Growing season (April - Sept) | 900 | 330 - 865 (460) | Yes | [U. of Idaho](http://www.extension.uidaho.edu/forage/Fact%20Sheets/Alfalfa%20Irrigation%20Facts%202013%20Final%5B1%5D.pdf) |
| Serbina & Miller, 2014 | Eastern Colorado | Alfalfa | Measurements; METRIC model | Growing season (April - Oct) | 660 - 680 | 530 - 840 (560) | Yes | [Link](http://pubs.usgs.gov/of/2014/1108/pdf/ofr2014-1108.pdf) |
| Samis & Smeal, 1983 | Clovis, New Mexico | Winter wheat | Measured; multiple models | Growing season (October - June) | 285 - 615 (445) | 300 - 420 (325) | Unspecified |  |
| Jensen & Sletten, 1965 | Bushland, Texas | Winter wheat | Measurements | Growing season (October - July) | 450 - 710 | 300 - 865 (435) | Yes |  |
| Howell et al., 1997 | Bushland, Texas | Winter wheat | Measured; multiple models | Growing season (Sep - July) | 790 - 930 | 300 - 865 (435) | Yes |  |
| Masoner et al., 2003 | Lake Altus, OK / TX | Winter wheat | Radation method | Growing season (August - Dec) | 660 - 1070 | 380 - 900 (440) | Yes | [Link](http://pubs.usgs.gov/wri/wri034155/pdf/wri034155.pdf) |
| Kanemasu, 1977a | Kansas | Winter wheat | Measurements; simulation model | Growing season (August - Dec) | 380 - 480 | 325 - 450 (350) | Unspecified | [Kansas State](http://www.ksre.ksu.edu/historicpublications/Pubs/SRL32.pdf) |
| Kanemasu, 1977b | Manhattan, Kansas | Winter wheat | Measurements; Landsat model | Growing season (August - Dec) | 410 - 490 (430) | 460 - 500 (470) | Unspecified |  |
| McAuley et al., 1978 | Goodwell, Oklahoma | Sorghum | Measurements | Growing Season (June - Nov) | 330 - 760 | 550 - 630 (570) | Yes |  |
| Masoner et al., 2003 | Lake Altus, OK / TX | Sorghum | Radation method | Growing season (April - Sept) | 650 - 730 | 415 - 500 (430) | Yes | [Link](http://pubs.usgs.gov/wri/wri034155/pdf/wri034155.pdf) |
| Kanemasu, 1997a | Kansas | Sorghum | Measurements; PEM | Growing season (June-Sept) | 500 | 330 - 610 (385) | Unspecified | [Link](http://www.ksre.ksu.edu/historicpublications/Pubs/SRL32.pdf) |
| Zeng & Heilman, 1997 | West Texas (Lubbock) | Sorghum | ENWATBAL simulation model | Growing season (March-August) | 565 | 460 - 550 (475) | Unspecified | [Article](http://link.springer.com/article/10.1007%2FBF00863616) |
| Howell et al., 1997 | Bushland, Texas | Sorghum | Measured; multiple models | Growing season (Sept - July) | 535 - 640 | 460 - 550 (475) | Yes |  |
| Masoner et al., 2003 | Lake Altus, OK / TX | Cotton | Radation method | Growing season (Feb - July) | 850 - 960 | 660 - 770 (680) | Yes | [Link](http://pubs.usgs.gov/wri/wri034155/pdf/wri034155.pdf) |
| Zeng & Heilman, 1997 | West Texas (Lubbock) | Cotton | ENWATBAL simulation model | Growing season (Feb - July) | 360 | 130 - 500 (400) | No | [Link](http://link.springer.com/article/10.1007%2FBF00863616) |
| Zeng & Heilman, 1997 | West Texas (Lubbock) | Cotton | ENWATBAL simulation model | Growing season (Feb - July) | 415 | 360 - 800 (530) | Yes | [Link](http://link.springer.com/article/10.1007%2FBF00863616) |
| Fisher, 2012 | Stoneville, Mississippi | Cotton | Lysimeter; Penman | Growing season (April-Sept) | 500 - 570 | 415 - 500 (430) | Yes |  |
| Fisher, 2012 | Stoneville, Mississippi | Cotton | Penman-Monteith | Growing season (April-Sept) | 615 - 740 | 415 - 500 (430) | Yes |  |
| Serbina & Miller, 2014 | Eastern Colorado | Sugar beets | Measurements; METRIC model | Growing season (April - October) | 530 - 575 | 380 - 650 (445) | Yes |  |
| Samis & Smeal, 1983 | Clovis, New Mexico | Spring barley | Measured; multiple models | Growing season (May - Sept) | 335 - 525 (400) | 300 - 800 (500) | Yes | [Link](http://www.wrri.nmsu.edu/publish/techrpt/tr179/tr179.pdf) |
| Robertson & Stark, 2003 | Southern Idaho | Spring barley | Measured (estimated mean) | Growing season (April - Sept) | 650 | 700 - 900 (755) | Yes | [U. of Idaho](http://www.extension.uidaho.edu/forage/Fact%20Sheets/Alfalfa%20Irrigation%20Facts%202013%20Final%5B1%5D.pdf) |
| Serbina & Miller, 2014 | Eastern Colorado | "small grains" | Measurements; METRIC model | Growing season (April - August) | 435 - 445 | 300 - 800 (500) | Unspecified | [Link](http://pubs.usgs.gov/of/2014/1108/pdf/ofr2014-1108.pdf) |
| Masoner et al., 2003 | Lake Altus, OK / TX | Peanuts | Radation method | Growing season | 530 - 690 | 450 - 640 (540) | Yes | [Link](http://pubs.usgs.gov/wri/wri034155/pdf/wri034155.pdf) |
| McAuley et al., 1978 | Fort Cobb, Oklahoma | Peanuts | Measurements | Growing Season (May - October) | 435 - 615 | 450 - 610 (520) | Yes |  |
| Sumner & Jacobs, 2005 | Ferris farms, Florida | Grassland pasture | Measured; multiple models | Annual | 690 - 900 | 640 - 910 (740) | No |  |
| Douglas et al., 2009 | Florida | Grassland pasture | Measured; multiple models | Annual | 675 - 1090 | 660 - 1000 (735) | No |  |
| Serbina & Miller, 2014 | Eastern Colorado | Grassland pasture | Measurements; METRIC model | Annual | 565 - 670 | 215 - 580 (250) | No | [Link](http://pubs.usgs.gov/of/2014/1108/pdf/ofr2014-1108.pdf) |

Seasonal and annual evapotranspiration ranges reported in literature sources and model by *CropWatR*. A range of methods are available for determining evapotranspiration at the field, landscape, and watershed scales, either via direct measurement (e.g. soil moisture measurement via lysimeters), modeling (e.g. via process-based s imulation models, or energy balances using weather data collected via satellite, local instruments, and/or remote sensing). For a summary of common methods, see Connor et al.,2011 [24].
