# Supplementary material for: Water impacts of U.S. biofuels: Insights from an assessment combining economic and biophysical models
Source: PLoS One. 2018 Sep 28;13(9):e0204298. doi: 10.1371/journal.pone.0204298 (PMC6161887; doi:10.1371/journal.pone.0204298)
Supplement: S2 File — Table A. Kcb parameter values for perennial grassland and non-crop agricultural land cover types. Table B. Crop water balances that can be estimated for daily, seasonal, or annual time steps. Table C. Literature estimates of evapotranspiration versus modeled evapotranspiration rates. Seasonal and annual evapotranspiration ranges reported in literature sources and model by CropWatR. A range of methods are available for determining evapotranspiration at the field, landscape, and watershed scales, either via direct measurement (e.g. soil moisture measurement via lysimeters), modeling (e.g. via process-based s imulation models, or energy balances using weather data collected via satellite, local instruments, and/or remote sensing). For a summary of common methods, see Connor et al.,2011. Table C. Model performance metrics comparing the results with MODIS 16 estimates. Table E. NASS Classification categories considered in this analysis. Note that double-cropped classifications where both crops were not included among the 14 parameterized crops (e.g. lettuce / upland cotton, lettuce / barley, etc.) were excluded from the analysis. The total acreage of these classifications on the national scale was in all cases much less than 1% of the acreage of the modeled crop. Table F. NASS Accuracy assessments for crops and land types considered in this analysis. Accuracy statistics for crop and land use categories not reported in the above table are not available for 2008. These categories are: alfalfa, other hay, sugarcane, fallow/idle cropland, grassland herbaceous, and pasture/hay. Fig A. Relation between time (day since emergence), crop coefficient (Kcb) and plant height for maize. Crop coefficients are specified for three moments in time. According to the FAO 56 methods, coefficients are estimated by stepwise and linear interpolation (black line). In CropWatR, a Bezier curve (red curve) is used to interpolate daily Kcb values. Fig B. Irrigation calibration results for corn. The [file pone.0204298.s002.zip › S2 File Figures Tables/S2 Table D.docx]

**S2 Table D. Model performance metrics comparing the results with MODIS 16 estimates.**

| Crop | # obs. | Accuracy | Correlations | | Bias | Reliability | | Efficiency |
| --- | --- | --- | --- | --- | --- | --- | --- | --- |
|  |  | annual | 8-day measure | annual | annual | 8-day | annual | 8-day measure |
| Corn | 327 | 0.02% - 15.5% | 0.85 (0.72 - 0.93) | 0.77 | 4.90% | 0.44 | 1 | 0.70 (0.33 - 0.86) |
| Soybeans | 122 | 0.3% - 15.3% | 0.84 (0.69 - 0.91) | 0.77 | 5.90% | 0.41 | 1 | 0.68 (0.37 - 0.83) |
| Alfalfa | 84 | 0.3% - 20.7% | 0.83 (0.61 - 0.91) | 0.78 | 7.7% | 0.51 | 0.940 | 0.63 (0.14 - 0.83) |
| Pasture grass | 38 | 0.03% - 19.0% | 0.80 (0.48 - 0.93) | 0.82 | 9.39% | 0.55 | 1 | 0.53 (-0.26 - 0.84) |
| Durum wheat | 3 | 1.5% - 21.2% | 0.75 (0.71 - 0.79) | 0.92 | 8.87% | 0.34 | 0.667 | 0.77 (0.70 - 0.84) |
| Spring wheat | 9 | 0.1% - 12.6% | 0.58 (0.49 - 0.78) | 0.96 | 12.23% | 0.30 | 1 | 0.14 (0.03 - 0.28) |
| Spring barley | 8 | 0.5% - 16.6% | 0.65 (0.52 - 0.83) | 0.91 | 8.17% | 0.39 | 1 | 0.30 (0.06 - 0.64) |
| Rice | 12 | 5.08% - 20.2% | 0.69 (0.58 - 0.76) | 0.55 | 11.54% | 0.27 | 0.917 | 0.30 (0.10 - 0.47) |
| Peanuts | 15 | 0.37% - 17.8% | 0.68 (0.57 - 0.83) | 0.69 | 8.14% | 0.37 | 1 | 0.25 (0.03 - 0.63) |
| Cotton | 35 | 0.10% - 16.0% | 0.79 (0.68 - 0.91) | 0.86 | 7.83% | 0.40 | 1 | 0.52 (0.14 - 0.82) |
| Spring oats | 8 | 1.2% - 15.3% | 0.71 (0.56 - 0.87) | 0.52 | 8.43% | 0.40 | 1 | 0.29 (0.01 - 0.64) |
| Sorghum | 39 | 0.13% - 19.1% | 0.75 (0.56 - 0.90) | 0.97 | 8.87% | 0.33 | 1 | 0.36 (0.03 - 0.68) |
